# The HIV latency reversing agent HODHBt inhibits the phosphatases PTPN1 and PTPN2

**DOI:** 10.1172/jci.insight.179680

**Published:** 2024-08-08

**Authors:** J. Natalie Howard, Thomas D. Zaikos, Callie Levinger, Esteban Rivera, Elyse K. McMahon, Carissa S. Holmberg, Joshua Terao, Marta Sanz, Dennis C. Copertino, Weisheng Wang, Natalia Soriano-Sarabia, R. Brad Jones, Alberto Bosque

**Affiliations:** 1 Department of Microbiology, Immunology, and Tropical Medicine, George Washington University, Washington, DC, USA.; 2 Department of Pathology, Johns Hopkins Hospital, Baltimore, Maryland, USA.; 3 Department of Medicine, Weill Cornell Medical College, New York, New York, USA.

**Keywords:** AIDS/HIV, Cytokines, Phosphoprotein phosphatases, Signal transduction

## Abstract

Nonreceptor tyrosine phosphatases (NTPs) play an important role in regulating protein phosphorylation and have been proposed as attractive therapeutic targets for cancer and metabolic diseases. We have previously identified that 3-Hydroxy-1,2,3-benzotriazin-4(3H)-one (HODHBt) enhanced STAT activation upon cytokine stimulation, leading to increased reactivation of latent HIV and effector functions of NK and CD8 T cells. Here, we demonstrate that HODHBt interacted with and inhibited the NTPs PTPN1 and PTPN2 through a mixed inhibition mechanism. We also confirm that PTPN1 and PTPN2 specifically controlled the phosphorylation of different STATs. The small molecule ABBV-CLS-484 (AC-484) is an active site inhibitor of PTPN1 and PTPN2 currently in clinical trials for advanced solid tumors. We compared AC-484 and HODHBt and found similar effects on STAT5 and immune activation, albeit with different mechanisms of action leading to varying effects on latency reversal. Our studies provide the first specific evidence to our knowledge that enhancing STAT phosphorylation via inhibition of PTPN1 and PTPN2 is an effective tool against HIV.

## Abstract 2

We describe the cellular targets of a small molecule with potential use to eliminate latent reservoirs towards finding an HIV cure

## Graphical abstract

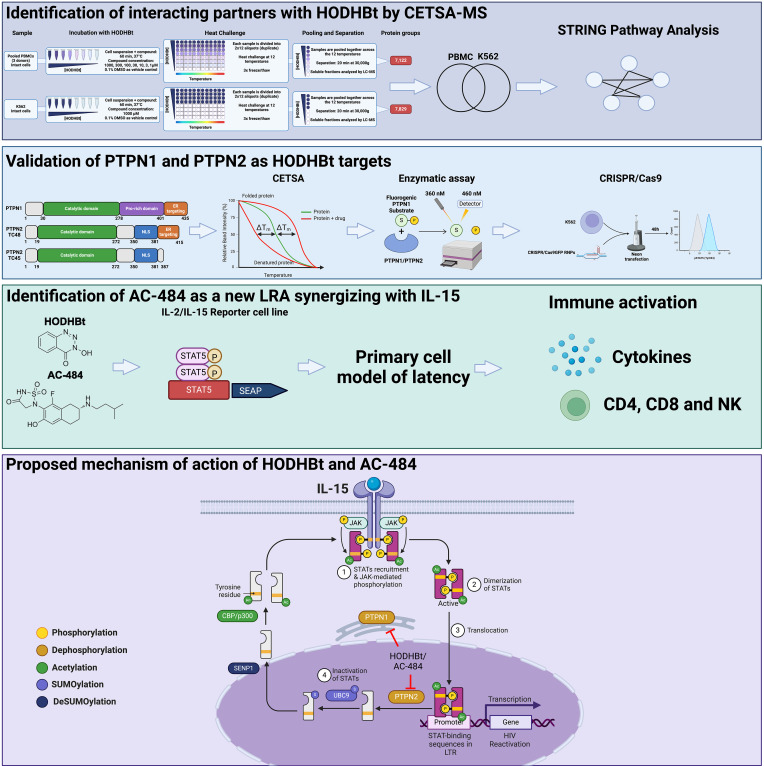

## Introduction

Despite the development and rapid advancement of antiretroviral therapy (ART) against HIV in the past 3 decades, there remains no functional cure. This is due to the presence of an intact and inducible provirus that is integrated within infected cells and is unseen by the immune system, allowing for viral persistence despite ART (1–3). Efforts to identify small molecules that can reactivate (or shock) latent viruses into active replication, allowing cells to be seen and eliminated by immune effector cells, remains at the forefront of research today.

We have previously described that the small molecule 3-Hydroxy-1,2,3-benzotriazin-4(3H)-one (HODHBt) is able to enhance cytokine-mediated STAT signaling (4). We initially identified HODHBt via a screening for compounds that could reactivate latent HIV in a primary cell model of latency (5). Our previous studies demonstrate that HODHBt was able to increase cytokine-induced phosphorylated STAT5 (pSTAT5), leading to enhanced binding of pSTAT5 to the HIV long terminal repeat (LTR). This resulted in viral transcriptional activation and latency reversal in primary CD4 T cells (4). We then described that the structural analogue 1,2,3-Benzotriazine-4(3H)-one (HBt) lacks biological activity, indicating the importance of the 3-hydroxy group in the biological activity of these compounds. Furthermore, we demonstrated that HODHBt lacks acute toxicity in mice and does not promote global immune activation (6). In follow-up studies, we showed that HODHBt enhanced the ability of IL-15 to (a) promote IFN-γ and Granzyme B production in NK cells leading to increased cytotoxic activity against HIV-infected cells and cancer cell lines (7) and to (b) enhance the cytotoxic activity of HIV-specific CD8 T cells via increasing the expression Granzyme B in CD8 T cells and MHC-I expression on target cells (8). However, the direct targets of HODHBt remain unknown.

Here, to identify HODHBt target candidates, we used thermal proteomic profiling (TPP) (9–11). The 2 top hits were the nonreceptor tyrosine phosphatases (NTPs) protein tyrosine phosphatase nonreceptor type 1 (PTPN1) and type 2 (PTPN2), known for their activity in the regulation of the STAT signaling pathway (12–15). Utilizing biochemical and functional assays, we determined that HODHBt is a mixed inhibitor of PTPN1 and PTPN2. Recently, a small molecule dual active site inhibitor of PTPN1 and PTPN2, ABBV-CLS-484 (AC-484, Osunprotafib), has been characterized as a potent immune activator of antitumor responses (16), and there is 1 clinical trial in progress determining the effects of AC-484 against advanced solid tumors (NCT04777994; https://clinicaltrials.gov/study/NCT04777994). In this work, we compared the anti-HIV functions of these 2 PTPN1/PTPN2 inhibitors, HODHBt and AC-484. We showed that HODHBt and AC-484 have similar effects on STAT5 phosphorylation, induction of STAT5 transcriptional activity, and immune cell activation but differ in their ability to reactivate latent HIV in primary cells. These results show that PTPN1 and PTPN2 can be targeted to reverse latency, broadening current approaches for HIV cure.

## Results

### HODHBt modulates the thermal stability of PTPN1 and PTPN2.

**Figure 1:**
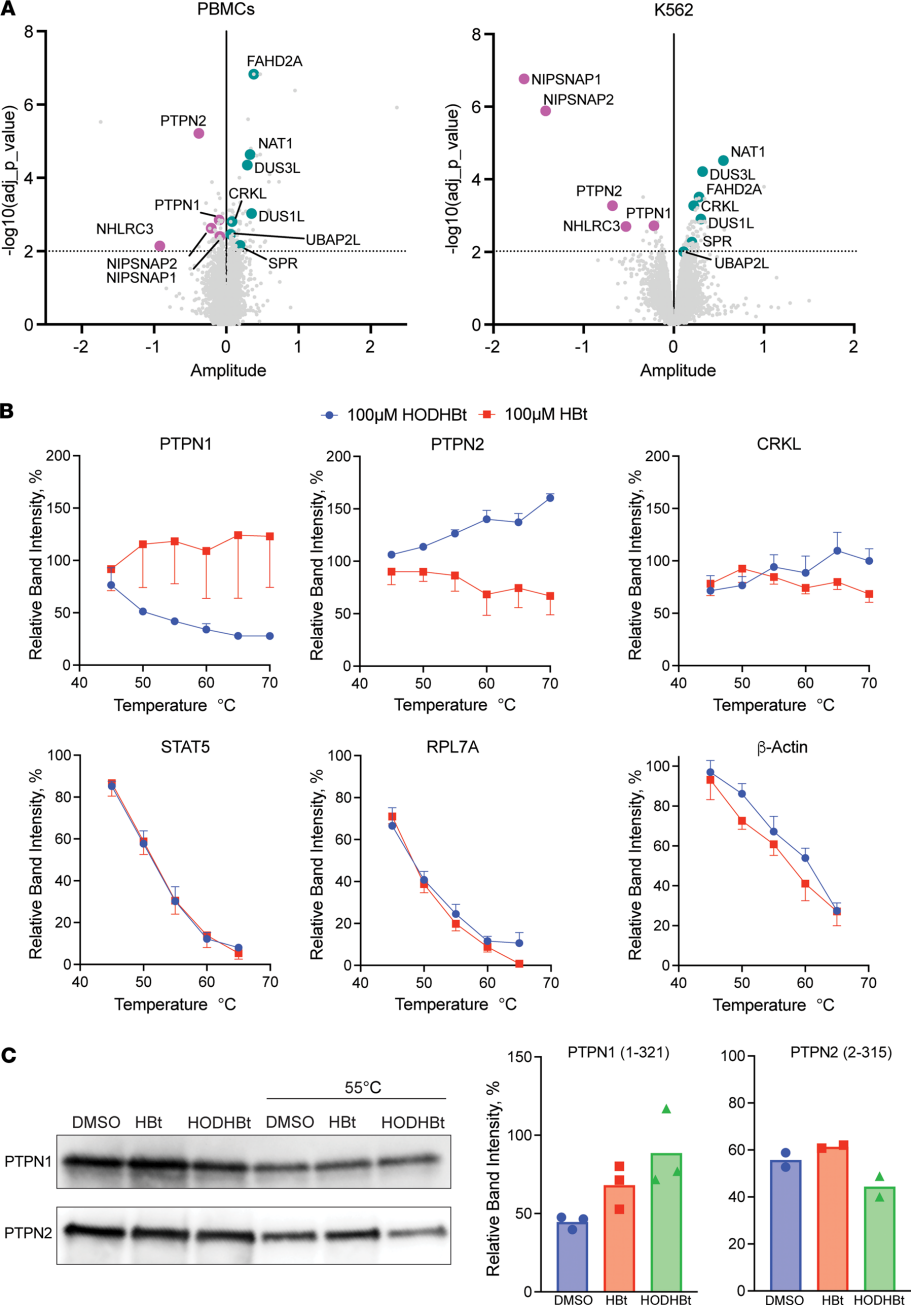
HODHBt modulates the thermal stability of PTPN1 and PTPN2 in vitro. (**A**) Compressed CETSA-MS results indicating changes in thermal stability of protein in both PBMCs and K562. The *x* axis represents amplitude (log_2_ fold change), and the *y* axis represents effect significance (–log_10_ [*P* value]). Statistically significant (*P* < 0.01) proteins in common between PBMCs and K562 cells are indicated. (**B**) Thermal melting curves for PTPN1, PTPN2, CRKL, STAT5, RPL7A, and β-actin in CD4 T cell lysates after treatment with 100 μM HODHBt versus 100 μM HBt (*n* = 2–4). (**C**) Purified PTPN1 catalytic domain and purified commercial PTPN2 catalytic domain CETSA after treatment with DMSO, 100 μM HODHBt, or 100 μM HBt (*n* = 2–3).

TPP couples the cellular thermal shift assay (CETSA) with quantitative mass spectrometry (MS) (9–11), allowing precise identification of proteins that bind to a small molecule by identifying changes in protein stabilization of thousands of proteins simultaneously upon heating. TPP was performed in live peripheral blood mononuclear cells (PBMCs) from HIV^–^ donors and the chronic myelogenous leukemia cell line K562 cells treated with HODHBt (11, 17, 18). By performing TPP in living cells as opposed to cell lysates, we ensured that the targets were (a) present at their physiological levels; (b) with their posttranslational modifications; (c) in their subcellular compartments; and (d) interacting with other proteins in their native conformation. We reliably quantified changes in stabilization of 7,122 and 7,829 proteins in PBMCs and K562 cells, respectively (Supplemental Data File 1; supplemental material available online with this article; https://doi.org/10.1172/jci.insight.179680DS1). HODHBt only changed the thermal stability of 119 proteins in PBMCs and 173 proteins in K562 cells (*P* < 0.01) (Supplemental Data File 2). Among those, only 12 proteins were shared between both cell types (Figure 1A, Supplemental Figure 1, and Supplemental Data File 2). Next, STRING pathway analysis identified 3 proteins with known interactions with STAT5: PTPN1, PTPN2, and CRKL (Supplemental Data File 3). PTPN1 and PTPN2 are 2 NTPs that regulate STAT5 activation, with PTPN1 being the predominant phosphatase present in the endoplasmic reticulum (ER) and with PTPN2 being present in both the ER and nucleus (19, 20). CRKL is a proto-oncogene adaptor protein that has been shown to directly interfere with STAT5-DNA binding (21). To validate the TPP, we measured changes induced by HODHBt in thermal stabilization of different proteins in primary CD4 T cell lysates (10). Confirming our TPP, HODHBt specifically induced changes in the thermal stability of PTPN1 and PTPN2 compared with the inactive analogue HBt (Figure 1B and Supplemental Figure 2). We could not confirm changes in the thermal stability of CRKL. As controls, we evaluated changes in the thermal stability of STAT5, the ribosomal protein RPL7A, or the housekeeping gene β-actin, which did not show changes in thermal stability in our TPP (Supplemental Data File 1). We did not observe changes in the thermal stability of these 3 proteins (Figure 1B). Additionally, we measured changes in the thermal stability of purified PTPN1 and PTPN2 catalytic domain proteins induced by HODHBt and HBt compared with a DMSO control. For PTPN1, we observed stabilization by both HODHBt and the inactive control HBt compared with DMSO, whereas for PTPN2, we observed destabilization with HODHBt but not HBt (Figure 1C). These data suggest that the inactive control HBt could also bind to PTPN1 but does not have the downstream effects of HODHBt. Next, to confirm the role of PTPN1 and PTPN2 regulating STAT5 phosphorylation (22, 23), both NTPs were individually or simultaneously knocked out using CRISPR-Cas9 in K562. We confirmed that knocking out both PTPN1 and PTPN2 results in enhanced STAT5 phosphorylation, suggesting that both NTPs are equally important in STAT5 regulation (Supplemental Figure 3, A–C). Additionally, we observed that treatment of K562 cells with HODHBt resulted in a dose-dependent increase in pSTAT5 concomitant with a reduction on the levels of PTPN2 and, to a lesser extent, of PTPN1 (Supplemental Figure 3D), suggesting that HODHBt may promote changes in the expression of these 2 phosphatases.

### HODHBt is a mixed inhibitor of PTPN1 and PTPN2.

**Figure 2:**
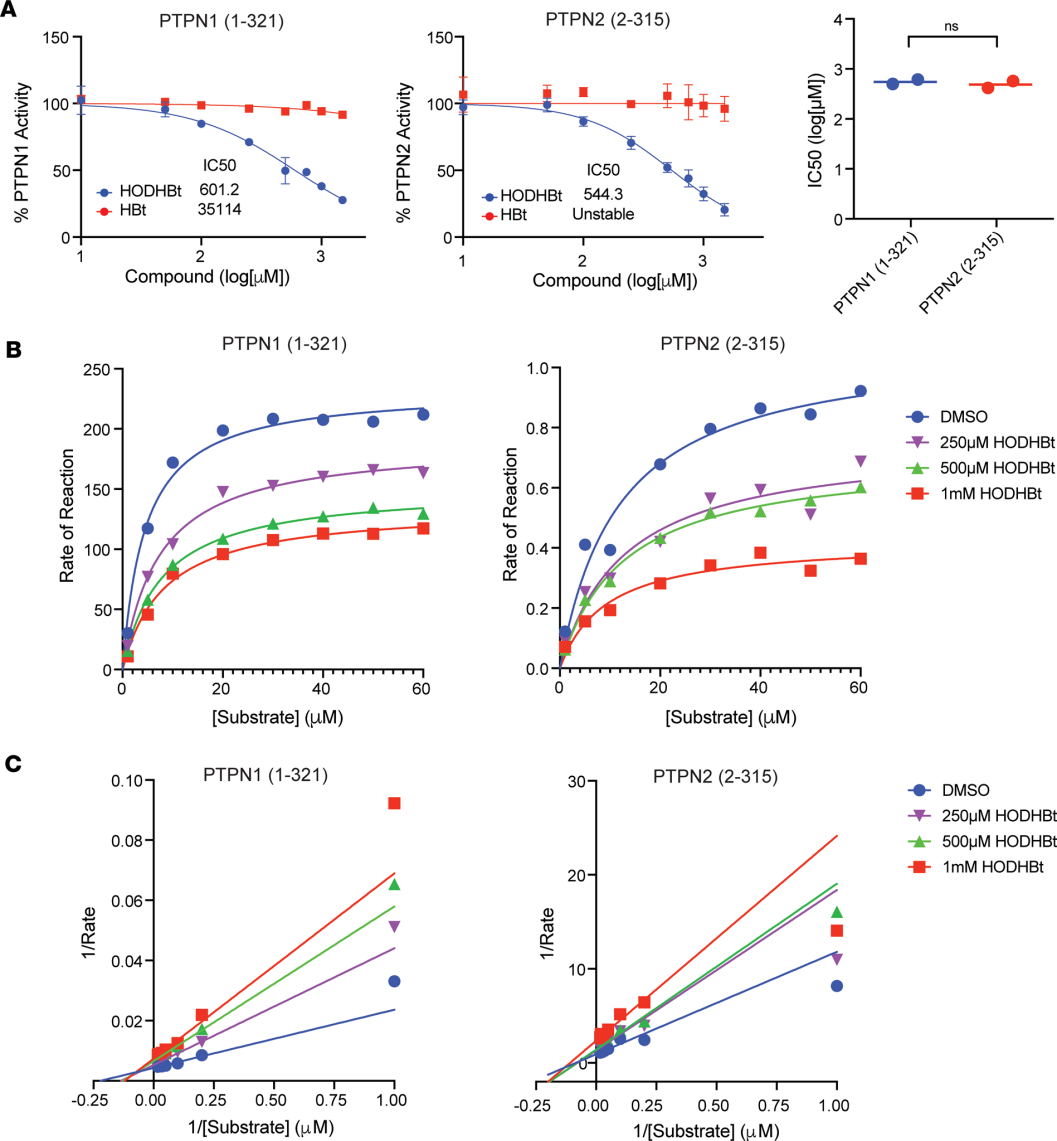
HODHBt is a mixed inhibitor of PTPN1 and PTPN2. (**A**) HODHBt directly inhibits the catalytic activity of the catalytic domain of PTPN1and the catalytic domain of PTPN2 using a fluorogenic assay (*n* = 2). Data are shown as mean ± SD. IC_50_ values calculated for 2 independent experiments. (**B**) Effect of HODHBt on PTPN1- and PTPN2-catalyzed fluorogenic PTPN1 substrate. (**C**) Lineweaver-Burk plots. Lineweaver-Burk plots represent the Michaelis-Menten equation of enzyme kinetics.

PTPN1 and PTPN2 belong to the Class I PTP family and share an overall 72% sequence similarity and 94% similarity for the catalytic domain, including the cysteine residue required for full enzymatic function (24, 25) (Supplemental Figure 4). Using recombinant PTPs in vitro, we characterized HODHBt’s mechanism of inhibition. HODHBt inhibited the catalytic activities of both PTPN1 and PTPN2 compared with the inactive structural analogue HBt with average IC_50_ values of 601 μM and 544 μM, respectively (Figure 2A). To determine the mechanism of inhibition, we performed a kinetic analysis using varying concentrations of both the substrate and HODHBt (Figure 2B). This allowed us to perform Michaelis-Menten least square fit analysis to determine the V_max_ and *K_m_* for each HODHBt concentration, where V_max_ is the extrapolated maximum enzyme velocity and *K_m_* is the substrate concentration needed to achieve a half-maximum enzyme velocity (also known as the Michaelis-Menten constant) (Supplemental Figure 5). Using the calculated V_max_ and *K_m_* values, we fitted the data to Lineweaver-Burk plots and determined that HODHBt is a mixed inhibitor. A mixed inhibitor is defined as an inhibitor that can either bind to the enzyme at an allosteric site regardless of whether the substrate is bound or can bind at an allosteric site to the already-bound enzyme-substrate complex (26, 27). Both scenarios result in a decrease in the V_max_, but preferential binding of the inhibitor to free enzyme increases the *K_m_*, while binding to the enzyme-substrate complex decreases the *K_m_* (27). Our results suggest potential preferential binding of HODHBt to free PTPN1 and PTPN2 substrate complex based on the increased and decreased *K_m_* values, respectively (Figure 2C and Supplemental Figure 5). Together, our results demonstrate that HODHBt binds and inhibits the catalytic domain of PTPN1 and PTPN2, and they describe a class of compounds that act as dual PTPN1/PTPN2 mixed inhibitors.

### PTPN1 and PTPN2 control the phosphorylation and transcriptional activity of STATs in a cytokine-specific manner.

**Figure 3:**
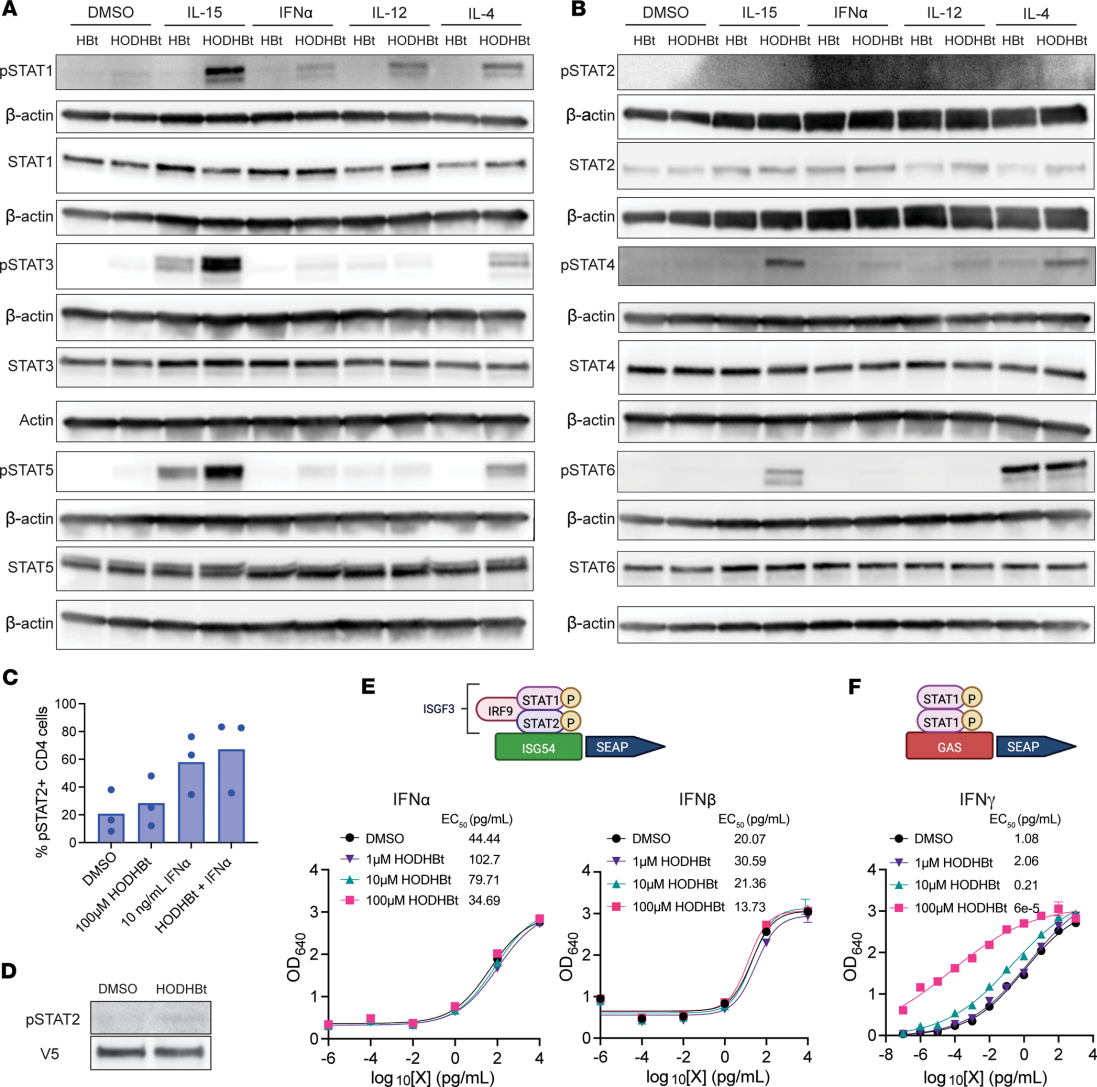
Inhibiting PTPN1 and PTPN2 with HODHBt enhances activation of different STATs in a cytokine-dependent manner. (**A** and **B**) Analysis of phosphorylation levels of STAT-1, -3, and- 5 (**A**) or STAT-2, -4, and -6 (**B**) in primary total CD4 T cells after treatment with DMSO, 100 ng/mL IL-15, 1 ng/mL IFN-α, 2 ng/mL IL-12, and 2 ng/mL IL-4 in the presence of 100 μM HODHBt or HBt for 24 hours. (**C**) Levels of pSTAT2^+^ cells in naive CD4 T cells treated with 100 μM HODHBt ± 10 ng/mL IFN-α for 24 hours (*n* = 3). (**D**) Levels of STAT2 phosphorylation after transfection of V5-STAT2 into 293FT cells and treatment with DMSO or 100 μM HODHBt. (**E**) Dose response of STAT1/2 transcriptional activity mediated by IFN-α (left) and IFN-β (right) in the presence of 1, 10, or 100 μM HODHBt in HEK-Blue IFN-α/β cells. The data represent the mean ± SD of an experiment performed in triplicate. (**F**) Dose response of STAT1 transcriptional activity mediated by IFN-γ in the presence of 1, 10, or 100 μM HODHBt in HEK-Blue IFN-γ cells. The data represent the mean ± the SD of an experiment performed in triplicate.

We have reported previously that, in addition to enhancing STAT5A and STAT5B phosphorylation, HODHBt also enhances the phosphorylation of STAT1 and STAT3 upon IL-15 stimulation (6, 7). To test whether inhibiting PTPN1 and PTPN2 with HODHBt enhanced phosphorylation of additional STATs, we isolated and treated primary total CD4 T cells with 4 different cytokines targeting activation of 1 or more specific STATs for 24 hours (28). As previously reported, HODHBt alone did not significantly increase STAT phosphorylation (4). However, stimulation with IL-15 combined with HODHBt resulted in increased phosphorylation of STAT1, STAT3, STAT4, and STAT5 compared with the inactive control HBt. HODHBt also enhanced IFN-α–mediated STAT1 phosphorylation but not STAT2 (Figure 3, A and B). We confirmed that HODHBt does not enhance phosphorylation of STAT2 in total CD4 T cells in the presence of IFN-α (Figure 3C) and in 293FT cells stably transfected with V2-tagged STAT2 (Figure 3D). Stimulation with IL-12 in the presence of HODHBt resulted in increased phosphorylation of STAT1, while HODHBt did not influence IL-4–mediated STAT6 phosphorylation (Figure 3, A and B). Failure of HODHBt to enhance STAT2 phosphorylation suggests that PTPN1 and PTPN2 might not control STAT2 transcriptional activity. STAT2, in combination with STAT1, are important for regulating IFN signaling. While type-I IFN-α/β requires STAT1 and STAT2 heterodimers and binding to IFN-sensitive response element (ISRE), type II IFN-γ signals through STAT1 homodimers and gamma interferon activation site (GAS) elements (29–31). Based on our previous work, we hypothesized that inhibiting PTPN1 and PTPN2 with HODHBt would enhance IFN-γ but not IFN-α/β. We confirmed that inhibiting PTPN1 and PTPN2 with HODHBt did not enhance IFN-α or IFN-β activation of the ISRE promoter (Figure 3E). On the other hand, HODHBt enhanced IFN-γ activation of the GAS promoter in a dose-dependent manner (Figure 3F). These data suggest that PTPN1 and PTPN2 regulate the phosphorylation of all STAT isoforms except STAT2 and STAT6. This could be attributed to the fact that both PTPN1 and PTPN2 preferentially bind to biphosphorylated substrates (32). All STATs, excluding STAT2 and STAT6, have a conserved serine residue that can be phosphorylated in addition to the ubiquitous C-terminal tyrosine residue required for SH2 partner interaction and dimerization (33). Overall, our findings confirm that PTPN1 and PTPN2 control the phosphorylation and transcriptional activity of STAT-1, -3, -4, and -5 and that HODHBt enhances STAT transcriptional activation in a cytokine-specific manner.

### AC-484 promotes immune activation and synergizes with IL-15 to reactivate latent HIV.

**Figure 4:**
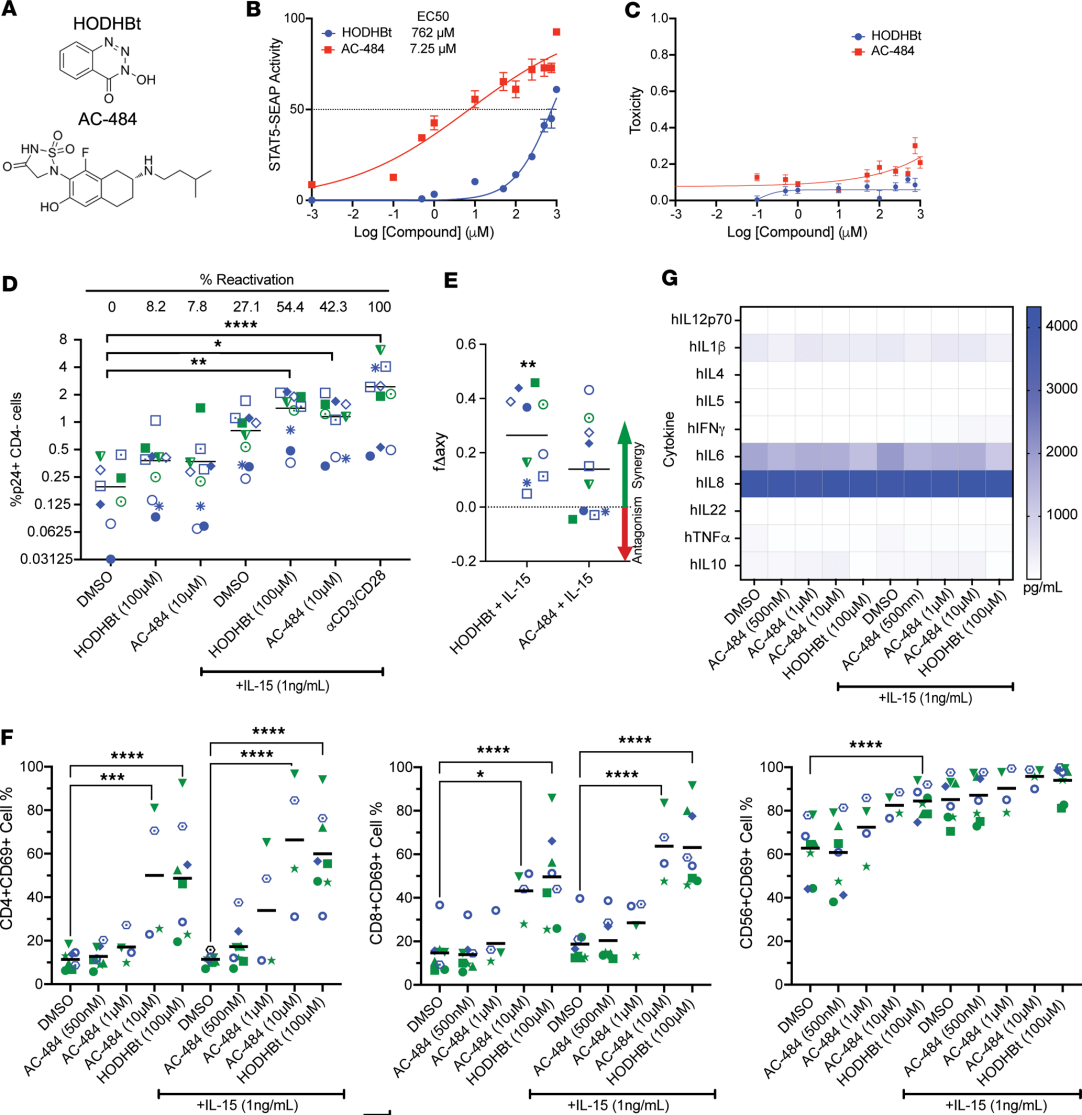
AC-484 promotes immune activation and synergizes with IL-15 to reactivate latent HIV. (**A**) Structure comparison of HODHBt and AC-484. (**B** and **C**) Measurement of STAT5 transcriptional activity (**B**) and toxicity (**C**) after treatment with dose response of HODHBt and AC-484 in HEK–Blue–IL-2/IL-15 cells. The data represent the mean ± SD of an experiment performed in duplicate. (**D**) Reactivation of latent HIV in Tcm cells measured by flow cytometry after treatment with 100 μM HODHBt or 10 μM AC-484 ± 100 ng/mL IL-15, or αCD3/CD28 (*n* = 10). Dunnett’s multiple-comparison test was used to calculate *P* values (*P < 0.05; **P < 0.01; ****P < 0.0001). (**E**) Bliss independence synergy calculations for reactivation. Wilcoxon matched-pairs signed-rank test was used to calculate *P* values (**P < 0.01). (**F**) PBMCs were treated with 100 μM HODHBt and a dose response of AC-484 ± 1 ng/mL IL-15 for 48 hours. CD69 induction was analyzed by flow cytometry in CD4 T cells, CD8 T cells, and NK cells (*n* = 4–8). Data are the average effect from 4–8 donors. Tukey’s multiple-comparison test was used to calculate *P* values (*P < 0.05; ***P < 0.001; ****P < 0.0001). (**G**) Secretion of pro- and antiinflammatory cytokine was measured using a 10-plex cytokine ELISA in supernatants from **F**.

AC-484 is a newly characterized active site dual inhibitor of PTPN1 and PTPN2 with potent antitumor effects (16) that is currently in clinical trials for patients with advanced solid tumors (NCT04777994). We sought to investigate the potential of AC-484 as an HIV LRA compared with HODHBt. Structurally, HODHBt and AC-484 share a core benzene ring but lack other substantial similarities that could explain their shared inhibition of PTPN1 and PTPN2 (Figure 4A). First, we evaluated the effects on STAT5 transcriptional activity using HEK–Blue–IL-2/IL-15 cells as previously described (6). Compared with HODHBt, AC-484 was about 100-fold more potent at increasing STAT5 transcriptional activity, with an EC_50_ of 7.25 μM compared with 762 μM of HODHBt (Figure 4B), and there was no observed toxicity with either compound (Figure 4C). These initial findings suggest that AC-484 would be able to reactivate latent HIV to a similar if not greater degree as HODHBt. Given the current clinical relevance of IL-15 as an LRA (34), we next investigated the LRA efficacy of combining HODHBt and AC-484 with IL-15. We observed that HODHBt alone has minimal LRA activity (8.2% of the maximal stimulus αCD3/28 beads) and observed similar minor significant levels with AC-484 (7.8%). Treatment with IL-15 induced higher frequency of HIV p24^+^ cells compared with the DMSO control (27.1% versus 0%; Figure 4D). Additionally, the combination of HODHBt and IL-15 led to significant reactivation compared with DMSO (54.4% versus 0%, *P* = 0.003; Figure 4D) and synergistic viral reactivation compared with IL-15 alone (Figure 4E). AC-484 with IL-15 also resulted in significantly higher reactivation than the DMSO control (42.3% versus 0%, *P* = 0.03) and was synergistic with IL-15, albeit to a lesser degree than HODHBt (Figure 4, D and E). Similar results were observed with IL-2, though the degree of reactivation was lower than with IL-15 (Supplemental Figure 6). These results show that AC-484 can enhance the LRA activity of IL-15.

We have previously shown that HODHBt in the absence of exogenous cytokine is sufficient to induce immune activation of multiple cell subsets in PBMCs from HIV^–^ individuals and aviremic people living with HIV (PWH) (6). AC-484 has also been shown to increase CD69 expression on T cells in whole blood in a dose-dependent manner (16). To investigate whether AC-484 induces immune activation of CD4 T, CD8 T, and NK cells, we performed dose response experiments of AC-484 in PBMCs with and without low-dose IL-15 and compared the effects to HODHBt. We used AC-484 concentrations from 500 nM to 10 μM and compared the effects of these concentrations with 100 μM HODHBt alone and in the presence of 1 ng/mL of IL-15. In the absence of cytokine, AC-484 induced the expression of CD69 in CD4 T, CD8 T, and NK cells in a dose-dependent manner reaching similar levels as 100 μM HODHBt at the highest concentration tested (10 μM) (Figure 4F). Similar results were observed in the presence of IL-15, but the magnitude of the response was increased compared with no cytokine treatment. Next, we sought to further investigate the effects of AC-484 on immune activation and production of proinflammatory cytokines, a potentially unwanted consequence of manipulating STAT signaling for HIV cure approaches. We have previously shown that HODHBt does not promote the secretion of proinflammatory cytokines (4). On the other hand, AC-484 has been shown to trigger production of the proinflammatory cytokines IFN-γ and TNF-α in mouse T cells (16). Using a 10-plex cytokine ELISA, we saw no significant increase after treatment with HODHBt or any of the AC-484 concentrations alone or with IL-15 of different pro- and antiinflammatory cytokines (Figure 4G). These data show that AC-484 is sufficient to induce immune activation in various cell subsets without inducing a proinflammatory cytokine profile.

### Effects of HODHBt and AC-484 on STAT5 activity.

**Figure 5:**
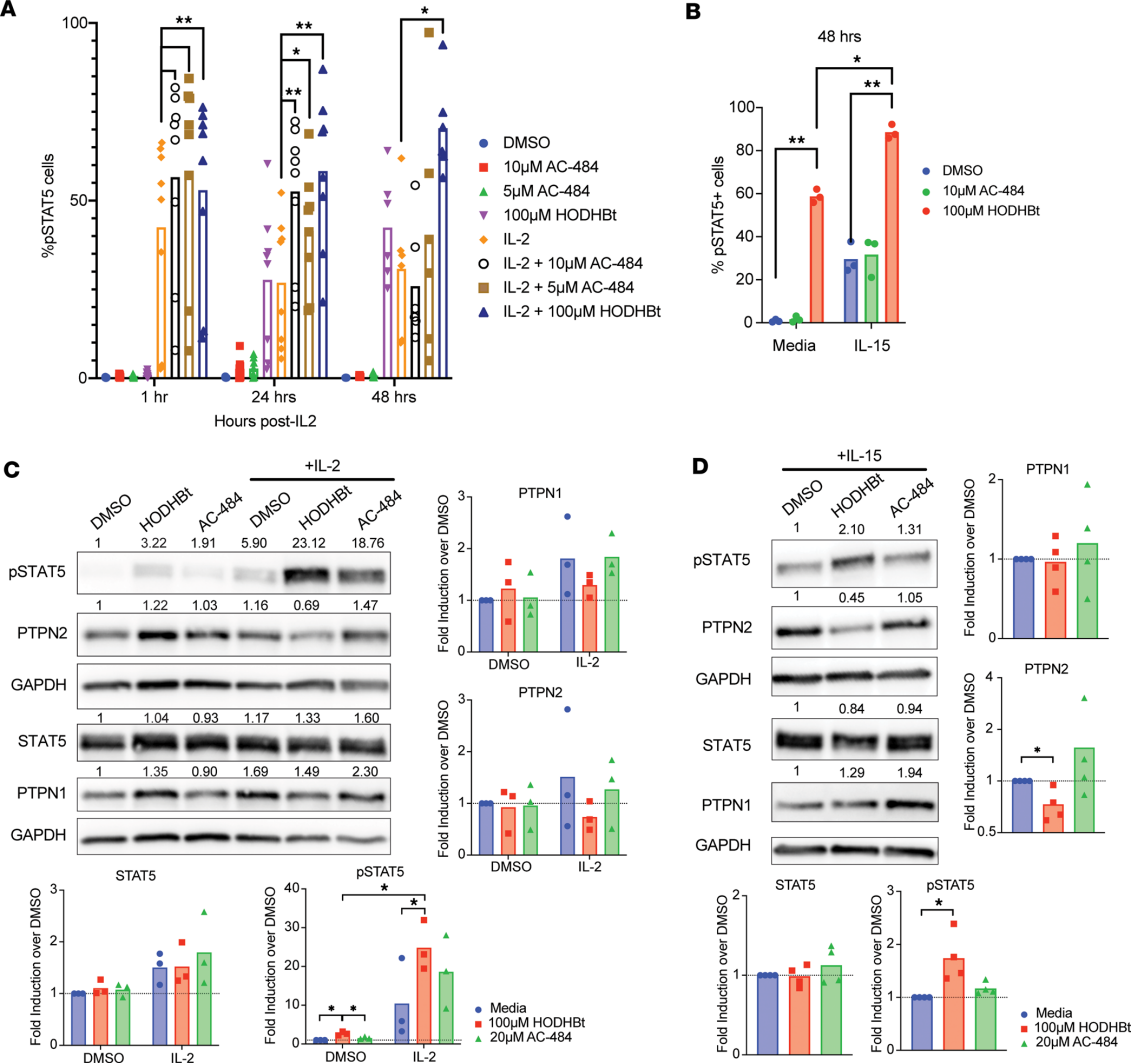
HODHBt and AC-484 have differing effects on STAT5 phosphorylation. (**A**) Total CD4s were pretreated with 100 μM HODHBt, 10 μM AC-484, and 5 μM AC-484 for 2 hours prior to the addition of IL-2. pSTAT5 levels were measured by flow cytometry 1, 24, and 48 hours after stimulation with IL-2 (*n* = 6). Wilcoxon matched-pairs signed-rank test was used to calculate *P* values (*P < 0.05; **P < 0.01). (**B**) Total CD4 T cells were pretreated with 100 μM HODHBt or 10 μM AC-484 for 2 hours prior to the addition of IL-15. pSTAT5 levels were measured by flow cytometry 48 hours after stimulation with IL-2 (*n* = 3). Paired 2-tailed *t* test was used to calculate *P* values (*P < 0.05; **P < 0.01). (**C** and **D**) Protein levels of PTPN1, PTPN2, pSTAT5, and STAT5 were measured by Western blot in CD4 T cells pretreated with 100 μM HODHBt or 20 μM AC-484 for 2 hours before the addition of IL-2 (**C**) and IL-15 (**D**) for 24 hours (*n* = 3). Paired 2-tailed *t* test was used to calculate *P* values (*P < 0.05).

Although AC-484 was a more potent STAT5 activator in the HEK–Blue–IL-2/IL-15 cells, it had lower activity than HODHBt when reactivating latent HIV in a primary cell model of HIV latency. We have previously shown that HODHBt maintains prolonged STAT5 activation upon cytokine signaling, leading to increased nuclear presence (4). We then performed a pSTAT5 time course experiment in primary total CD4 T cells with and without IL-2. After 1 hour and 24 hours in the presence of IL-2, HODHBT and AC-484 induced higher pSTAT5 compared with IL-2 alone. As expected, HODHBt sustained IL-2–mediated pSTAT5 up to 48 hours (Figure 5A). On the other hand, AC-484 was unable to maintain levels of pSTAT5 over IL-2 treatment alone, but this reduction on pSTAT5 levels was not associated with toxicity (Supplemental Figure 7A). Similar results were observed with IL-15 (Figure 5B) without an effect on viability (Supplemental Figure 7B). We have shown that HODHBt is able to reduce the levels of PTPN1 and PTPN2 in K562 cells (Supplemental Figure 3D), potentially explaining the persistent pSTAT5 over time. We then analyzed whether AC-484 was able to reduce PTPN1 and PTPN2 levels. We evaluated changes in both NTP levels after treatment with either HODHBt or AC-484 ± IL-2 in primary CD4 T cells for 24 hours. In the absence of cytokine, we observed no significant changes in the levels of either PTPN1 or PTPN2 after treatment with HODHBt or AC-484 compared with DMSO. However, in the presence of IL-2, we observed a decrease, albeit not significant, in the levels of both PTPN1 and PTPN2 after treatment with HODHBt but not AC-484 compared with IL-2 alone. As expected, we observed a significant increase in pSTAT5 levels after treatment with IL-2 and HODHBt and to a lesser extent with IL-2 and AC-484 compared with IL-2 alone (Figure 5C), confirming the flow cytometry analysis (Figure 5A). In the presence of IL-15, HODHBt treatment similarly resulted in significantly increased pSTAT5 levels and decreased PTPN2 levels, whereas AC-484 again did not enhance pSTAT5 levels or significantly change the levels of PTPN1 and PTPN2 over IL-15 alone (Figure 5D). Together, these data show that AC-484 was not able to sustain pSTAT5 levels over time to the same degree as HODHBt either alone or in the presence of γc-cytokines. A potential difference between both compounds that could explain these results is the lack of ability of AC-484 to alter the levels of PTPN1 and PTPN2 compared with HODHBt.

## Discussion

In this work, we have characterized HODHBt as a PTPN1/PTPN2 inhibitor that directly interacts with PTPN1 and PTPN2, inducing changes in the thermal stability of both proteins in vitro and leading to enhanced phosphorylation and transcriptional activation of different STATs. Because of the relevance of our previous work showing that HODHBt enhances immune functions and latency reversal, we analyzed the functions of the recently developed and clinically relevant PTPN1/PTPN2 active site inhibitor AC-484. Our results demonstrate that, like HODHBt, AC-484 promotes STAT5 transcriptional activation, induces immune activation, and synergizes with IL-15 to reactivate latency in an in vitro primary cell model of latency, albeit with a different mechanism of action.

**Figure 6:**
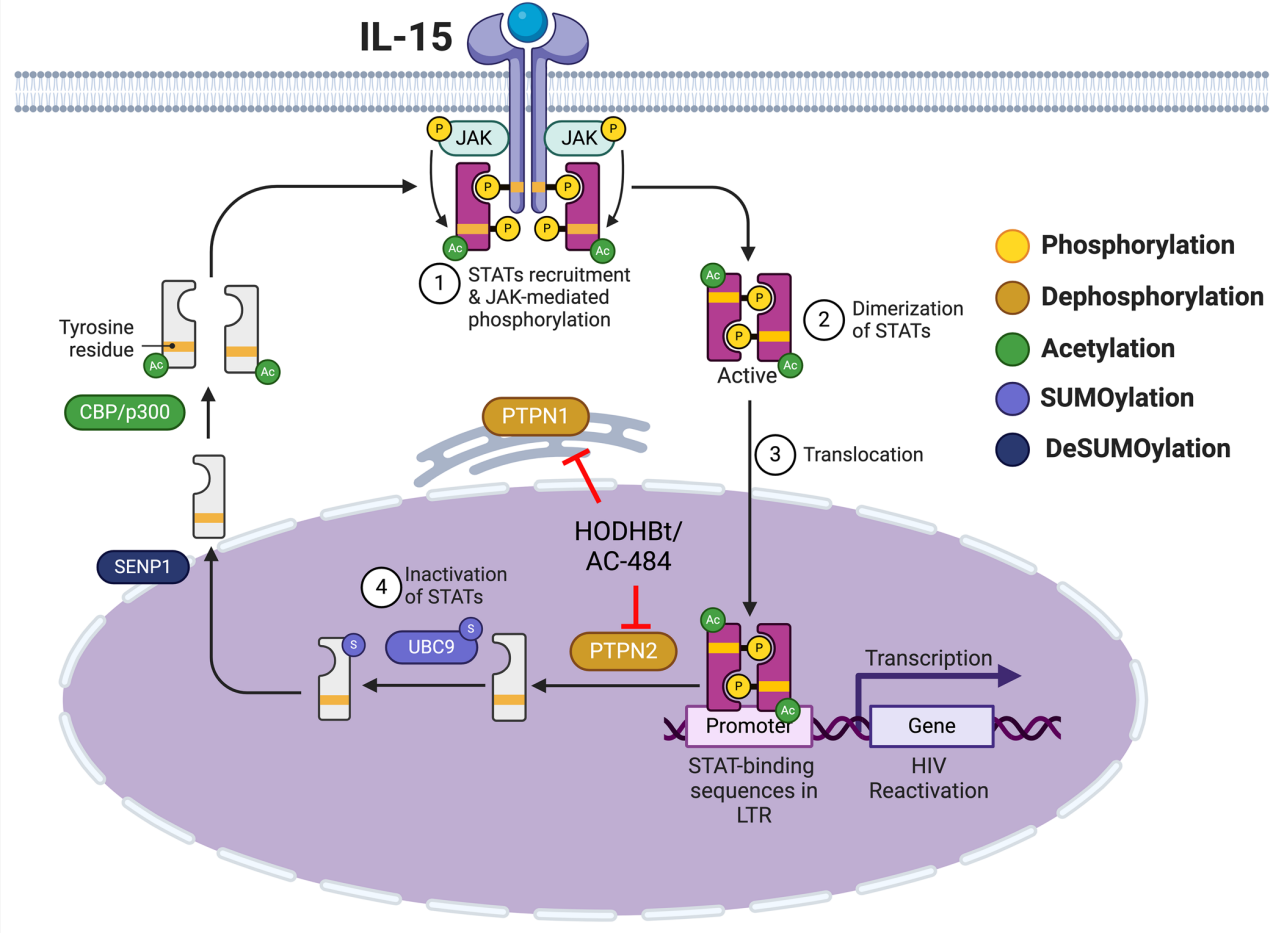
Proposed mechanism of action of HODHBt and AC-484. Created with Biorender.com.

In the context of HIV, we have previously shown that HODHBt increases cytokine-mediated HIV reactivation from latency due to enhanced STAT5 transcriptional activation and binding to the HIV LTR (4). In addition, we have demonstrated that HODHBt reactivates latent viruses in cells isolated from ART-suppressed PLWH (6) and can also enhance NK cell killing of HIV-infected cells through increased STAT activation upon IL-15 treatment (7). Our identification of PTPN1 and PTPN2 as the targets of HODHBt is important and relevant, given the growing body of literature highlighting both NTPs as attractive therapeutic targets for cancer (22, 23, 35–38) and metabolic diseases such as diabetes and obesity (39–42). We have previously shown that HODHBt enhanced STAT5 phosphorylation, and this led to a reduction on STAT5 SUMOylation and accumulation in the nucleus in primary CD4 T cells (4). At the time, we did not know the actual targets of HODHBt. Based on our current and past studies, we now proposed that HODHBt and AC-484 target PTPN1 and PTPN2, and we suggest that dephosphorylation is a step required for SUMOylation of STAT5 and translocation back into the cytoplasm (Figure 6) (4).

A recent study investigating a related compound of AC-484, Compound-182, exhibited promise in small animal models for cancer therapeutics by demonstrating that in vivo administration of Compound-182 led to augmented activation and recruitment of T cells in solid tumors, resulting in a reduction in tumor growth (43). Crucially, this was achieved without triggering the development of cytokine release syndrome or autoimmunity, suggesting that targeting PTPN1 and PTPN2 in vivo may not be associated with toxicities caused by immune system overactivation. Our work demonstrates that these targets can now be expanded to other infectious diseases and, in particular, to HIV.

The ability of AC-484 to enhance immune activation and STAT5 phosphorylation through inhibition of PTPN1 and PTPN2 (16) led us to hypothesize that AC-484 functions similarly to HODHBt and has the potential for use as a component of HIV cure strategies. Direct comparison of HODHBt and AC-484 on STAT5 transcriptional activity with and without IL-15 showed that AC-484 is a much more potent transcriptional activator in the HEK–Blue–IL-2/IL-15 cell line. However, we saw lower activity of AC-484 in reversing HIV latency. We speculate that the differences on transcriptional activation seen between HODHBt and AC-484 are cell type and/or gene dependent. The HEK–Blue–IL-2/IL-15 cell line has been optimized so that STAT5 binding is the only signal needed to induce transcriptional activation. The HIV LTR is a complex promoter subject to epigenetic regulation such as histone acetylation and histone methylation among others (44–47). Furthermore, in primary CD4 T cells, effective latency reversal must overcome several blocks, including blocks in elongation, splicing, nuclear export, and/or translation (48–53). We have demonstrated that 1 of the key effects of HODHBt is sustained γc-cytokine–stimulated STAT5 phosphorylation over time, which may facilitate increased HIV latency reversal (4). In primary total CD4 T cells, we observed that AC-484 failed to promote sustained STAT5 phosphorylation over time. We hypothesize that the inability of AC-484 to sustain STAT5 phosphorylation is why we did not see greater latency reversal in the primary cell model compared with HODHBt. Mechanistically, we observed that HODHBt led to a reduction on the levels of PTPN1 and PTPN2, while this was not observed with AC-484. Our previous studies with HODHBt did not reveal changes in the transcription of either phosphatase (4), suggesting that another mechanism such as proteasomal or lysosomal degradation may be involved in this process. Further studies will be warranted to elucidate the exact mechanism by which HODHBt reduces the protein levels of PTPN2 and to a lesser extent PTPN1. Despite these differences between HODHBt and AC-484, we confirmed that both compounds can synergize with IL-15 to reactivate latent HIV, are sufficient to induce immune activation of CD4 T, CD8 T, and NK cells, but AC-484 had activity at a 10-fold less concentration compared with HODHBt. Additionally, neither compounds induce production of proinflammatory cytokines in PBMCs, which is an important factor when developing new HIV LRAs.

Given our findings that AC-484 is sufficient to promote immune activation despite reduced latency reversal activity compared with HODHBt, future directions for this work will investigate the effects of AC-484 on the anti-HIV activity of immune effector cells including CD8 T cells and NK cells and its latency reversal properties in combination with other LRAs. Overall, our work highlights the therapeutic potential of PTPN1 and PTPN2 inhibition, leading to enhanced STAT activity, in the search for globally applicable and achievable HIV cure strategies.

## Methods

### Sex as a biological variable.

All experiments using primary human PBMCs use cells isolated from both male and female donors.

### CETSA-MS

CETSA-MS was performed at Pelago Biosciences, Sweden.

#### Sample matrix.

Pooled human PBMCs were purchased from 3H Biomedical. Cells where thawed the day before the experiment in RPMI 1640 medium supplemented with 10% FBS and 1% penicillin-streptomycin (PS) (all from Thermo Fisher Scientific) and cultured at 37°C and 5% CO_2_. For the experiment, the cells were pelleted, washed with HBSS (Thermo Fisher Scientific), and pelleted again. Cell viability was measured with trypan blue exclusion, and cells with a viability above 90% were used for the experiment.

K562 cells were obtained from ATCC (CCL-243). They were cultured at 37°C and 5% CO_2_ in RPMI 1640 medium with 10% FBS and 1% PS (all from Thermo Fisher Scientific). For the experiment, the cells were pelleted, washed with HBSS (Thermo Fisher Scientific), and pelleted again. Cell viability was measured with trypan blue exclusion, and cells with a viability above 90% were used for the experiment.

For both cell types, cell pellets were resuspended in CETSA buffer (20 mM HEPES, 138 mM NaCl, 1 mM MgCl_2_, 5 mM KCl, 2 mM CaCl_2_ [pH 7.4]) at a density of 40 × 10^6^ cells/mL and used as the 2× matrix solution.

#### Compounds.

HODHBt was purchased from Bio-techne (catalog 6994) and stored at –20°C.

#### Compressed CETSA-MS experiment.

PBMCs were divided into 8 aliquots each and mixed with an equal volume of either one of the 7 test compound concentrations or control at 2× final concentration in the experimental buffer. The resulting final concentrations of the compound were 1, 3, 10, 30, 100, 300, and 1,000 μM; 0.1% DMSO was used as a vehicle control. For K562, only the 1000 µM concentration of HODHBt and the 0.1% DMSO vehicle control were done in quadruplicates. Incubations were performed for 60 minutes at 37°C.

Each of the 8 treated samples (8 concentration points) was divided into 24 aliquots (12 temperature points, 2 replicates) that were all subjected to a heat challenge. After heating, all temperature points for each test condition were pooled to generate 8 × 2 individual (compressed) samples. In addition, nonheated samples were processed alongside the experiment in a single replicate and used to distinguish between changes in thermal stability and changes in protein abundance caused by the treatment.

Precipitated proteins were pelleted by centrifugation (30,000*g*), and supernatants constituting the soluble protein fraction were kept for further analysis.

#### Protein digestion and labeling.

Equal amounts of total protein from each soluble fraction were subjected to reduction and denaturation, followed by alkylation with chloroacetamide. Proteins were subsequently digested with endoproteinase Lys-C and trypsin.

After complete digestion had been confirmed by nano–liquid chromatography-tandem mass spectrometry (nanoLC-MS/MS), samples were labeled with 16-plex Tandem Mass Tag reagents (TMTPro, Thermo Fisher Scientific) according to the manufacturer protocol.

#### LC-MS/MS analysis.

For each TMT16-plex set, peptides were separated by multidimensional chromatography, and high-resolution MS/MS data was acquired with an Orbitrap Exploris 240 mass spectrometer coupled to a Dionex Ultimate3000 nanoLC system (both from Thermo Fisher Scientific).

#### Protein identification and quantification.

Protein identification was performed by database search against 95,607 human protein sequences in Uniprot (UP000005640, download date: 2019-02-21) using the Sequest HT algorithm as implemented in the ProteomeDiscoverer 2.5 software package. Data were recalibrated using the recalibration function in PD2.5, and the final search tolerance setting included a mass accuracy of 10 ppm and 50 mDa for precursor and fragment ions, respectively. A maximum of 2 missed cleavage sites was allowed using fully tryptic cleavage enzyme specificity (lysine [K], arginine [R], no proline [P]). Dynamic modifications included oxidation of methionine, and deamidation of asparagine and glutamine. Dynamic modification of protein N-termini by acetylation was also allowed. Carbamidomethylation of Cysteine, TMTpro modification of Lysine, and peptide N-termini were set as static modifications.

For protein identification, validation was done at the peptide-spectrum-match (PSM) level using the following acceptance criteria; 1 % FDR determined by Percolator scoring based on *q* value, rank 1 peptides only (54). 

For quantification, a maximum coisolation of 50% was allowed. Reporter ion integration was done at 20 ppm tolerance, and the integration result was verified by manual inspection to ensure the tolerance setting was applicable. For individual spectra, an average reporter ion signal/noise ratio of > 20 was required. Furthermore, shared peptide sequences were not used for quantification.

#### Compressed CETSA MS data processing and ranking.

Protein intensities were normalized, ensuring the same total ion current in each quantification channel. Intensity values were then log_2_ transformed and aligned between treatments and replicates, so each protein has the same mean intensity in all treatments and replicates.

The fold changes of any given protein across the concentration range was quantified by using the vehicle condition as the reference (i.e., a constant value of 1). Fold changes were also transformed to log_2_ values, to achieve a normal distribution around 0. Processed data were uploaded to Pelago data portal.

To estimate effect size (amplitude) and *P* value (significance) of the protein hits, the individual protein concentration-response curve was fitted using the following formula:


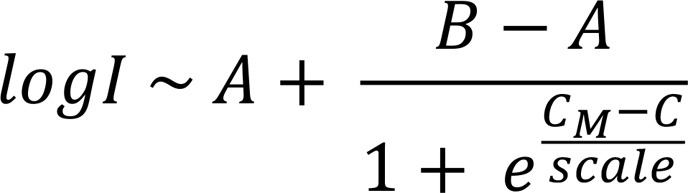


where *logI* indicates log_2_-transformed protein intensity; C indicates log_10_-transformed compound concentration; and A, B, *C*_M_, and *scale* indicate curve parameters for the fit. Using the values from the model fit, the effective concentration corresponding to 50% of maximal signal is estimated: *pEC*_M_* = −*C*_M_. Apparent *pEC*_M_* values should be used with caution, given the short incubation time and the specific experiment design.

Significance of the effect for each protein was assessed using 2-way ANOVA *F* test using the model fitted with formula above. Benjamini-Hochberg correction was applied to *F* test derived *P* values to adjust for multiple comparison.

### Reagents

Human recombinant IL-2 (rIL-2) and rIL-15 were obtained via the BRB/NCI Preclinical Repository. Human αIL-12 (catalog 500-P154G), αIL-4 (catalog 500-P24), TGF-β (catalog 100-21), rIL12 (catalog 200-12), rIL-4 (catalog 200-04), rIL-21 (catalog 200-21), rIL-7 (catalog 200-07), and rhIFN-β (catalog 300-02BC) were purchased from Peprotech. Human rIFN-α2 (catalog NBP2-34971) was purchased from Novus Biologicals. Human rIFN-γ (catalog 570206) was purchased from BioLegend. Raltegravir (catalog HRP-11680) and Nelfinavir (catalog ARP-4621) were purchased from the NIH HIV Reagent Program. Fluorogenic PTP1B catalytic domain assay kit (catalog 79764) and recombinant GST-tag TC-PTP (PTPN2, 30013) were purchased from BPSBioscience. CRISPR GFP-Cas9, PTPN1 and PTPN2 crRNA, and tracrRNA were purchased from IDT. AC-484 (catalog HY-145923) was purchased from MedChem Express. HEK-Blue CLR selection cocktail (catalog h-csm), Puromycin (catalog ant-pr-1), Blasticidin (catalog ant-bl-1), Zeocin (catalog ant-zn-1), and QUANTI-Blue solution (catalog rep-qbs2) purchased from Invivogen. CytoTox 96 nonradioactive cytotoxicity assay (catalog G1780) was purchased from Promega. CorPlex Cytokine panel kit was ordered from Quanterix (catalog 85-0329). Antibodies were purchased from BioLegend (PE aSTAT5 phospho [Y694], 936903; FITC αCD4, 300506; PerCPCy5.5 αCD56, 362506; APC-Cy7 αCD69, 310914), eBioscience (eF450 fixable viability dye), BD Horizon (BV786 αCD3, 563918; human FC block, 564220), Thermo Fisher Scientific (PE αCD8, 12-0086-42), Beckman Coulter (FITC KC57, 6604665), Cell Signaling Technologies (PTP1B, 5311S; TC-PTP [TC45], 58935S; CRKL, 38710S; STAT, 94205S; RPL7A, 2415S; pSTAT5 [Y694], 9359S; pSTAT1[Y701], 7649S; STAT1, 9177S; pSTAT3 [Y705], 9145S; STAT3, 9139S; pSTAT2, 4441P; STAT2, 4594S; pSTAT4, 4134S; STAT4, 2653S; pSTAT6, 9361S; STAT6, 9362S), Proteintech (PTPN2 polyclonal, 11214-1-AP), Sigma Aldrich (β-actin [AC-15], A5441), and Jackson ImmunoResearch (αRabbit secondary antibody, 111-035-046; αMouse secondary antibody, 115-0350146).

K562 cells were a gift from Katherine Chiappinelli (George Washington University, Washington, DC, USA).

#### Cell line culture.

K562 were cultured in complete RPMI.

#### CETSA.

For the CETSA experiments, the protocol was adapted from Jafari et al. (10). Naive CD4 T cells were isolated from donor PBMCs by negative selection and activated using αCD3/CD28 beads (Dynal/Invitrogen) for 72 hours as previously described (5). The cells were then expanded for a further 7 days in the presence of 30 IU/mL IL-2 in RPMI supplemented with 1% L-glutamine, 10% fetal bovine serum (FBS), and 1% PS (complete RPMI). On day 10, the cells were washed with PBS and resuspended in PBS supplemented with protease inhibitor cocktail (cOmplete, Roche) and phosphatase inhibitor cocktail (phosSTOP, Roche) at a concentration of 20 × 10^6^ cells/mL. Cell suspensions were lysed by freeze-thaw 3 times in liquid nitrogen, and the lysate fractions were separated from debris by centrifugation at 20,000*g* for 20 minutes at 4°C. Cell lysates were treated with 100 μM HODHBt or the inactive control HBt and incubated at 25°C for 30 minutes. Samples were then separated into 7 × 50 μL aliquots and heated at increasing temperatures for 3 minutes using a thermocycler (MultiGene OptiMAX). The samples were spun down at 15,000 rpm for 10 minutes to remove precipitates and analyzed by Western blot. The following antibodies were used at the noted concentrations: STAT5 (1:1,000), pSTAT5 (1:1,000), CRKL (1:1,000), PTPN1 (1:500), PTPN2 (1:1,000), and β-actin (1:5,000). Band intensities were quantified and plotted as a function of temperature to generate the melting curves of each protein/treatment combination.

For the purified protein CETSAs, PTPN1 catalytic domain was purified as described below. TC-PTP (PTPN2) catalytic domain protein was purchased from BPS Bioscience (catalog 30013). Protein was diluted to equal 62.5 ng in 100 μL PBS supplemented with protease and phosphatase inhibitors. The protein dilutions were treated with DMSO, 100 μM HODHBt, or the inactive control HBt and incubated at 25°C for 30 minutes. Samples were then split into 2 × 50 μL aliquots and heated at 55°C for 3 minutes using a thermocycler. The samples were spun down at 15,000 rpm for 10 minutes to remove precipitates and analyzed by Western blot. The following antibodies were used at the noted concentrations: PTPN1 (1:1,000) and PTPN2 (Proteintech, 1:1,000). Band intensities were quantified and plotted as a function of temperature compared with the unheated control.

#### PTPN1 protein purification.

PTPN1 protein was provided by Heidi Schubert and Chris Hill (University of Utah, Salt Lake City, Utah, USA). A total of 2 L of His-TEV-PTP1B (catalytic domain: 1-301; Addgene, 102719) in BL21(DE3)RIL (Agilent) cells was grown in Luria broth at 37°C until they reached an OD600 of 0.6 and then cooled, induced to a final concentration of 0.4 mM IPTG (Invitrogen) and grown overnight at 18°C. The cell pellet was resuspended in 80 mL of lysis buffer (20 mM Tris [pH 7.5], 40 mM imidazole, 300 mM NaCl, 10% glycerol) with protease inhibitors leupeptin (Roche), aprotinin, and pepstatin (both from GoldBio). The sample was sonicated prior to a high-speed spin to pellet the insoluble fraction. The soluble supernatant was incubated with 5 mL equilibrated Qiagen NiNTA resin for 30 seconds prior to washing the resin with an additional 100 mL of lysis buffer. The salt concentration was reduced to 100 mM prior to elution (20 mM Tris [pH 7.5], 250 mM imidazole, 100 mM NaCl, 10% glycerol). The protein was dialyzed against 50 mM Tris (pH 8.0). In total, 500 mM NaCl, 1 mM DTT, and homemade TEV protease were added overnight. A s200 SEC column (GE Healthcare) was run in 20 mM Tris (pH 7.5), 50 mM NaCl, and 1 mM DTT to finish the preparation. The protein was concentrated to 7–9 mg/mL and stored at –80°C.

#### PTPN1/PTPN2 catalytic domain inhibition assay.

HODHBt inhibition (IC_50_) of PTPN1 enzymatic activity was measured using the fluorogenic PTP1B (catalytic domain) assay kit (BPS Bioscience, 79764) following manufacturer’s instructions. It is designed to measure inhibition of enzyme catalyzation of dephosphorylation of fluorogenic substrate. For PTPN2, the catalytic domain of TC-PTP (PTPN2) was used instead of PTP1B (PTPN1).

The mode of inhibition of HODHBt was determined by adding a final concentration of 0.4 pg/mL of each enzyme to varying concentrations of PTP substrate and HODHBt. The fluorescence signal was measured every 15 seconds for 30 minutes by spectrophotometer using an excitation wavelength of 360 nm and an emission wavelength of 460 nm. Data were analyzed using GraphPad 9.0 software and Michaelis-Mention equation fit.

#### Genome editing via CRISPR/Cas9.

Predesigned guide RNAs for PTPN1 (5′-ACCACAACGGGCCCGTGCTC-3′) and PTPN2 (5′-GCACTACAGTGGATCACCGC-3′) were obtained from IDT. Protocol for transfection by electroporation with the Neon from IDT was followed. Briefly, ribonucleoproteins (RNPs) for PTPN1 and PTPN2 were prepared by first incubating 200 μM Alt-R-CRISPR-Cas9 crRNA (IDT) and Alt-R-CRISPR-Cas9 tracrRNA (IDT) (final duplex concentration of 44 μM) at 95°C for 5 minutes. The guide RNA duplexes were then combined with Alt-R Cas9 GFP (final concentrations 22 pmol and 18 pmol) and incubated at room temperature for 10–20 minutes as per the manufacturer’s instructions. The RNPs were then transfected into K562 cells (0.25 × 10^6^ per reaction) by electroporation. For the PTPN1 + PTPN2 dual condition, equal volumes of PTPN1 and PTPN2 RNP were transfected into the cells via electroporation (Neon). Forty-eight hours later, 0.5 × 10^6^ K562 cells were collected and stained for pSTAT5. The rest were collected for gDNA isolation and KO efficiency determination (described below).

#### KO efficiency analysis of CRISPR/Cas9-edited K562 cells.

Genomic DNA (gDNA) was obtained from cell pellets by resuspending in 50 μL Quick Extract DNA Extraction Solution (Lucigen, QE0905T) and following the extraction program as per the manufacturer’s instructions. Genomic DNA (2 μL) was then PCR amplified (50 μL total reaction volume) using the following primers: PTPN1 Forward (5′-CTATACCACATGGCCTGACTTT-3′), PTPN1 Reverse (5′-GAGTCTCAGGTACGCCTTTATTAG-3′), PTPN2 Forward (5′-ACTGCCAGTGGAAGCAAT-3′), PTPN2 Reverse (5′-TTTGGAGTCCCTGAATCACC-3′). KO efficiency was measured using the T7endonuclease I from NEB (catalog M0302S) and following manufacturer instructions. Analysis was performed using the GelAnalyzer 19.1 software and T7EI beta tool from Horizon Discovery.

#### Primary cell model of latency.

Naive CD4 T cells were isolated via negative selection from PBMCs obtained from HIV^–^ donors. Cultured Tcm cells were generated and infected as previously described (5, 55, 56). Naive CD4 T cells were isolated from PBMCs from HIV^–^ donors by negative selection (STEMCELL Technologies, 19555) and activated at 0.5 × 10^6^ cells/mL with αCD3/CD28 Dynabeads (1:1 bead/cell ratio) in the presence of 1 μg/mL αIL-4, 2 μg/mL αIL-12, and 10 ng/mL TGF-β for 72 hours. Dynabeads were removed on day 3, and cells were subsequently expanded in RPMI supplemented with 1% L-glutamine, 10% FBS, and 1% penicillin/streptomycin (complete RPMI) with 30 IU/mL IL-2 before being infected on day 7 via spinoculation with the X4-tropic virus NL4-3. Levels of intracellular p24 were assessed 72 hours later (day 10) by flow cytometry prior to the infected cells being crowded in 96-well round-bottom plates to facilitate spread of infection (100,000 cells/well). On day 13, the cells were uncrowded and plated in the presence of an ART cocktail (1 μM raltegravir + 0.5 μM nelfinavir) and 30 IU/mL IL-2, and p24 levels were again measured by flow cytometry. Ninety-six hours later (day 17), the CD4^+^ cells were sorted from the infected cultures by positive selection (Dynabeads CD4 Positive Isolation Kit, Thermo Fisher Scientific, 11331D), and p24 levels were measured before and after sort. The CD4^+^ cells were then resuspended at 1 × 10^6^ cells/mL and plated with reactivation conditions for a further 48 hours; reactivation was measured by p24 stain on day 19.

#### Flow cytometry.

Flow cytometry was used to measure the levels of STAT5 phosphorylation in the CRISPR/Cas9-edited K562 cells and total CD4 T cells, as well as immune activation in PBMCs. Between 0.3 × 10^6^ and 0.5 × 10^6^ cells for each condition were collected and washed with PBS before resuspension in 100 μL FACS buffer (PBS + 2% FBS) with 0.1 μL viability dye (eBioscience Fixable Viability Dye eFluor 450, Thermo Fisher Scientific, 65-0863-18). For immune activation flow cytometry, PBMCs were incubated with Fc block (564220, BD Biosciences) prior to staining with viability dye. Cells were incubated for 10 minutes at 4°C before being washed with 500 μL FACS buffer. Cells were then resuspended in 100 μL prewarmed Fix Buffer I (BD Bioscience, 557870) and incubated at 37°C for 10 minutes. Cells were washed with 500 μL FACS buffer, resuspended in precooled Perm Buffer III (BD Bioscience, 558050), and incubated on ice for 30 minutes. Cells were washed with 500 μL FACS buffer, resuspended with 100 μL FACS buffer + 2.5μL pSTAT5(Y694)-PE (BioLegend, 936903), and incubated for 1 hour at RT in the dark. Cells were washed with 500 μL FACS buffer and resuspended in 200 μL PBS/2% PFA and kept in the dark prior to analysis on a BD LSR Fortessa X20 flow cytometer with FACSDiva software (Becton Dickinson). Data were analyzed using FlowJo (TreeStar Inc.).

To analyze reactivated cells, cells were stained for CD4, viability, and intracellular p24-Gag as previously described (55).

#### SEAP and cytotoxicity assays.

HEK-Blue IL-2/15 cells, HEK-Blue IFN-α/β cells, and HEK-Blue IFN-γ cells were purchased from Invivogen. Cells were maintained in DMEM supplemented with 10% (v/v) heat-inactivated FBS, 1% penicillin, and 1% streptomycin (complete DMEM). Cells were selected with complete DMEM + 30 μg/mL blasticidin and 100 μg/mL zeocin. Cells were maintained in complete DMEM with 1× HEK-Blue CLR selection cocktail, and 1 μg/mL puromycin.

To evaluate the ability of HODHBt to enhance transcriptional activity of STAT1 and STAT2, HEK-Blue IFN-α/β cells and HEK-Blue IFN-γ cells were plated at 50,000 cells/well in a 96-well flat-bottom plate for 24 hours prior to treatment to facilitate adherence. Cells were treated in sextuplet for each HODHBt and IFN concentration for 24 hours. After 24 hours of treatment, plates were spun down at 15,000*g* for 5 minutes before 20 μL of each well was transferred to a fresh 96-well flat-bottom plate. Then, 180 μL of prepared fresh Quanti-Blue solution was added to each well, and plates were incubated at 37°C for 2 hours. Secreted embryonic alkaline phosphatase (SEAP) levels were measured using a spectrophotometer at 640 nm. For toxicity evaluation, 50 μL of each well was transferred to a fresh 96-well flat-bottom plate. Next, 50 μL of prepared CytoTox 96 reagent was added to each well, and the plates were incubated at room temperature in the dark for 30 minutes. Finally, 50 μL of stop solution was added to each well, and the absorbance was measured using a spectrophotometer at 490 nm.

To evaluate the transcriptional activity of AC-484 and HODHBt, HEK-Blue IL-2/15 cells were used following the same protocol as detailed above. Cells were treated in sextuplet for each compound and IL-15 concentration for 24 hours.

#### Western blotting.

K562 cells were treated with the indicated conditions for 24 hours. Primary total CD4 T cells were isolated from PBMCs by negative selection and treated with indicated conditions for 24 hours. Cells were then washed with PBS and lysed with NETN extract buffer comprised of 100 mM NaCl, 20 mM Tris-Cl (pH 8), 0.5 mM EDTA, 0.5% Nonidet P-40, protease inhibitor cocktail (cOmplete, Roche), and phosphatase inhibitor cocktail (phosSTOP, Roche) for 30 minutes on ice. Lysates were purified by centrifugation at 13,200*g* for 10 minutes at 4°C, and proteins were visualized on SDS-PAGE. All primary antibodies were used at 1:1,000 concentrations except for β-actin (1:10,000). Secondary anti-rabbit and anti-mouse antibodies were used at a 1:10,000 dilution.

#### Primary cell pSTAT2 assay.

Naive CD4 T cells were isolated from PBMCs from HIV^–^ donors by negative selection (STEMCELL Technologies, 19555) and activated at 0.5 × 10^6^ cells/mL with αCD3/CD28 Dynabeads (1:1 bead/cell ratio) in the presence of 1 μg/mL αIL-4, 2 μg/mL αIL-12, and 10 ng/mL for 72 hours. Cells were expanded in the presence of 30 IU/mL IL-2 for 48 additional hours before being treated with 100 μM HODHBt, 10 ng/mL IFN-α, or 100 μM HODHBt + 10 ng/mL IFN-α for 24 hours. pSTAT2 levels were measured by flow cytometry (Cell Signaling Technologies).

#### Primary cell pSTAT5 time course.

Total CD4 T cells were isolated via negative isolation from PBMCs from HIV^–^ donors. Cells were then pretreated with 100 μM HODHBt, 10 μM AC-484, and 5 μM AC-484 for 2 hours prior to the addition of 30 IU/mL IL-2. The 1-hour time point sample was taken and stained for pSTAT5 1 hour after the addition of IL-2. The 24-hour and 48-hour time points were stained at the respective time after IL-2 addition. For the IL-15 samples, cells were pretreated with 100 μM HODHBt or 10 μM AC-484 for 2 hours prior to the addition of 100 ng/mL IL-15. Samples were stained for pSTAT5 48 hours after the addition of IL-15, and flow cytometry analysis was performed as described above.

#### PBMC immune activation and cytokine analysis.

PBMCs from HIV^–^ donors were pretreated at 3 × 10^6^/mL for 2 hours with 100 μM HODHBt, 10 μM AC-484, 1 μM AC-484, and 500 nM AC-484; then IL-15 was added at 1 ng/mL and incubated for 48 hours. Cells were collected and stained for flow cytometry analysis, and supernatants were frozen at –20°C.

Frozen supernatants were thawed, and assay was performed according to manufacturer protocol. Ten cytokines were measured using Quanterix SP-X Corplex Cytokine Panel (IFN-γ, IL-1β, IL-4, IL-5, IL-6, IL-8, IL-10, IL-12P70, IL-22, TNF-α).

#### Statistics.

Statistical analyses were performed using GraphPad Prism 9.0 software. The statistical analysis used is indicated in each figure legend. *P* < 0.05 was considered statistically significant. Two-way ANOVA F test using Benjamini-Hochberg correction to derived *P* values to adjust for multiple comparisons were used, as well as Wilcoxon matched-pairs signed-rank test, Dunnett’s multiple-comparison test, and Tukey’s multiple-comparison test.

#### Study approval.

Volunteers 17 years and older at the Gulf Coast Regional Blood Center served as blood participants. WBC concentrates (buffy coat) prepared from a single unit of whole blood by centrifugation were purchased.

#### Data availability.

Values for data points shown in graphs are provided in the Supporting Data Values file. All additional data are provided in the supplement.

## Author contributions

AB and JNH conceptualized the work. JNH, TDZ, CL, MS, DCC, and AB designed methodology, and experiments were carried out by CSH, JT, JNH, TDZ, WW, DCC, MS, CL, ER, and EKM. AB acquired funding and performed project administration. AB, RBJ, and NSS supervised experimental output and manuscript preparation. JNH and AB wrote the original manuscript, and JNH, NSS, TDZ, and AB reviewed and edited the final manuscript.

## Supplementary Material

Supplemental data

Supplemental data set 1

Supplemental data set 2

Supplemental data set 3

Unedited blot and gel images

Supporting data values

## Version 1 08/08/2024

In-Press Preview

## Version 2 09/24/2024

Electronic publication
