# Supplementary material for: The HIV latency reversing agent HODHBt inhibits the phosphatases PTPN1 and PTPN2
Source: JCI Insight. 2024 Aug 8;9(18):e179680. doi: 10.1172/jci.insight.179680 (PMC11457865; doi:10.1172/jci.insight.179680)
Supplement: Supplemental data [file jciinsight-9-179680-s103.pdf]

Supplementary Materials for

The HIV latency reversing agent HODHBt inhibits the phosphatases PTPN1 and PTPN2

J. Natalie Howard, Thomas D. Zaikos, Callie Levinger, Esteban Rivera, Elyse K. McMahon, Carissa S. Holmberg, Joshua Terao, Marta Sanz, Dennis C. Copertino Jr., Weisheng Wang, Natalia Soriano-Sarabia, R. Brad Jones, Alberto Bosque

Corresponding author: abosque@gwu.edu

The PDF file includes:

Supplemental Figures 1-7

Other Supplementary Materials for this manuscript include the following:

Supplemental Data Files 1-3

Supplemental Figures

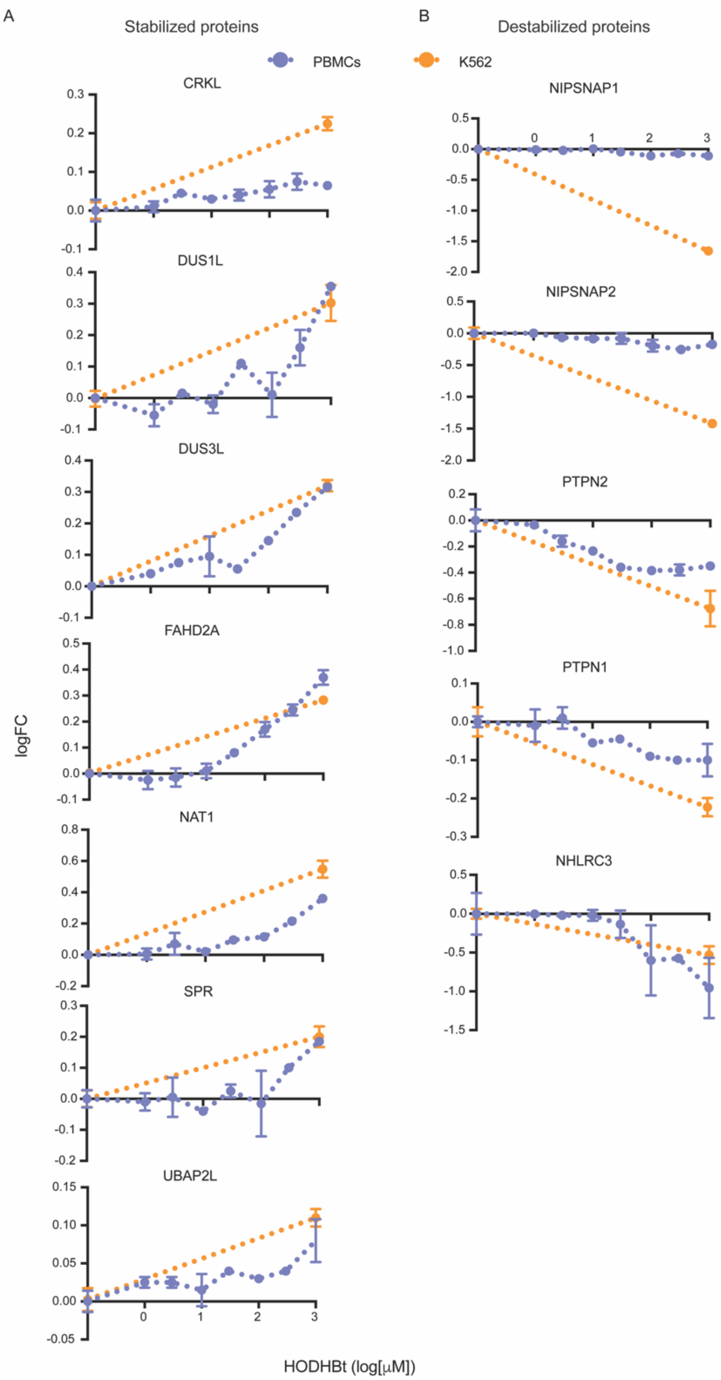

Supplemental Figure 1. Concentration-response curves for common proteins with changes in thermal stability for PBMCs and K562 cells in the TPP.

X-axis is $\log_{10}$-transformed compound concentration and y-axis is the log2FC of the peptide presence relative to the vehicle concentration calculated as indicated in the Materials and Methods. (**A**) Stabilized proteins. (**B**) Destabilized proteins.

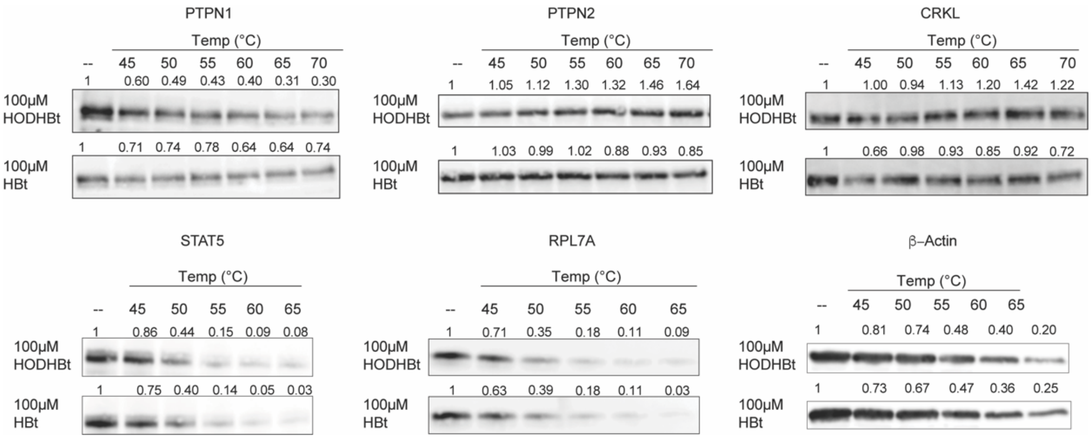

Supplemental Figure 2. Representative CETSA western blots.
Analysis of CETSA thermal shifts for proteins of interest in CD4 T cell lysates. Data representative of 2-4 western blots per protein.

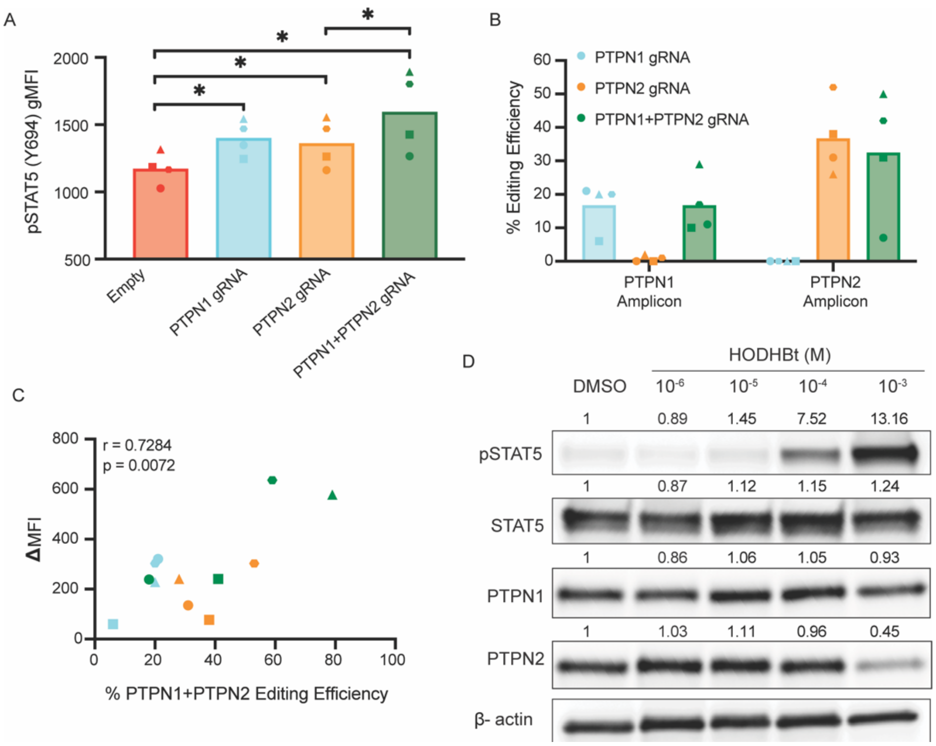

Supplemental Figure 3. PTPN1 and PTPN2 deletion increases STAT5 phosphorylation. (**A**) pSTAT5 levels in K562 cells after CRISPR/Cas9 knockout of PTPN1, PTPN2, or PTPN1/PTPN2 (n=4). Paired t-test used to calculate p-values (*p<0.05). (**B**) Editing efficiencies from the 4 independent experiments for each amplicon measured using T7 Endonuclease digestion. (**C**) Correlations between pSTAT5 ΔMFI (guide RNA minus Cas9 alone) for each experiment and combined knockout editing efficiency. Pearson correlation coefficients calculated. (**D**) Analysis of STAT phosphorylation and levels of PTPN1 and PTPN2 in K562 cells after HODHBt dose response treatment for 24 hours.

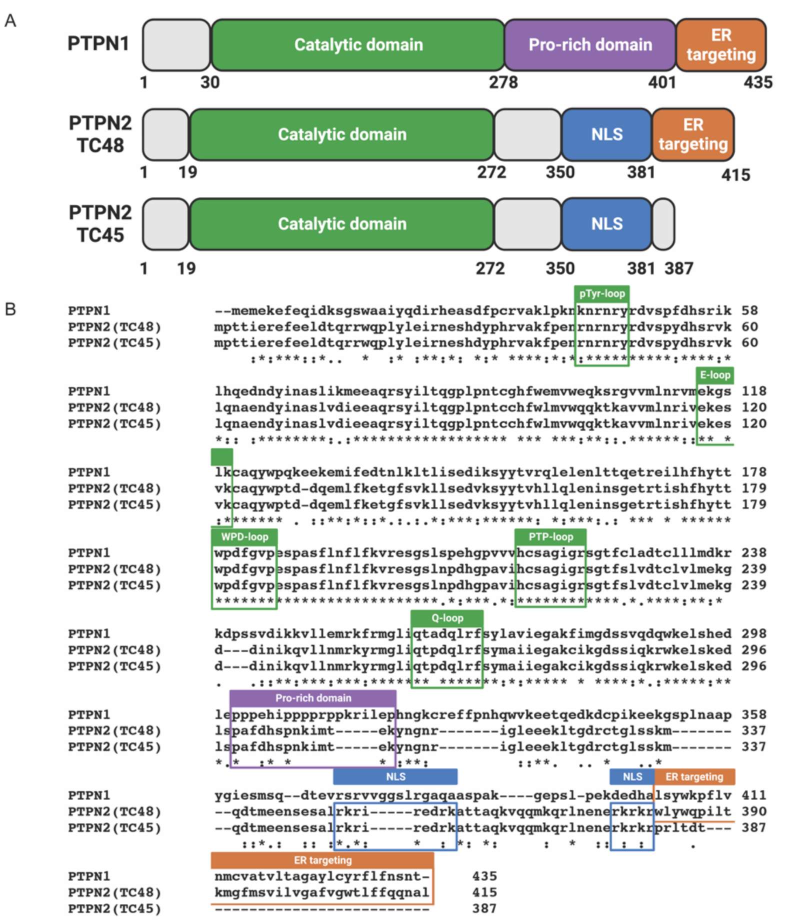

Supplemental Figure 4. Sequence similarity of PTPN1 and PTPN2.
(**A**) Schematic representation of PTPN1 and PTPN2. (**B**) Sequence alignment between PTPN1 and the two PTPN2 isoforms. The catalytic domains retain 98% similarity between proteins. Nuclear localization sequence (NLS) allows for nuclear translocation of PTPN2. Created with Biorender.com

PTPN1 (1-321)

[Inhibitor]	Vmax	Km
DMSO	233.5	4.52
250μM HODHBt	189.3	7.34
500μM HODHBt	151.5	7.78
1mM HODHBt	135	8.31

PTPN2 (2-315)

[Inhibitor]	Vmax	Km
DMSO	1.08	11.8
250μM HODHBt	0.76	12.89
500μM HODHBt	0.71	12.49
1mM HODHBt	0.42	9.25

Supplemental Figure 5. Enzymatic kinetic values for HODHBt against PTPNs.
Enzyme kinetic values for HODHBt against PTPN1 catalytic domain and PTPN2 catalytic domain. Calculated using Michaelis-Menten equation analysis in Prism 9 for Mac OS X software (GraphPad Software, Inc., La Jolla, CA)

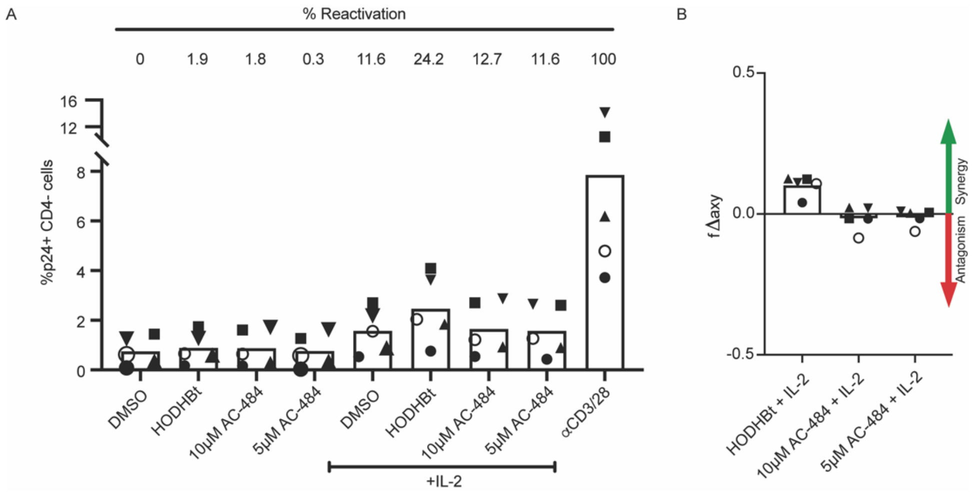

Supplemental Figure 6. AC-484 has limited reactivation activity with IL-2.
(**A**) Reactivation of latent HIV in T_{CM} measured by flow cytometry after treatment with 100µM HODHBt or 10µM AC-484 +/- 30 IU/mL IL-2, or αCD3/CD28 (n=5). (**B**) Bliss independence synergy calculations for reactivation.

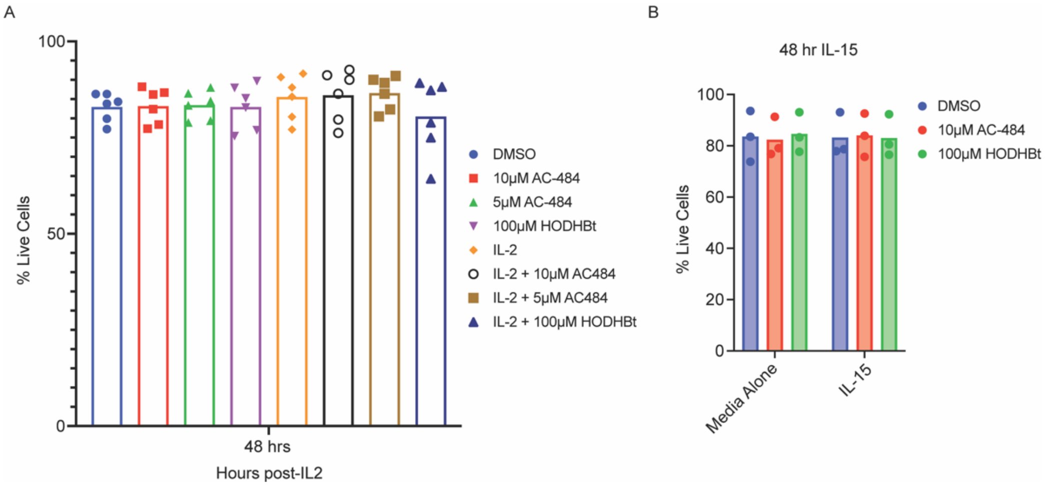

Supplemental Figure 7. Treatment with HODHBt and AC-484 has no effect on viability of primary CD4 T cells.
Viability of total CD4 T cells after pre-treatment for 2 hours with HODHBt and AC-484, and 48 hours in culture with and without IL-2 (n=6) (**A**) and IL-15 (n=3) (**B**).

Data S1. Raw data from Pelago CETSA-MS analysis of HODHBt
Intact PBMC and K562 data sets after treatment with HODHBt and subsequent analysis by LC-MS.

Data S2. Common proteins between PBMC and K562 data sets
Filtered for significance (p>0.01). Stabilized and destabilized proteins for each data set and common proteins between the two.

Data S3. STRING interaction analysis of common proteins
Interactions between common proteins from CETSA-MS analysis and STAT5.
