# Supplementary material for: The HIV latency reversing agent HODHBt inhibits the phosphatases PTPN1 and PTPN2
Source: JCI Insight. 2024 Aug 8;9(18):e179680. doi: 10.1172/jci.insight.179680 (PMC11457865; doi:10.1172/jci.insight.179680)

Full unedited blot for Figure 1C
PTPN1

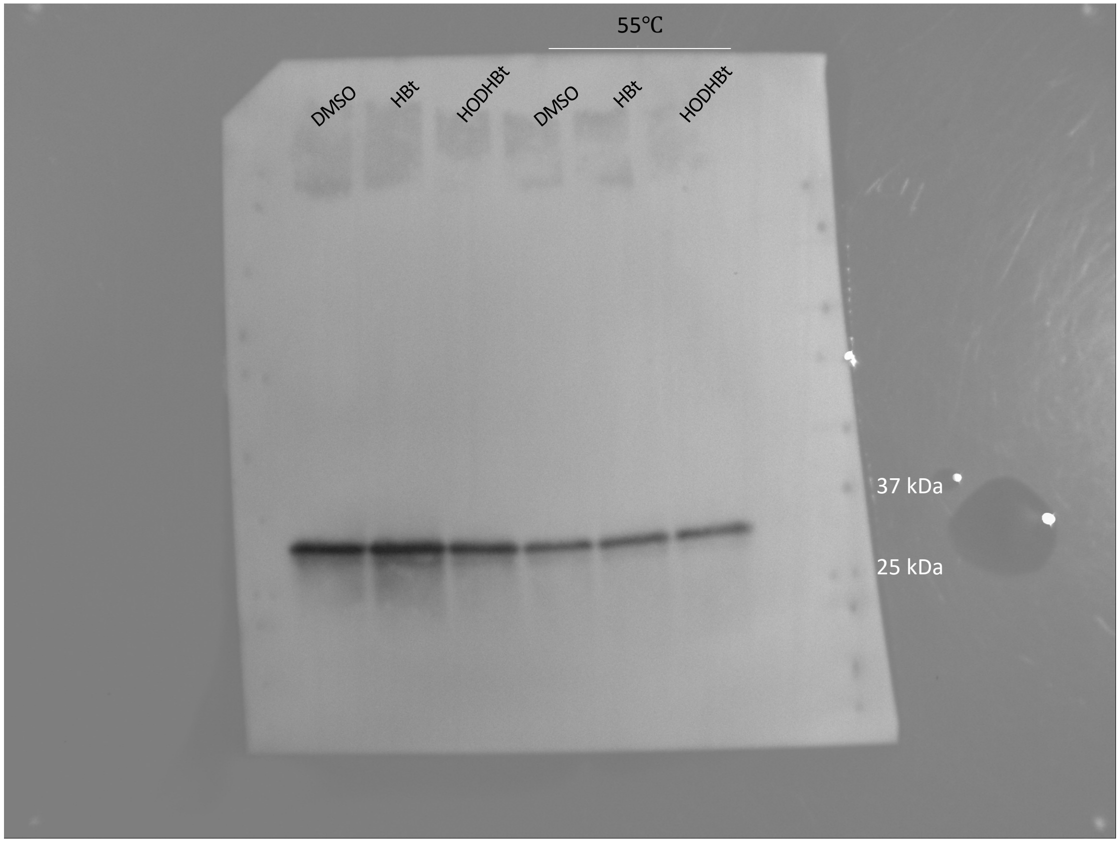

Full unedited blot for Figure 1C
PTPN2

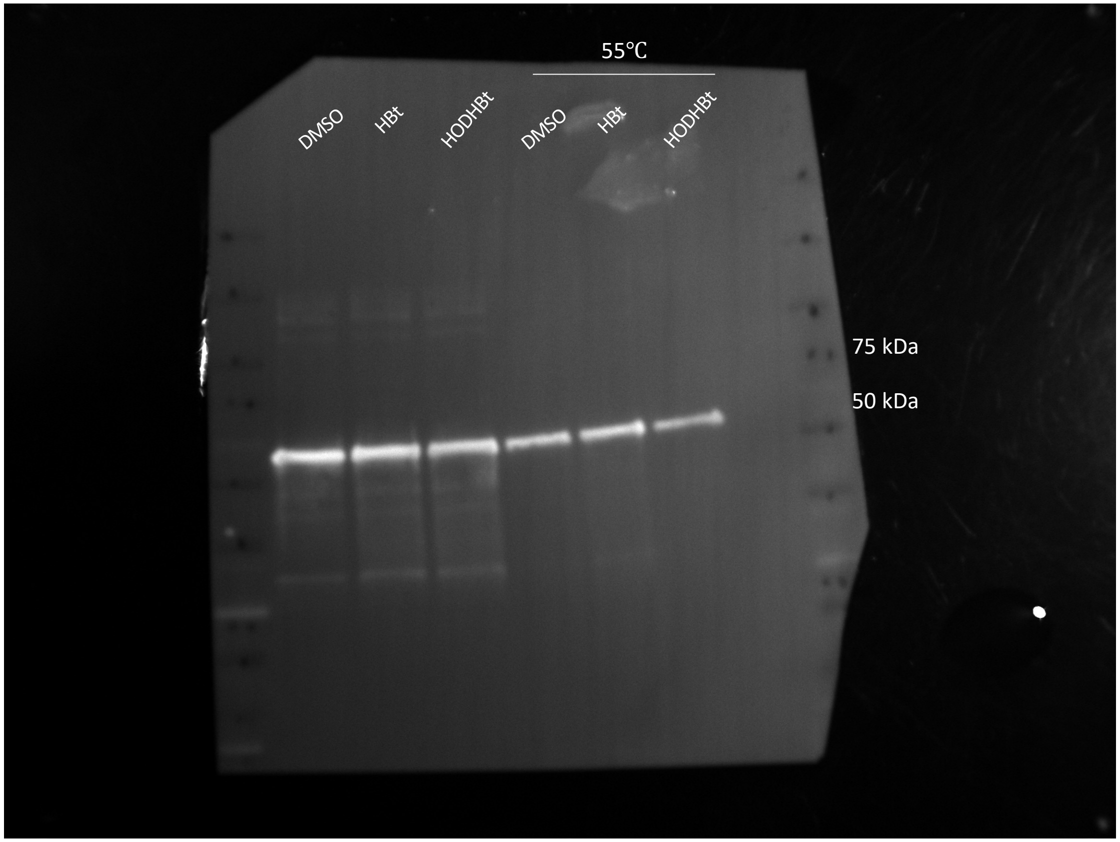

Full unedited blot for Figure 3A
pSTAT1

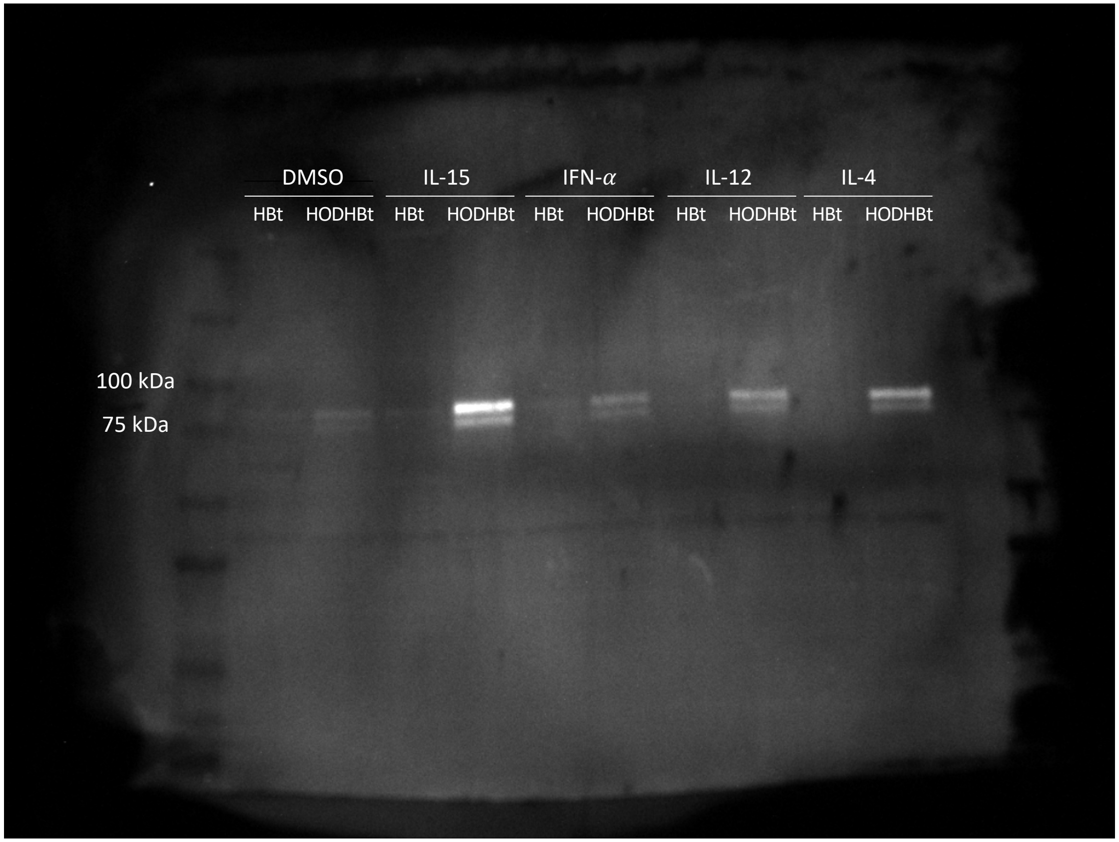

Full unedited blot for Figure 3A
pSTAT1 actin

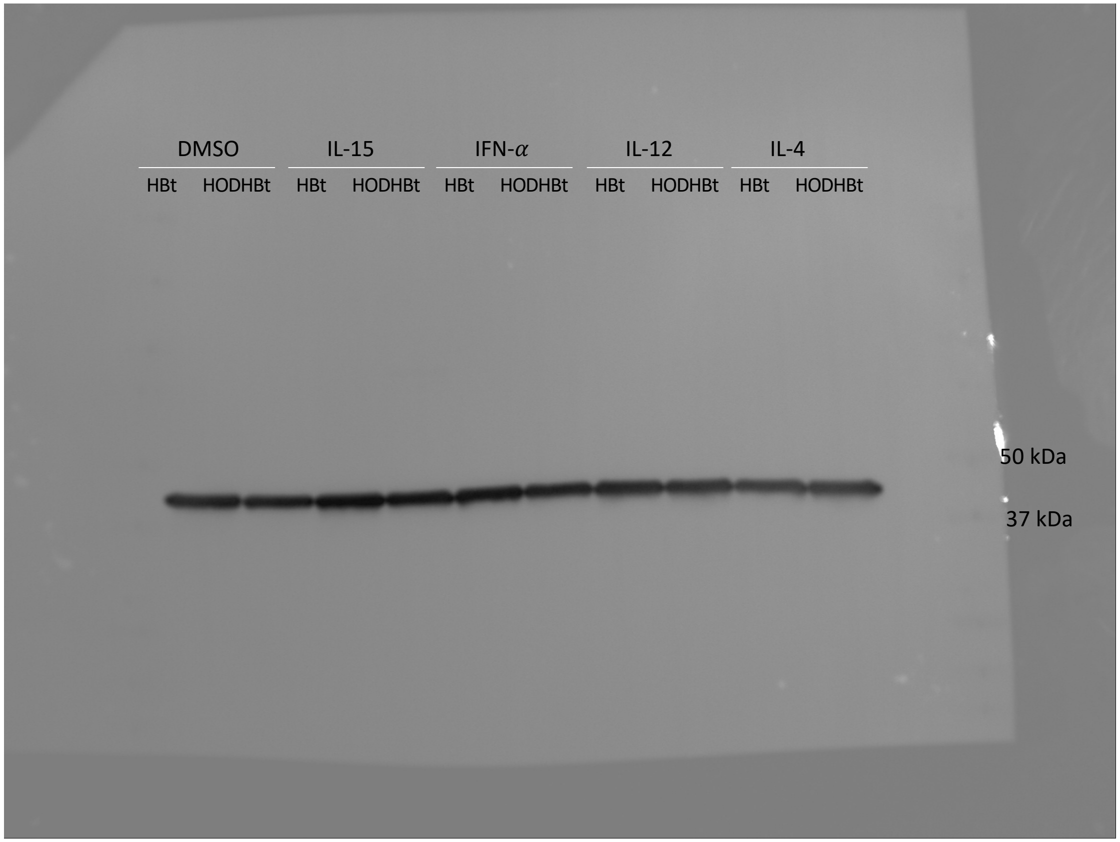

Full unedited blot for Figure 3A
STAT1

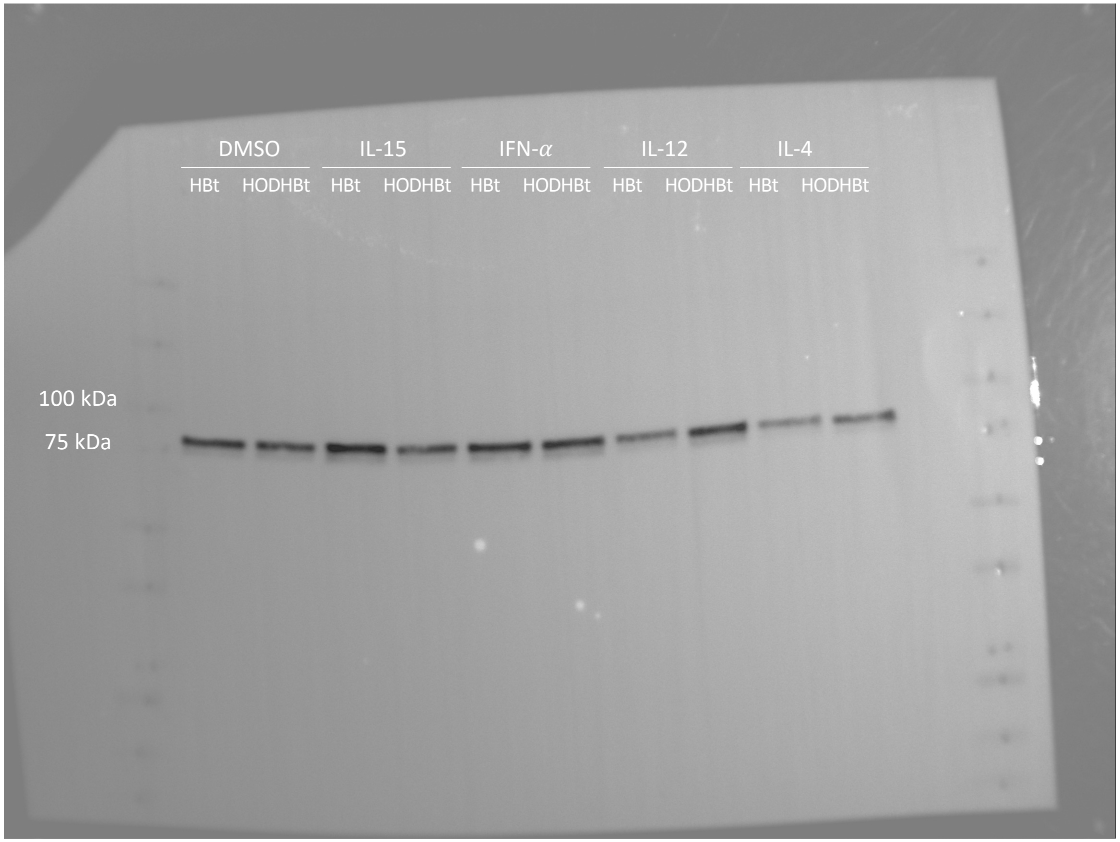

Full unedited blot for Figure 3A
STAT1 actin

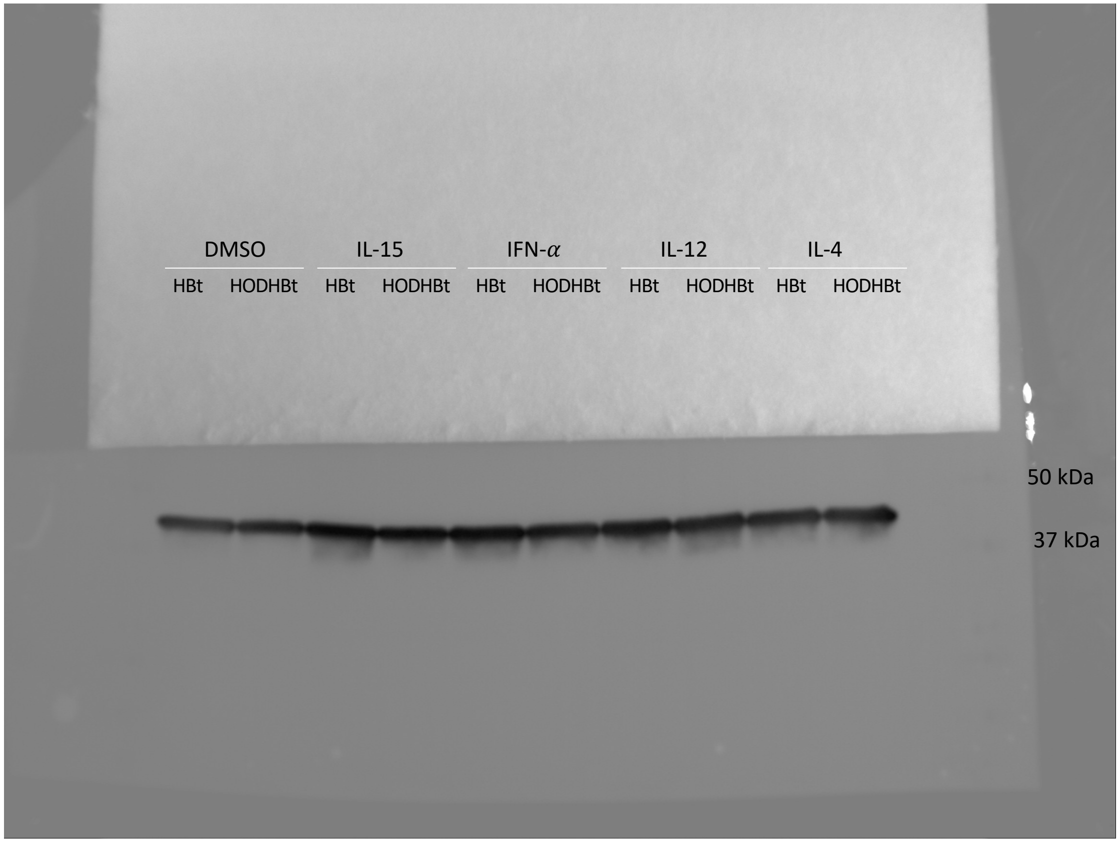

Full unedited blot for Figure 3A
pSTAT3

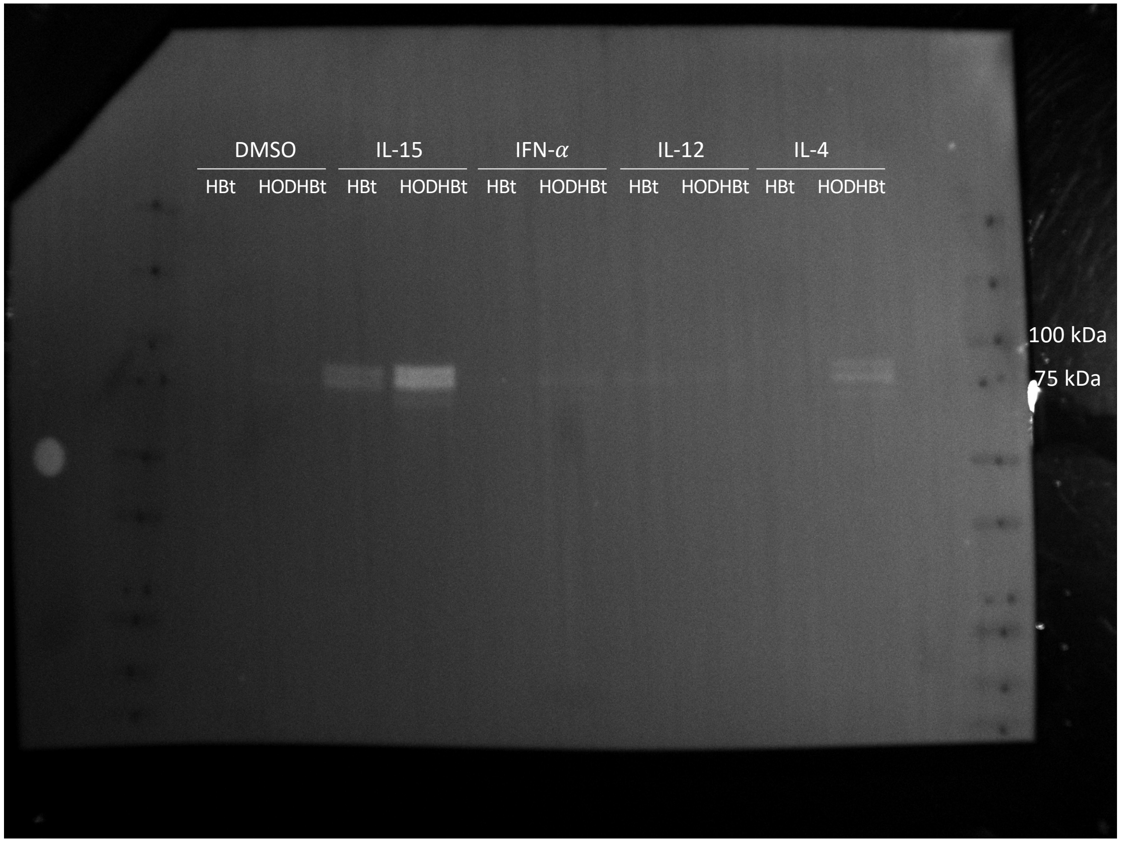

Full unedited blot for Figure 3A
pSTAT3 actin

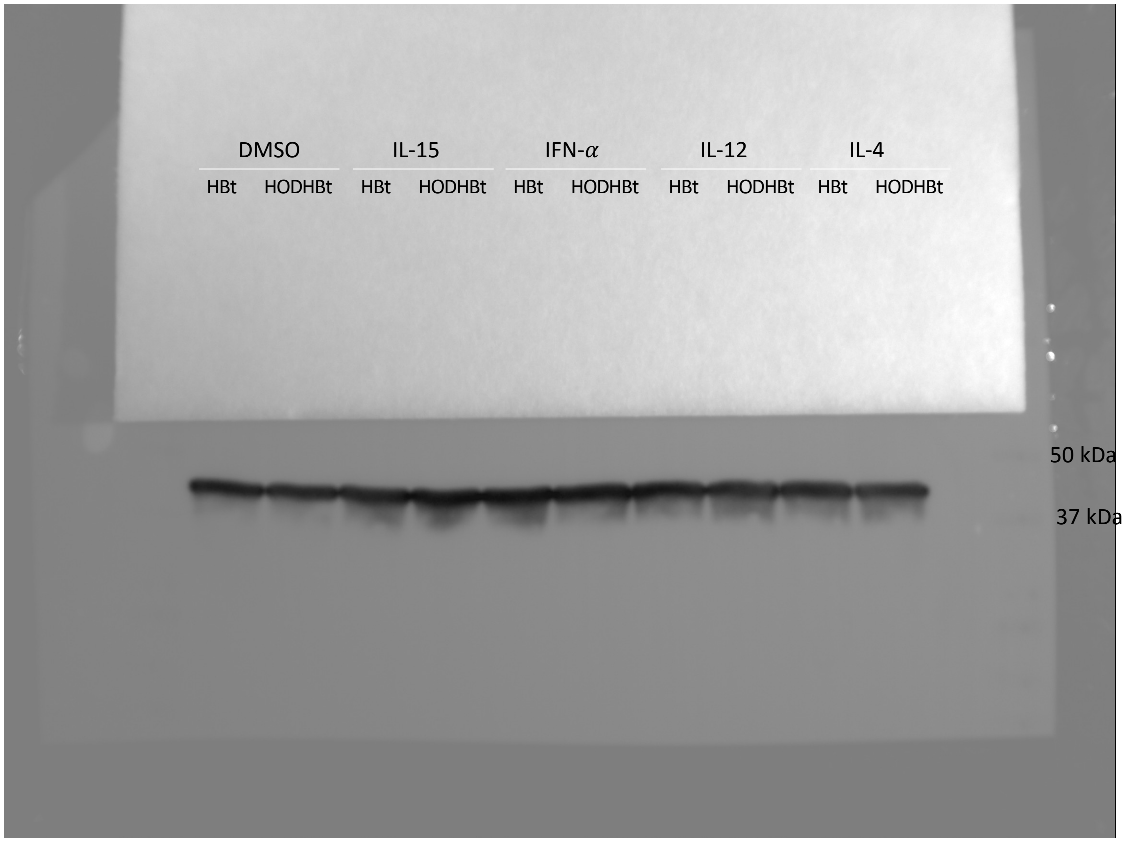

Full unedited blot for Figure 3A
STAT3

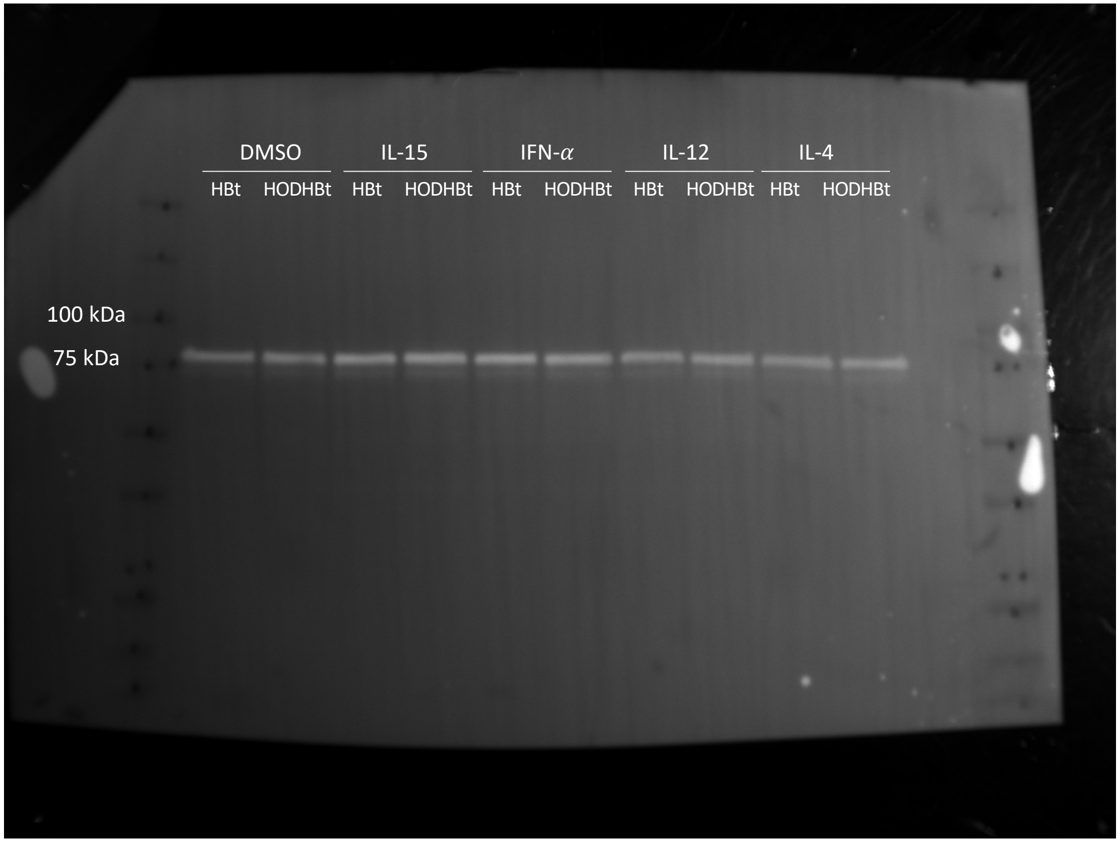

Full unedited blot for Figure 3A
STAT3 actin

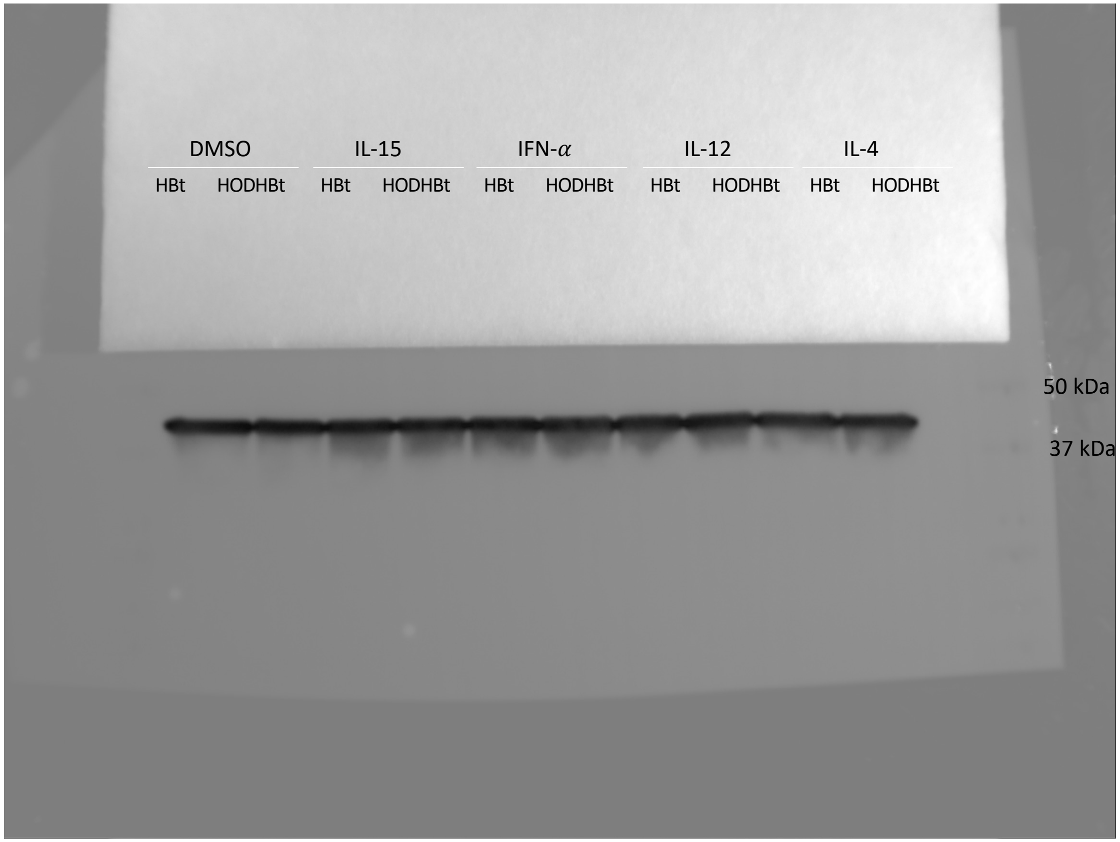

Full unedited blot for Figure 3A
pSTAT5

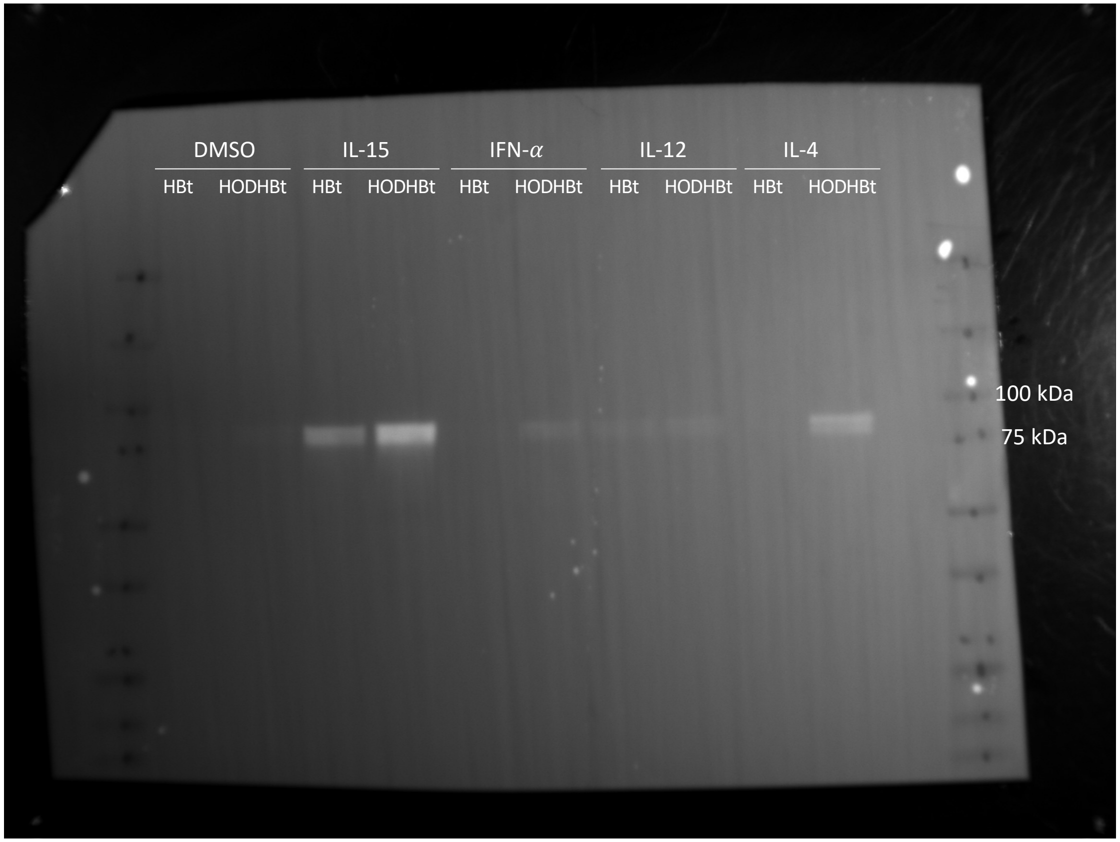

Full unedited blot for Figure 3A
pSTAT5 actin

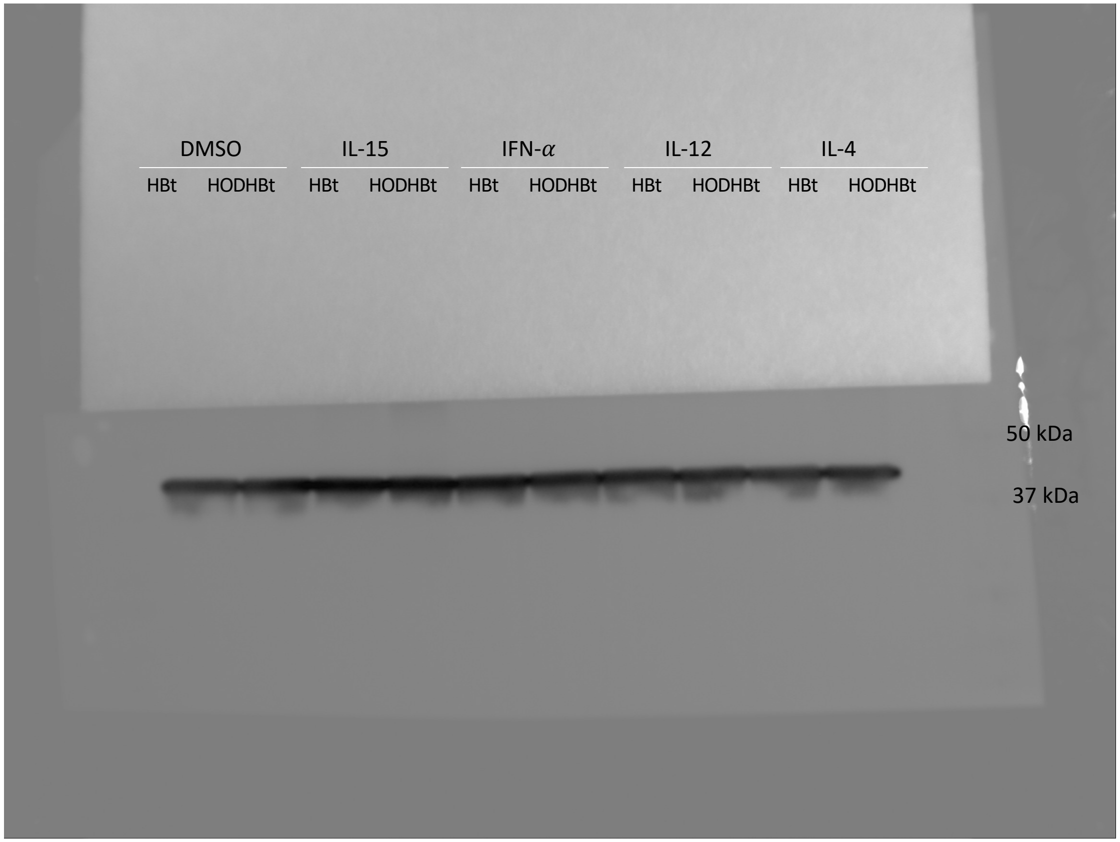

Full unedited blot for Figure 3A
STAT5

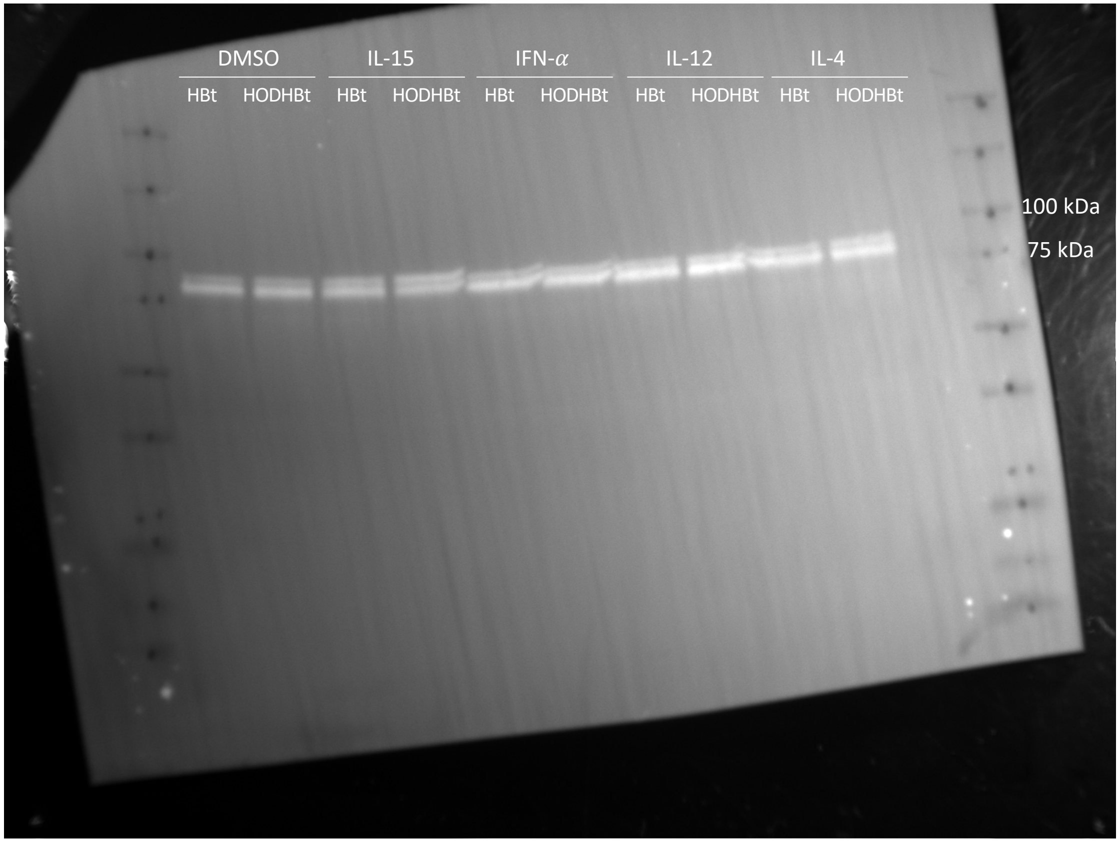

Full unedited blot for Figure 3A
STAT5 actin

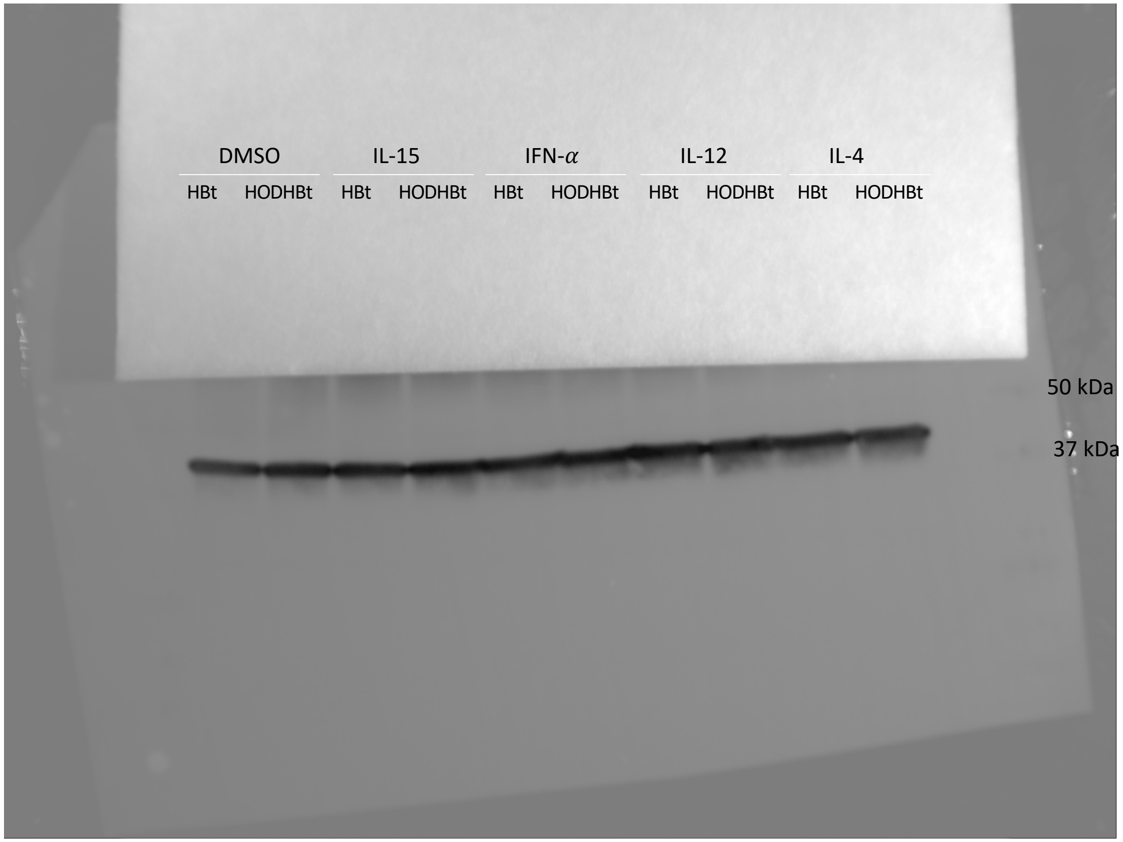

Full unedited blot for Figure 3B
pSTAT2

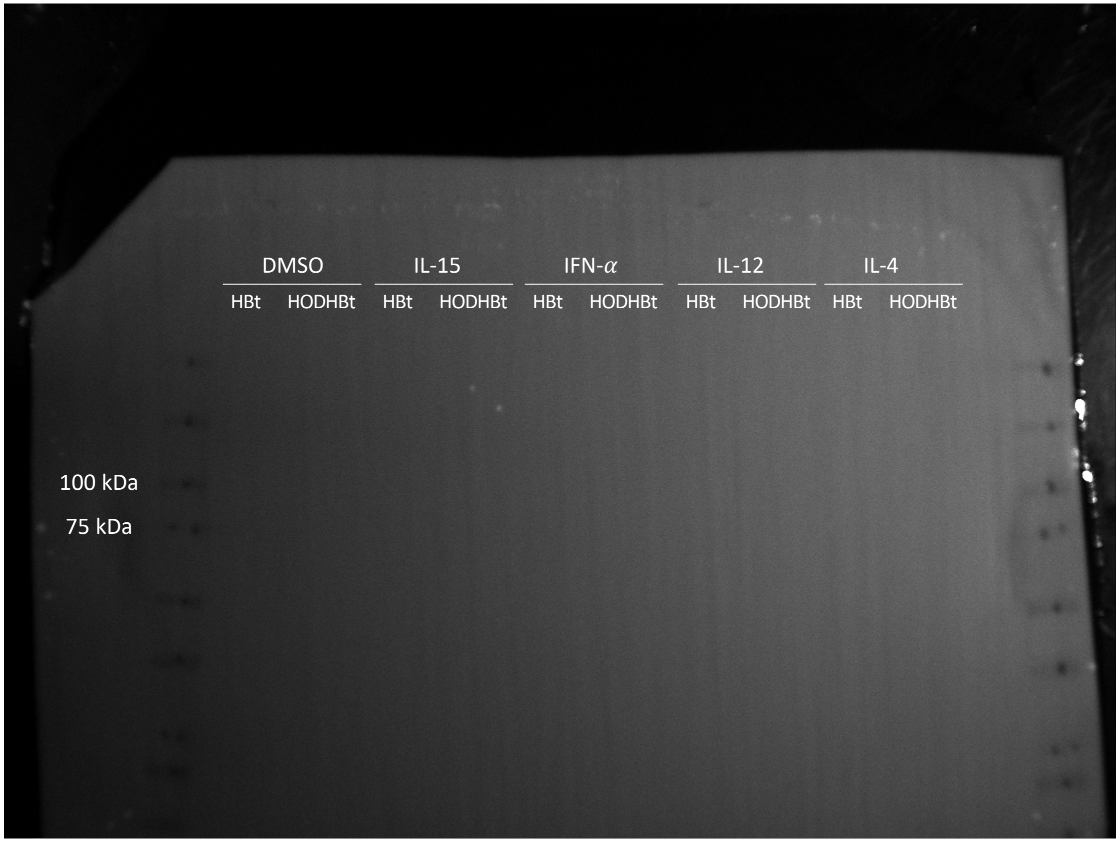

Full unedited blot for Figure 3B
pSTAT2 actin

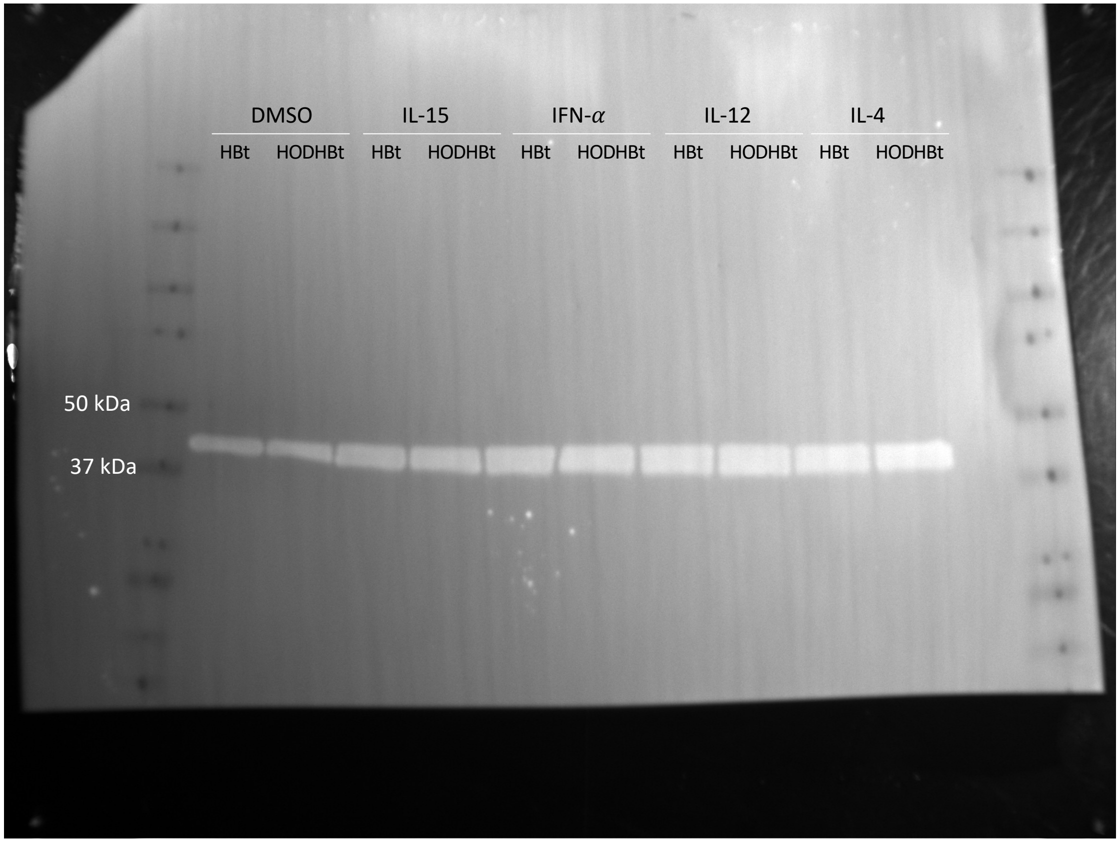

Full unedited blot for Figure 3B
STAT2

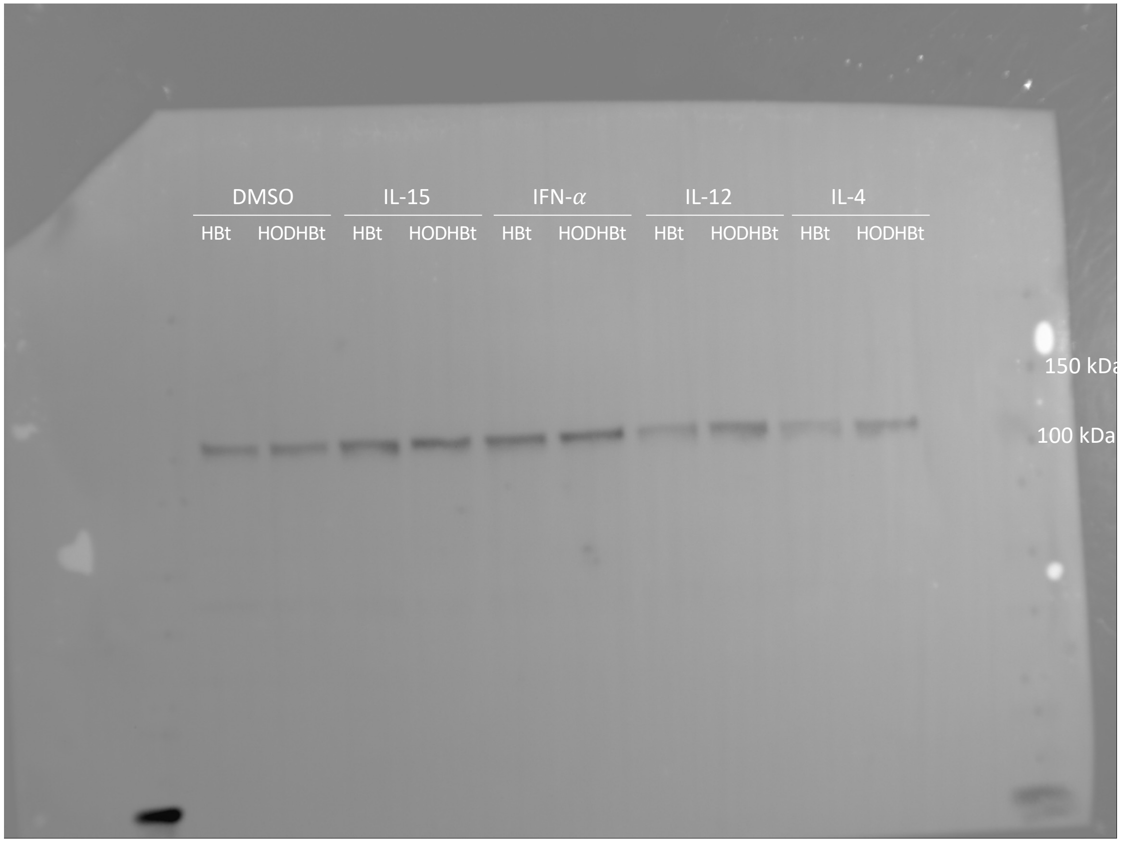

Full unedited blot for Figure 3B
STAT2 actin

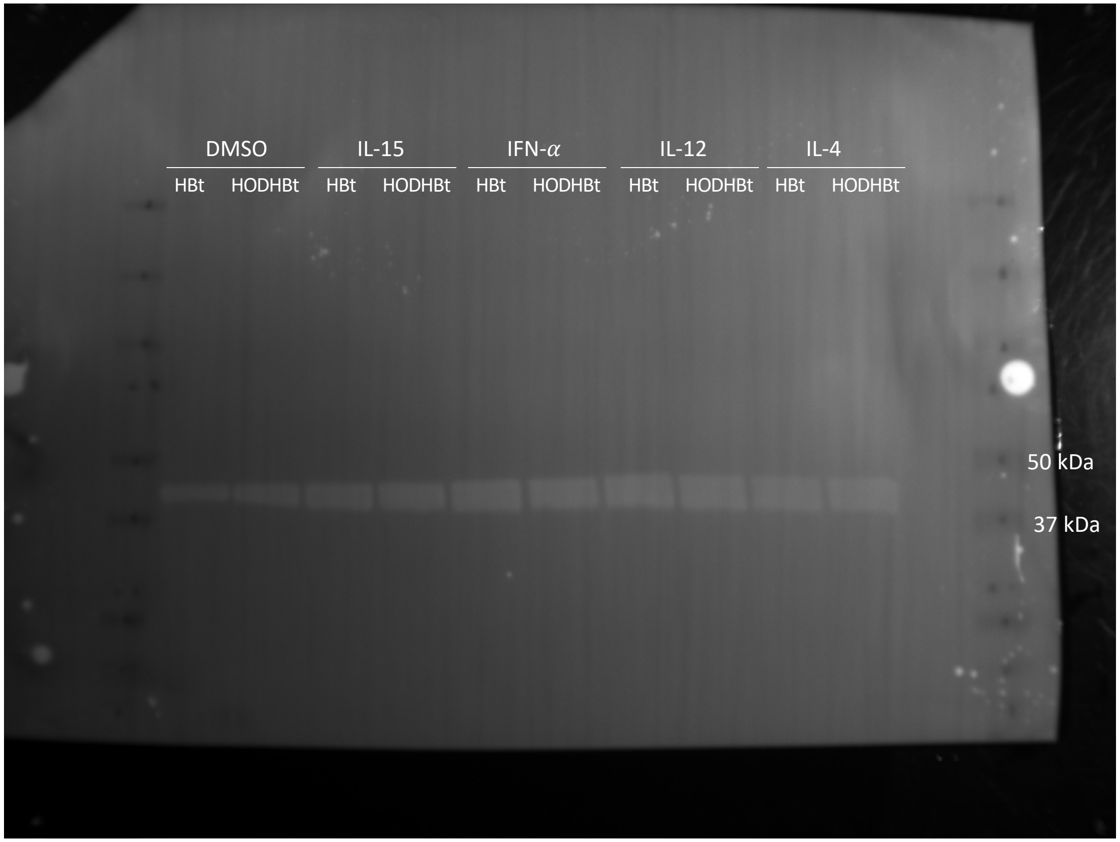

Full unedited blot for Figure 3B
pSTAT4

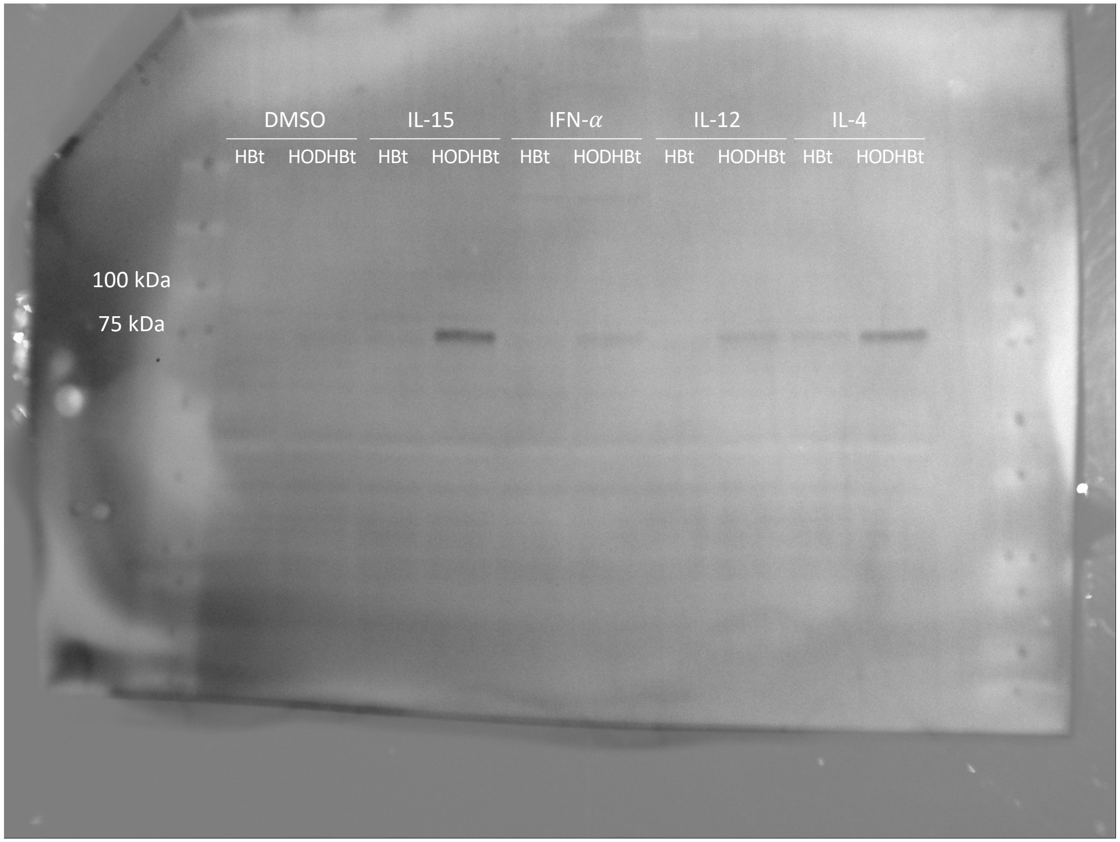

Full unedited blot for Figure 3B
pSTAT4 actin

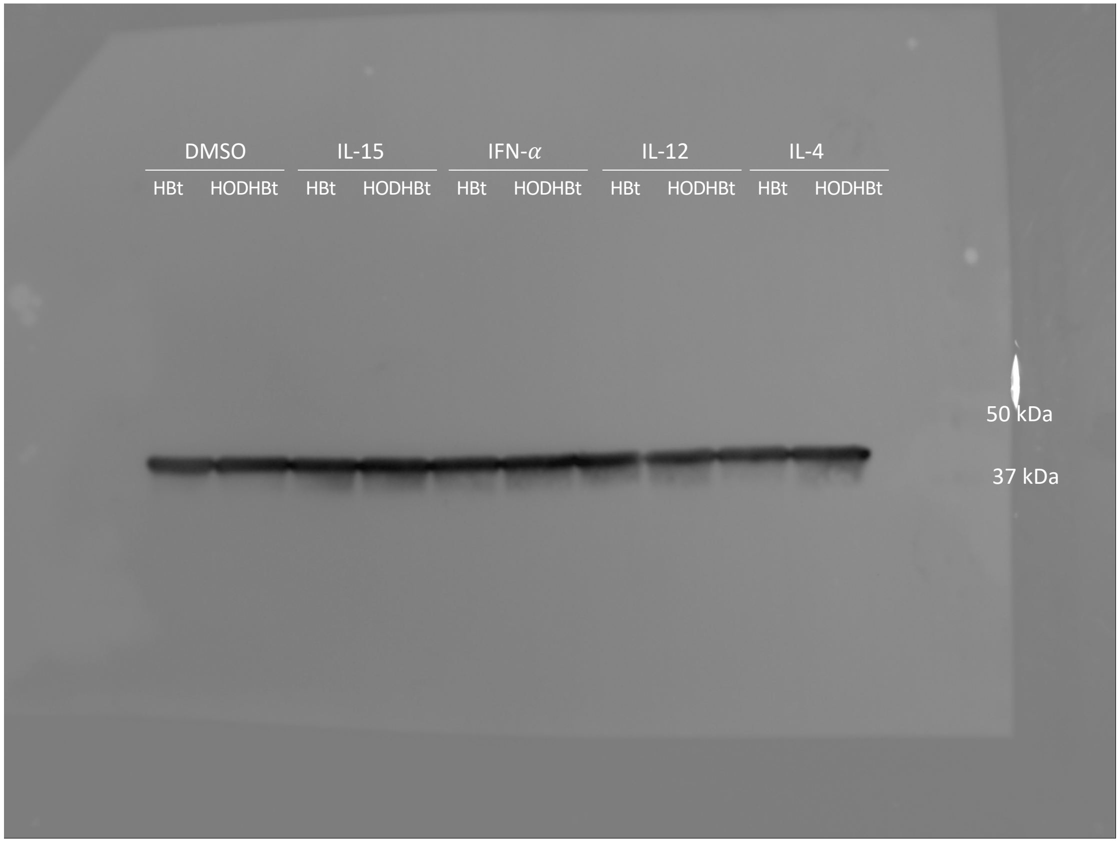

Full unedited blot for Figure 3B
STAT4

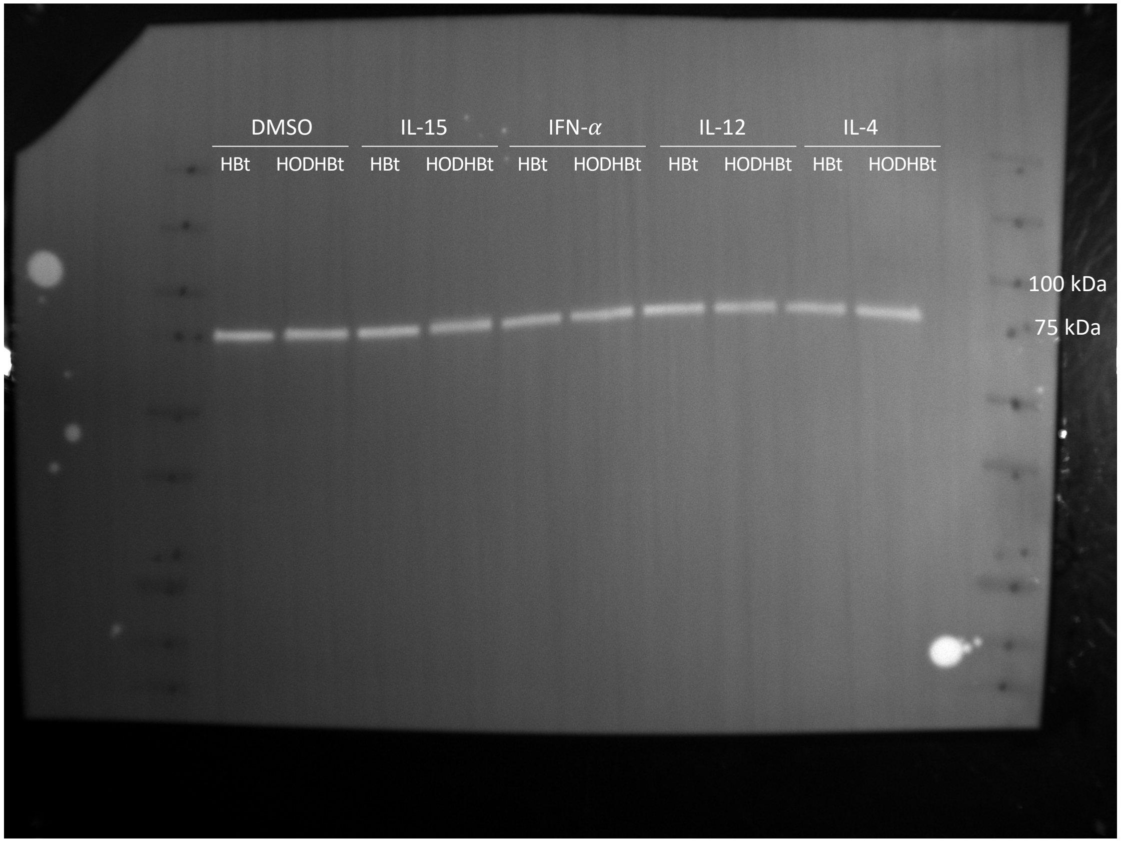

Full unedited blot for Figure 3B
STAT4 actin

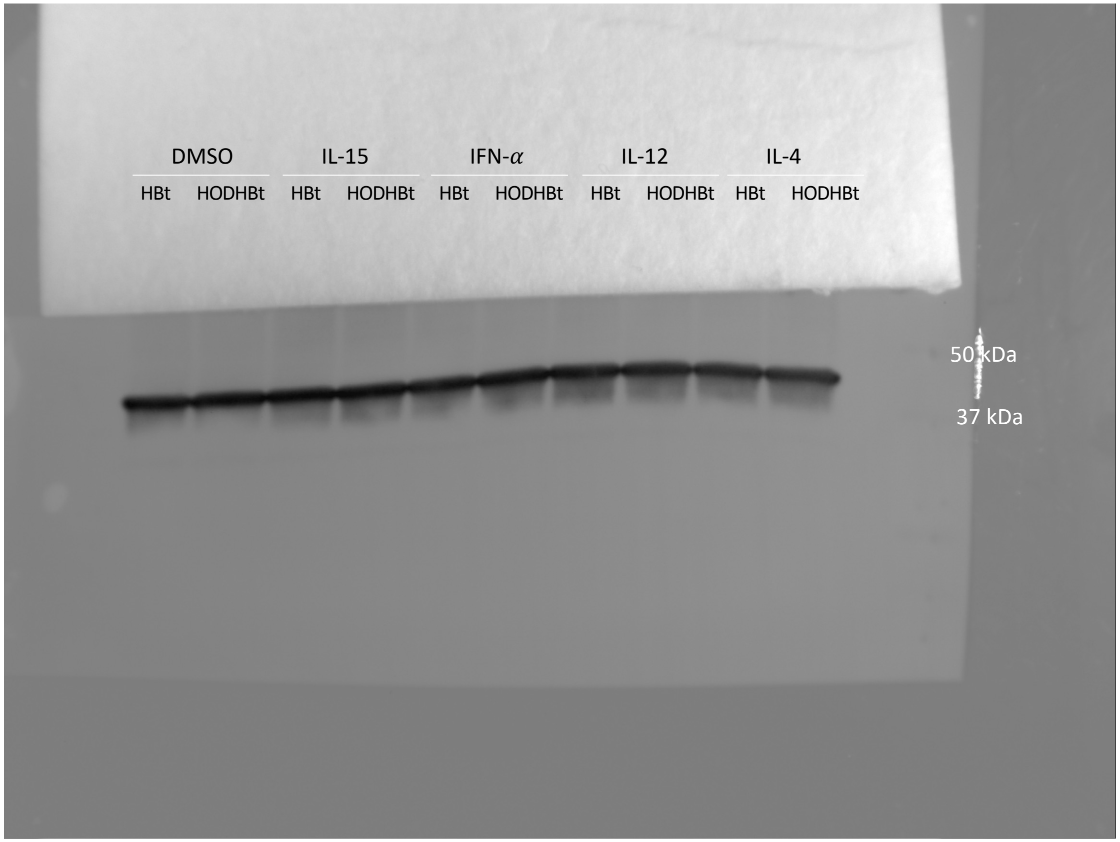

Full unedited blot for Figure 3B
pSTAT6

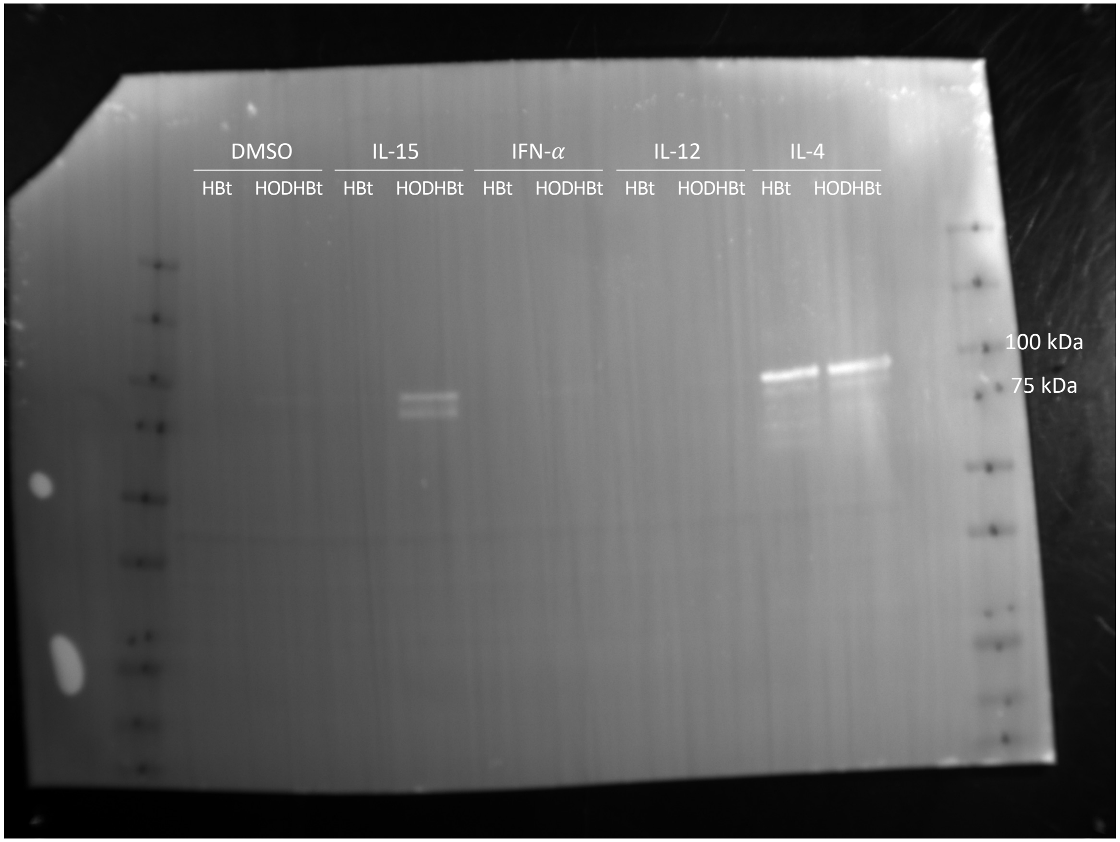

Full unedited blot for Figure 3B
pSTAT6 actin

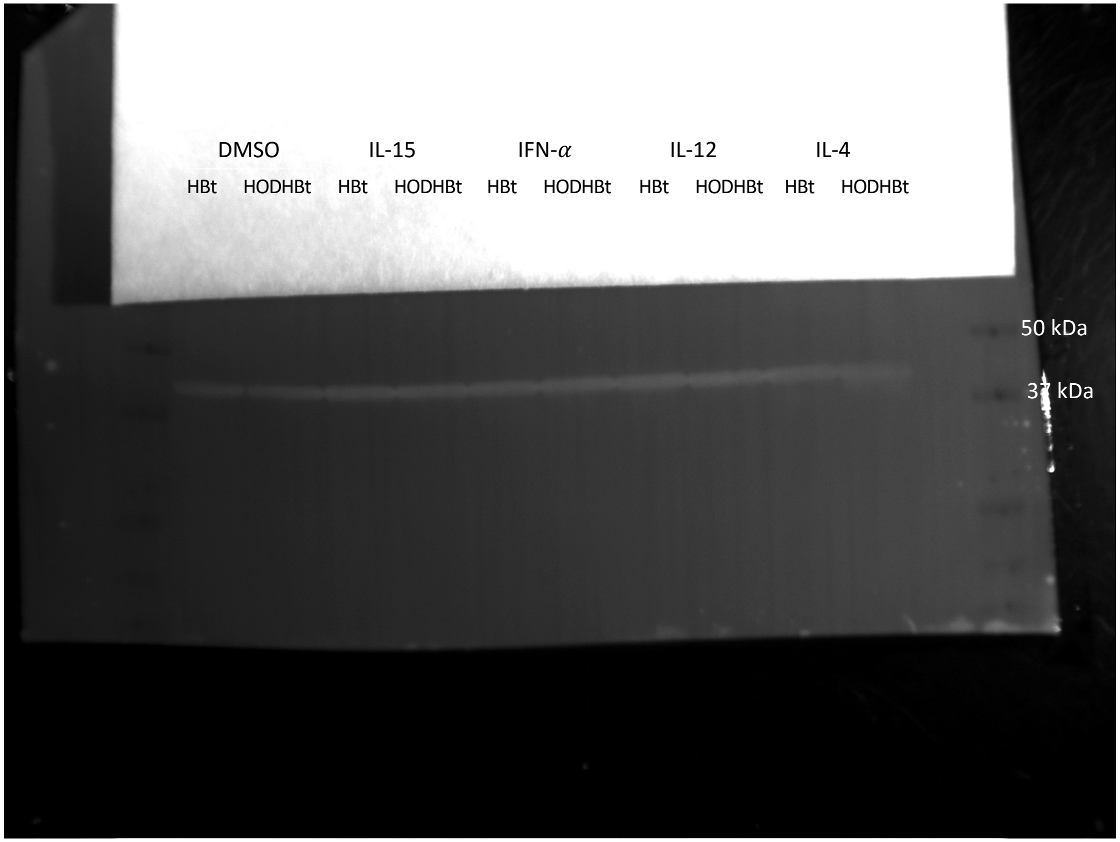

Full unedited blot for Figure 3B
STAT6

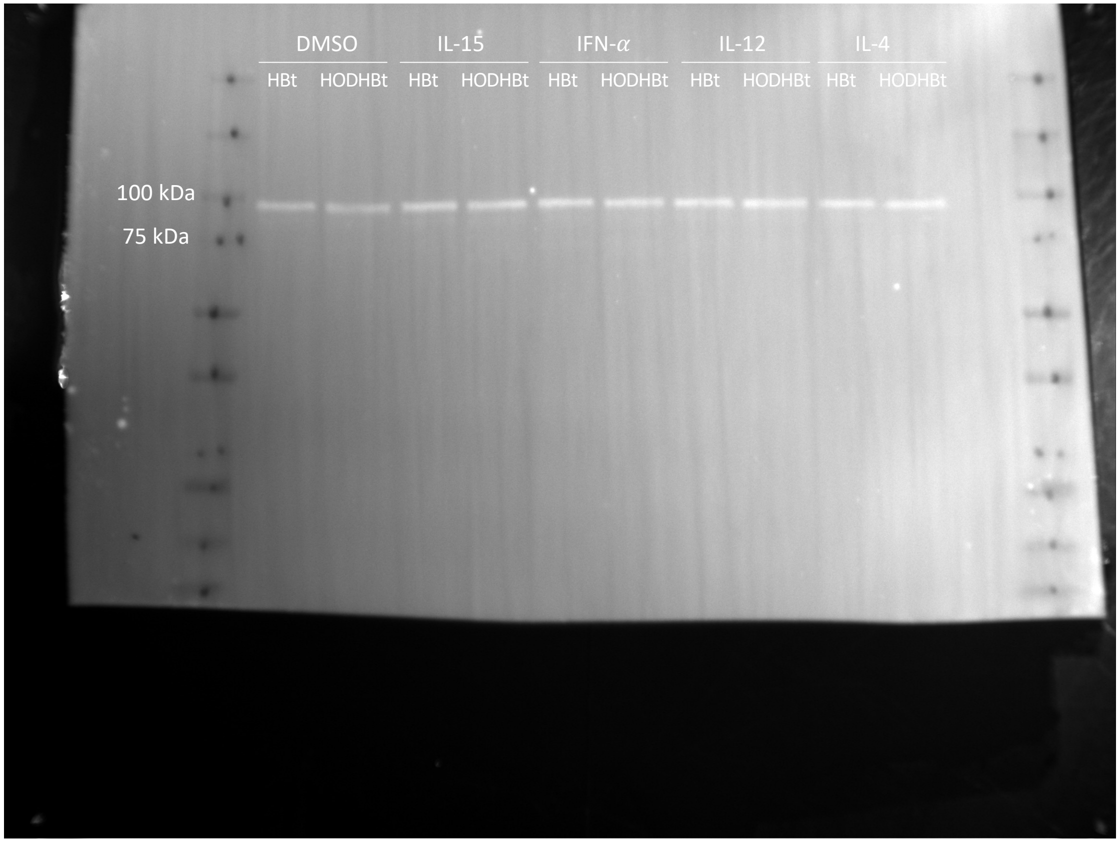

Full unedited blot for Figure 3B
STAT6 actin

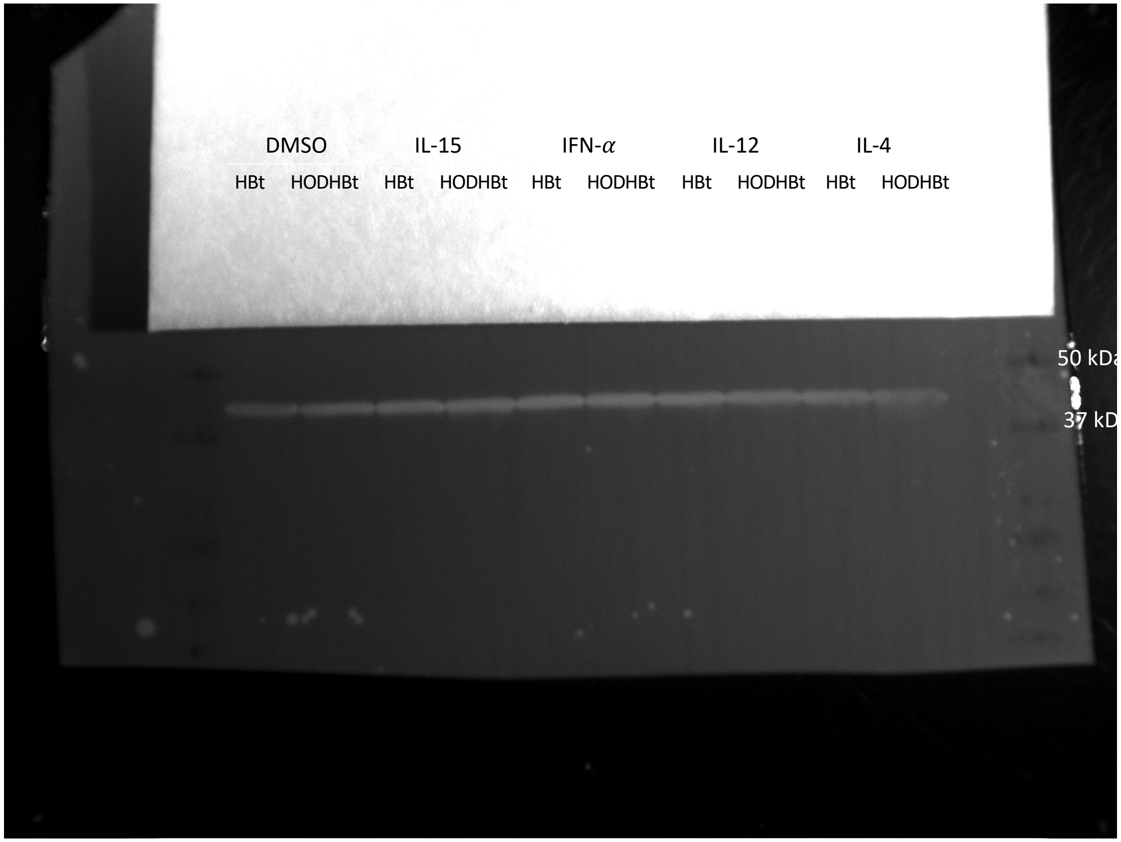

Full unedited blot for Figure 3D
pSTAT2

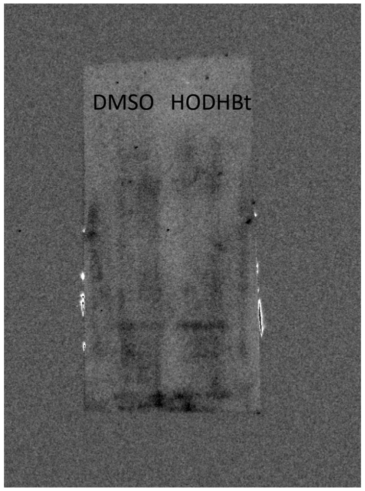

Full unedited blot for Figure 3D
V5-tag

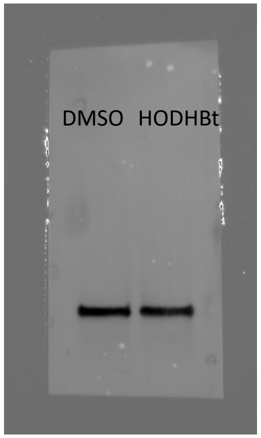

Full unedited blot for Figure 5C

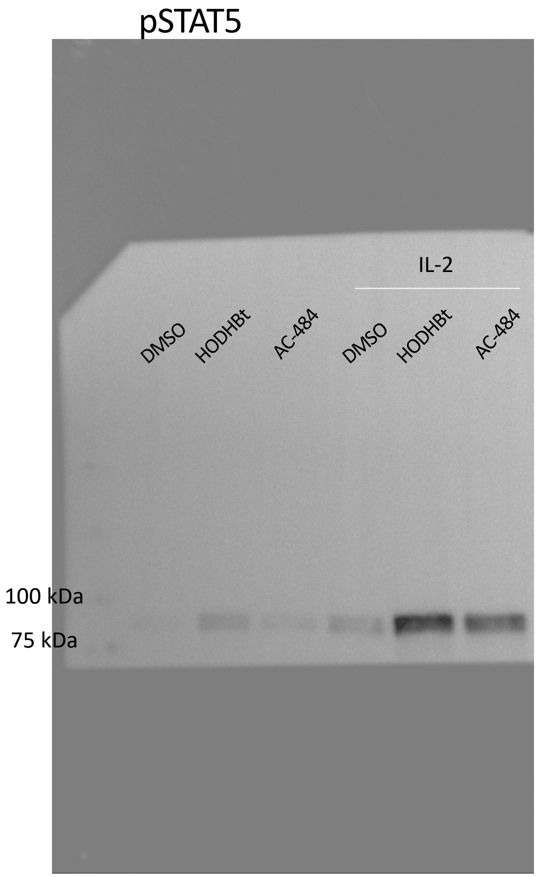

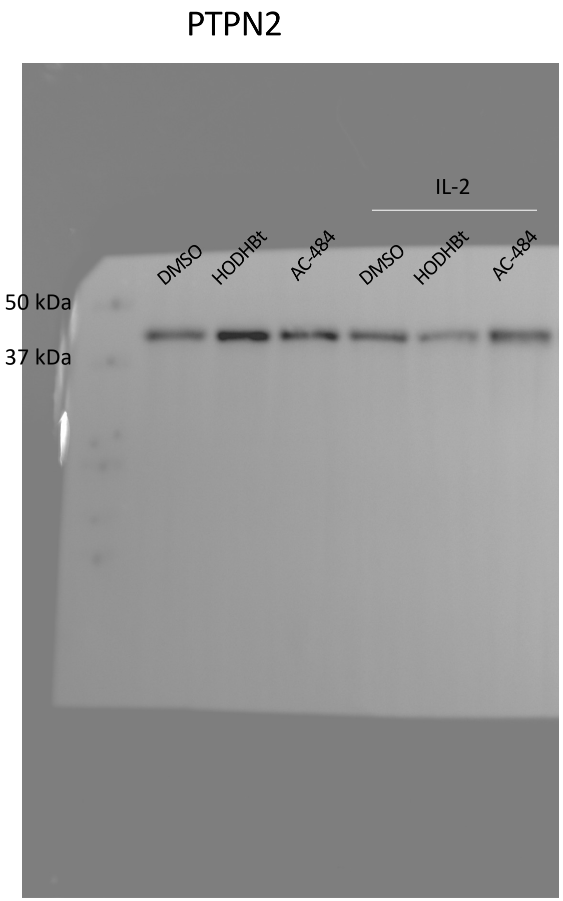

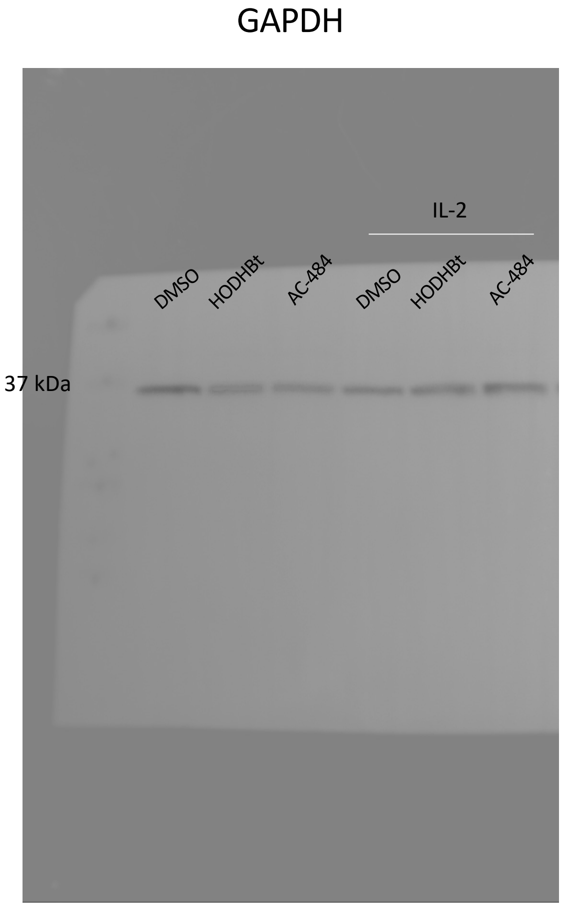

Full unedited blot for Figure 5C

STAT5

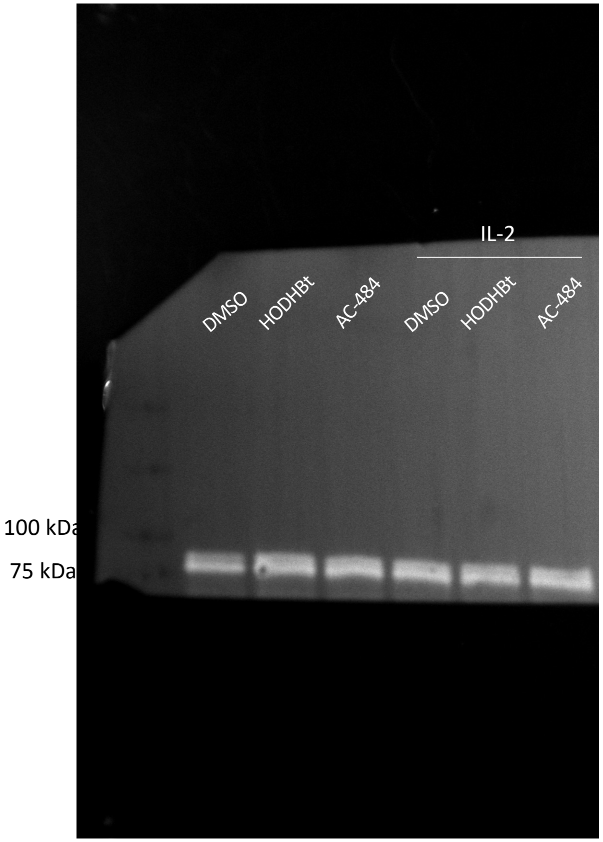

PTPN1

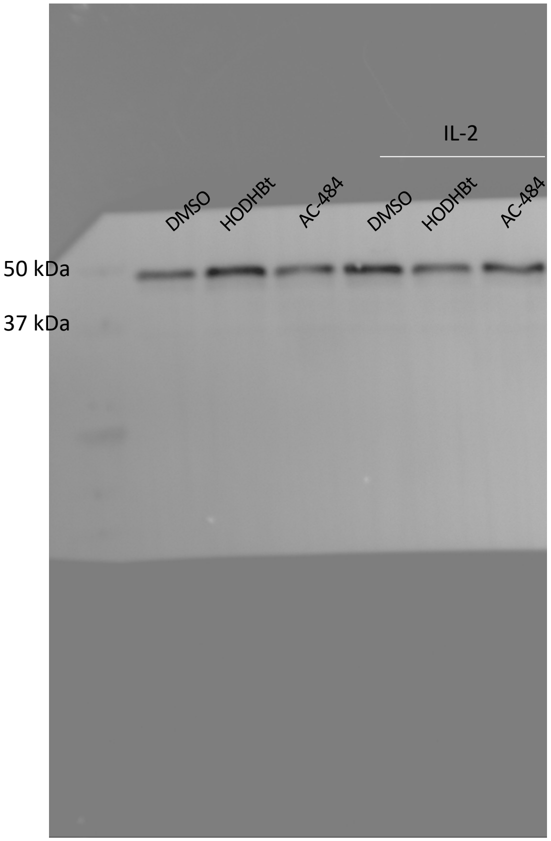

GAPDH

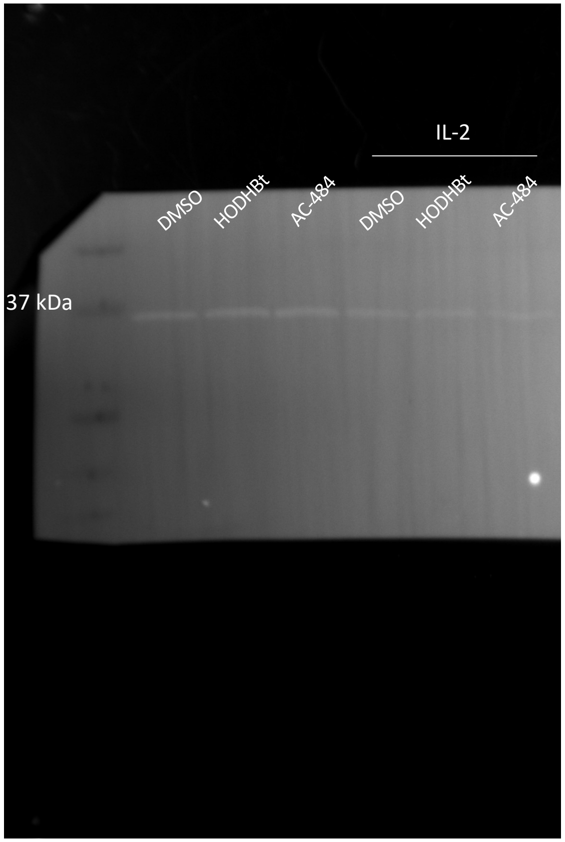

Full unedited blot for Figure 5D

pSTAT5

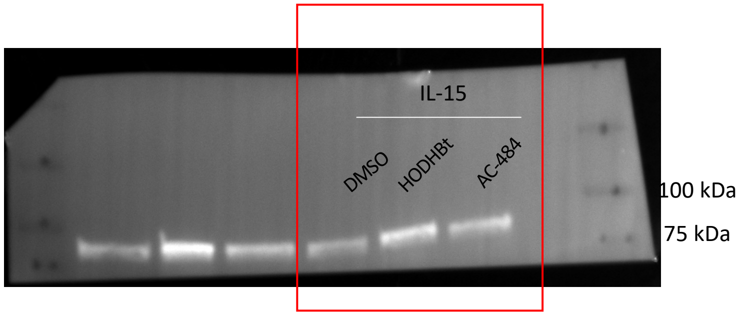

PTPN2

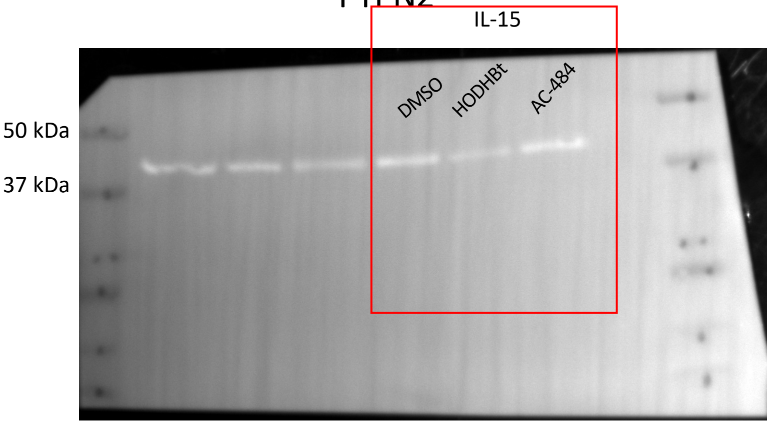

GAPDH

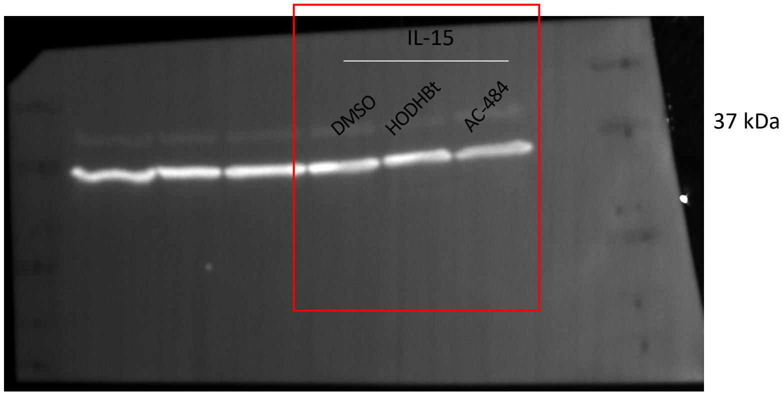

Full unedited blot for Figure 5D

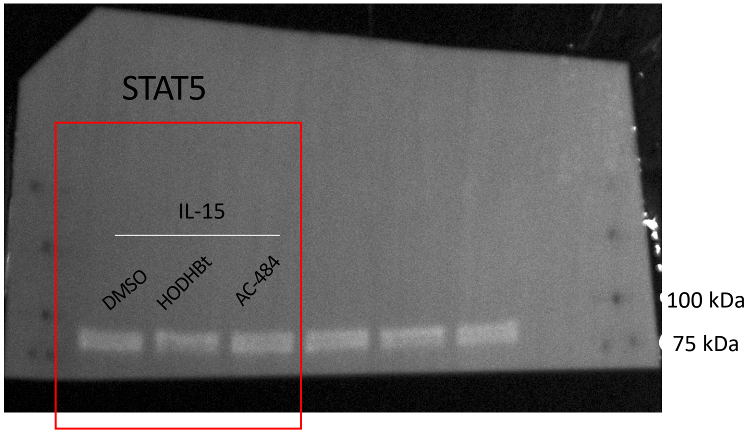

PTPN1

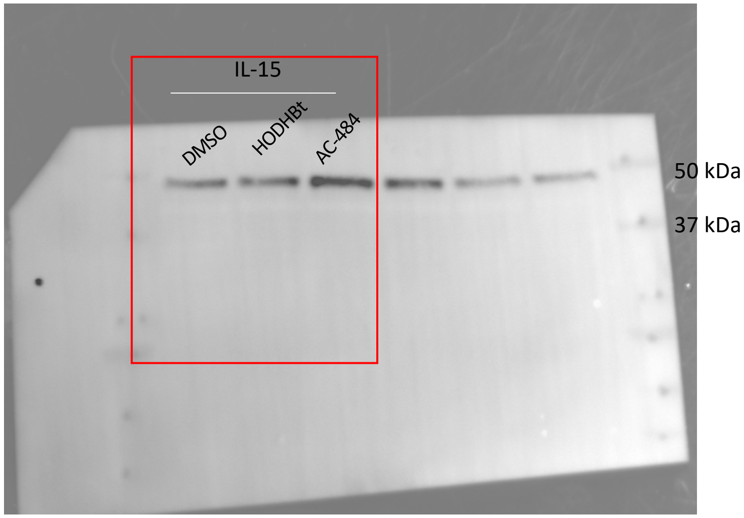

GAPDH

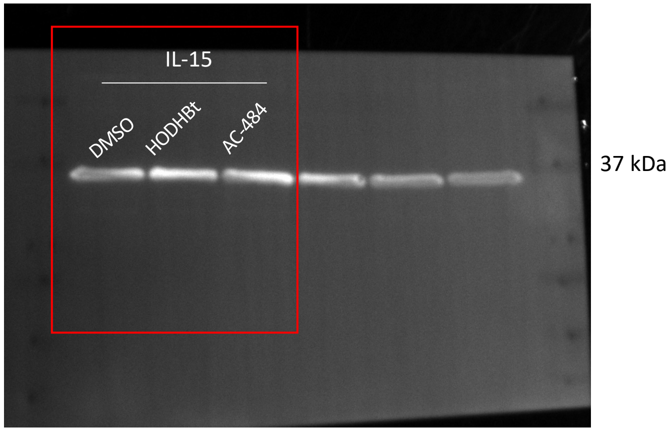

Full unedited blot for Supplemental Figure 2
RPL7A

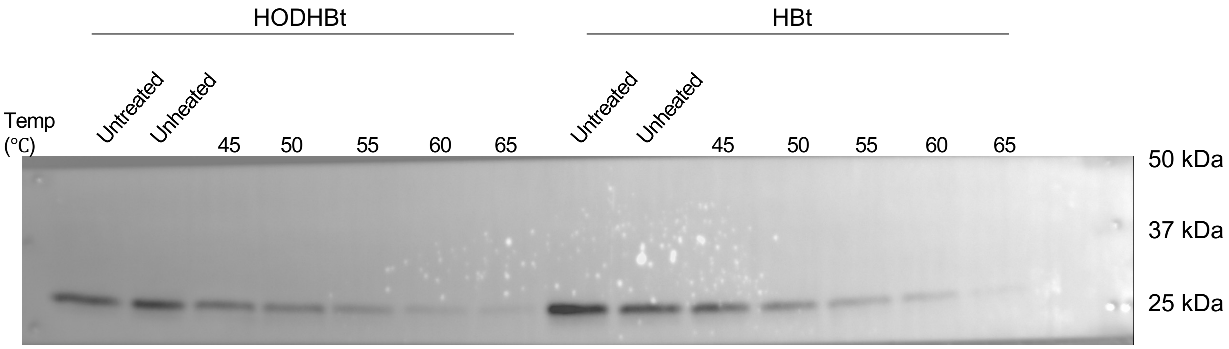

Full unedited blot for Supplemental Figure 2
STAT5

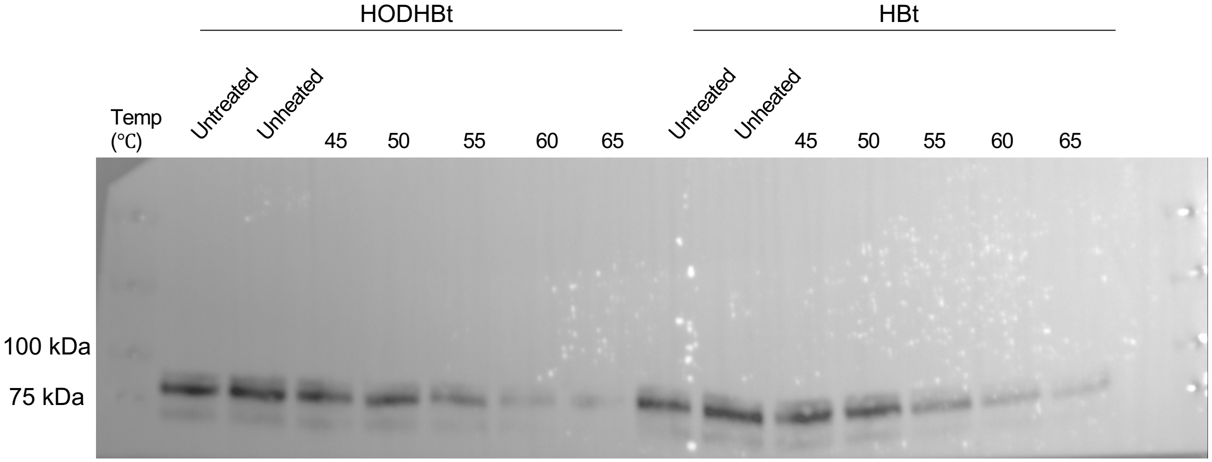

Full unedited blot for Supplemental Figure 2
actin

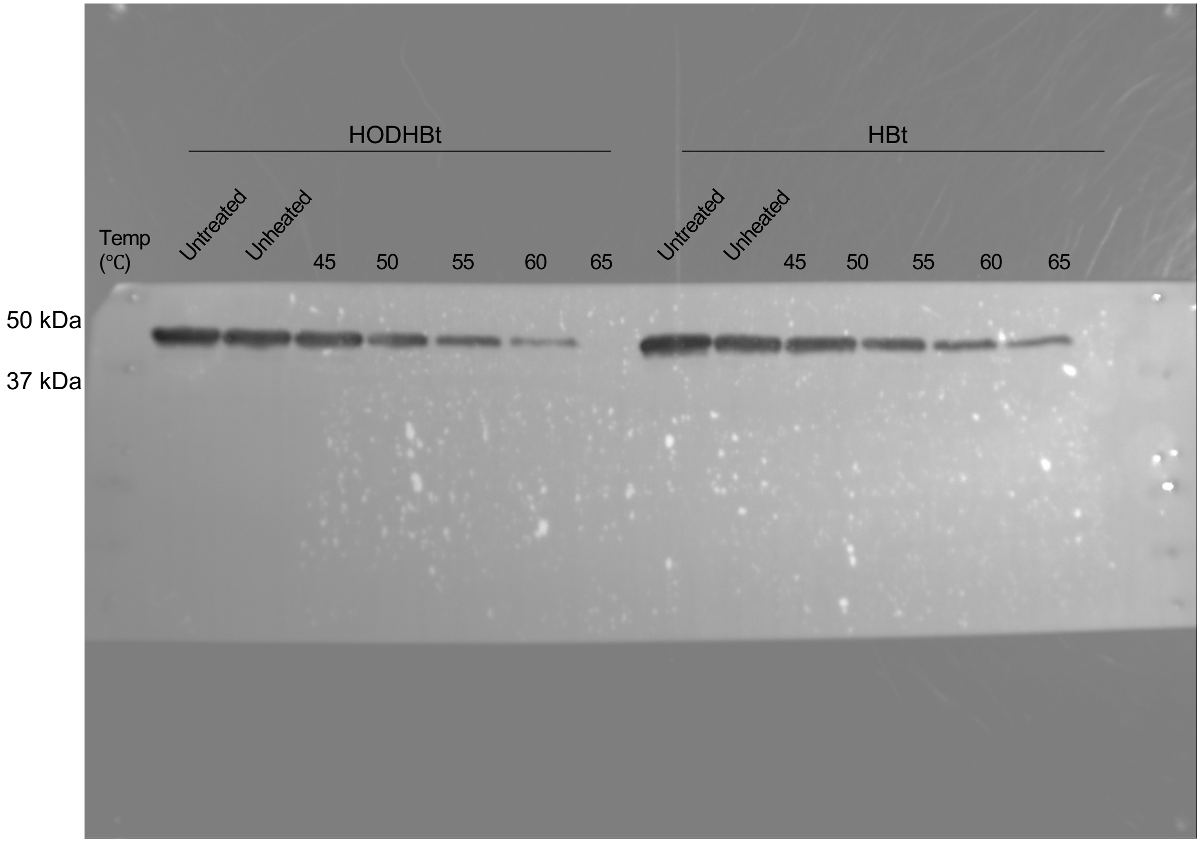

Full unedited blot for Supplemental Figure 2
CRKL

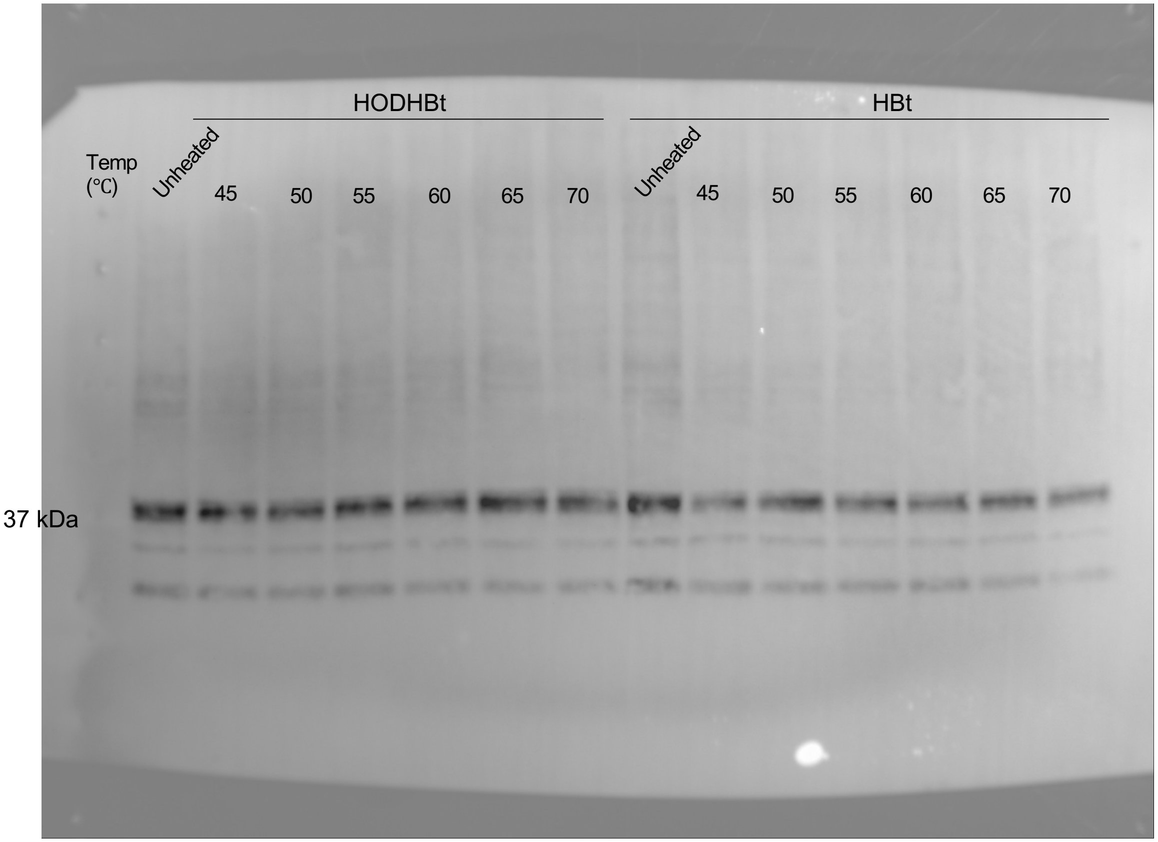

Full unedited blot for Supplemental Figure 2
PTPN1

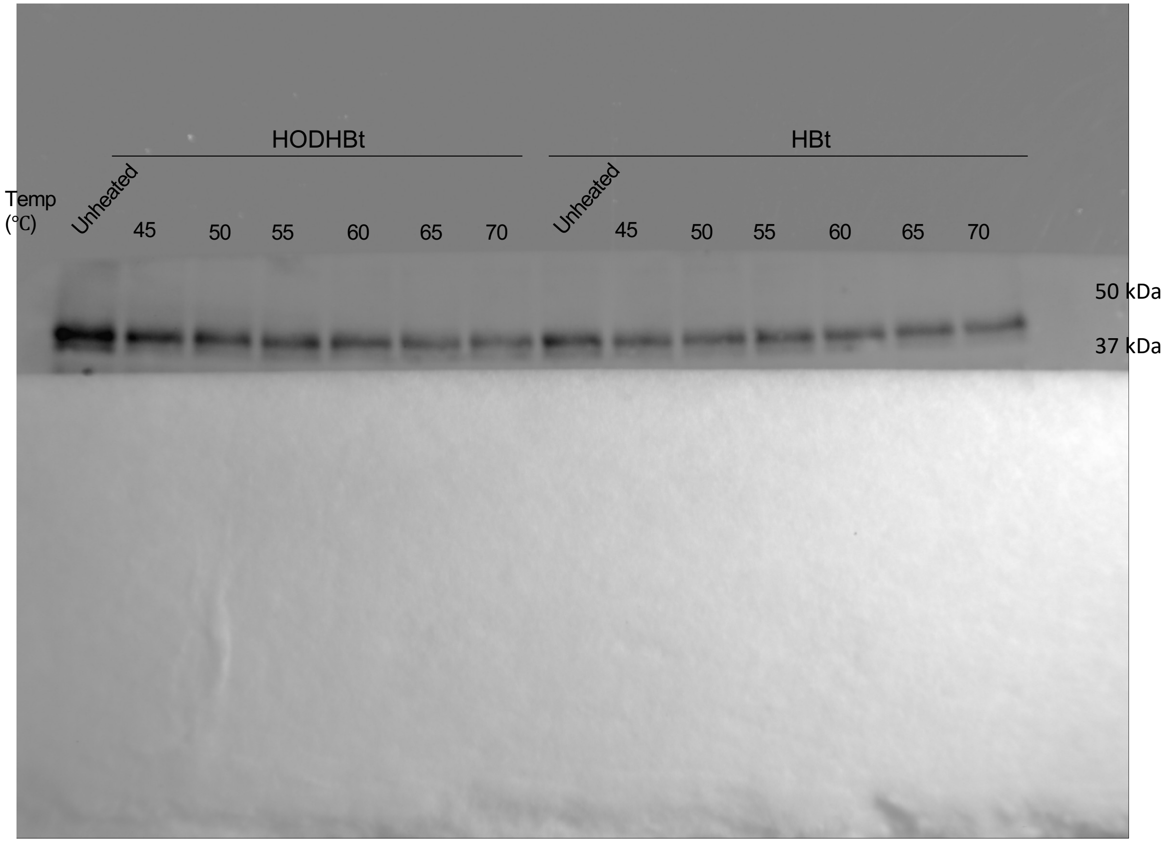

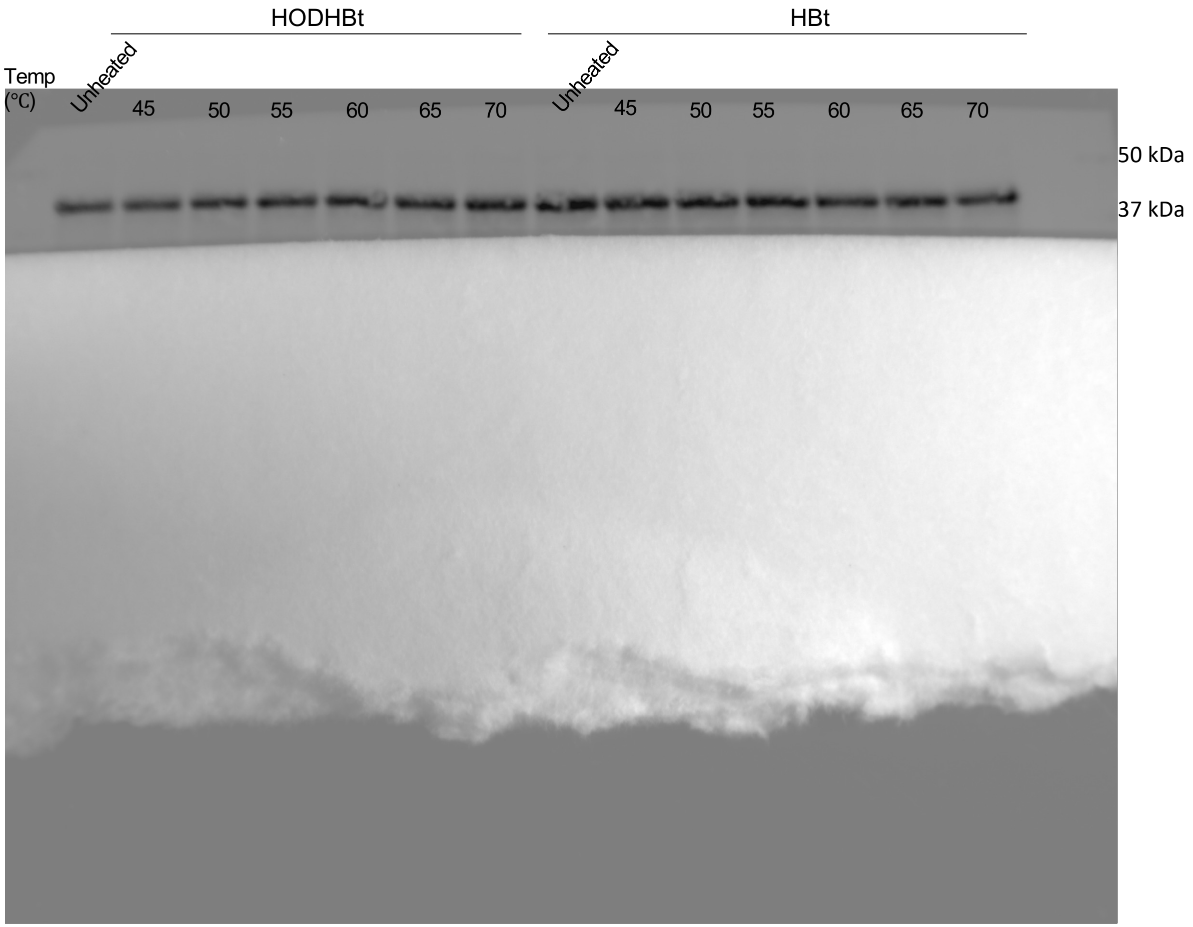

Full unedited blot for Supplemental Figure 2
PTPN2

Full unedited blot for Supplemental Figure 3D

pSTAT5

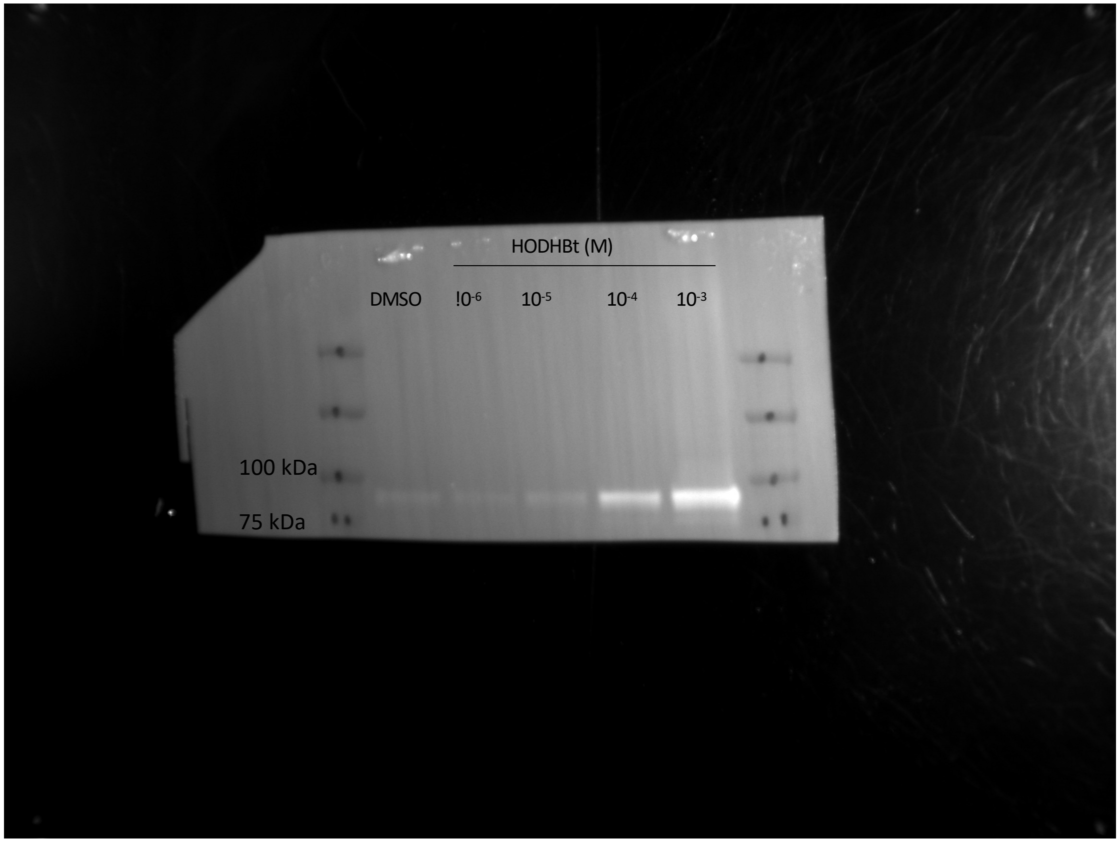

Full unedited blot for Supplemental Figure 3D
STAT5

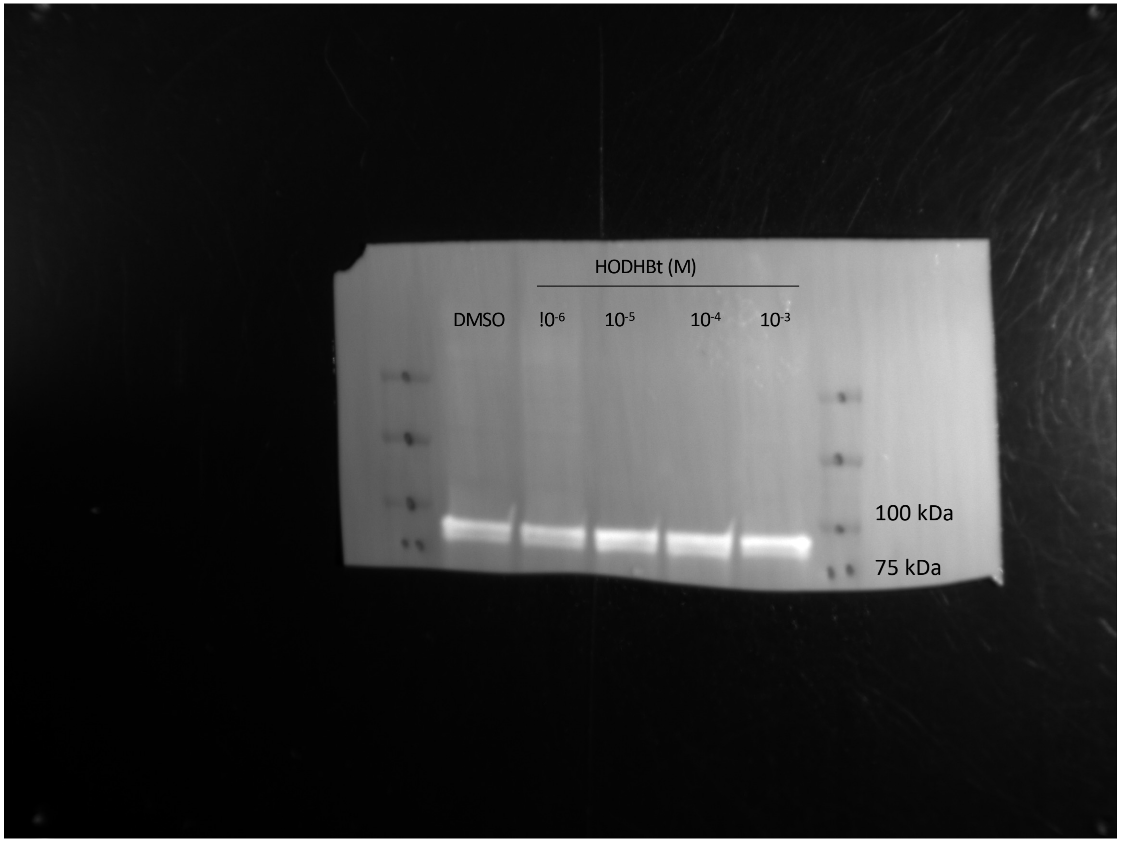

Full unedited blot for Supplemental Figure 3D
PTPN1

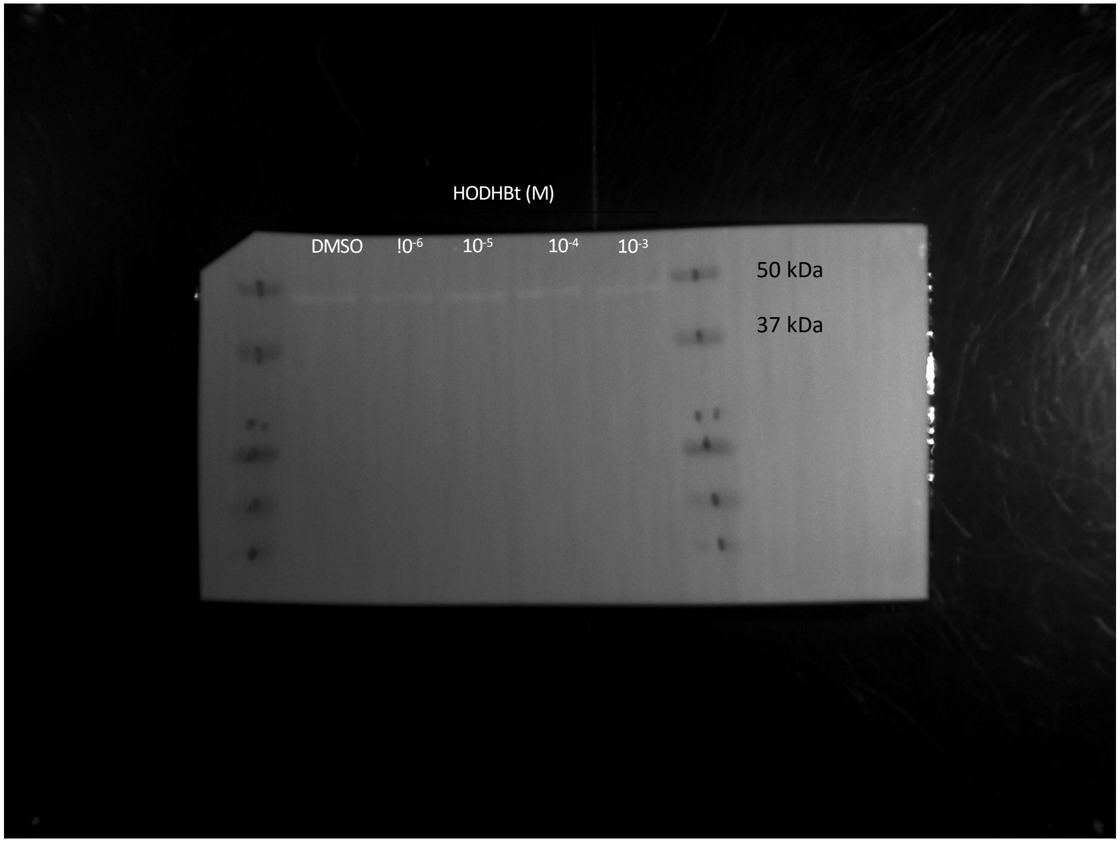

Full unedited blot for Supplemental Figure 3D
PTPN2

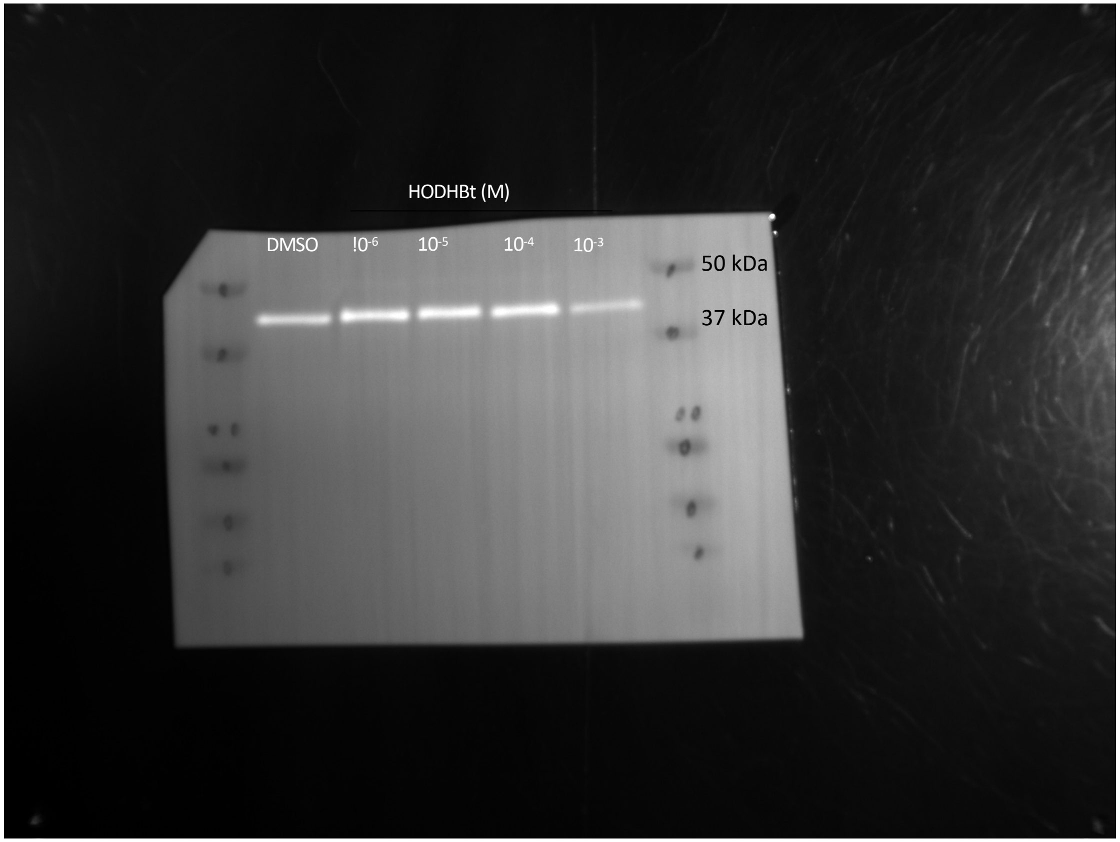

Full unedited blot for Supplemental Figure 3D
Actin

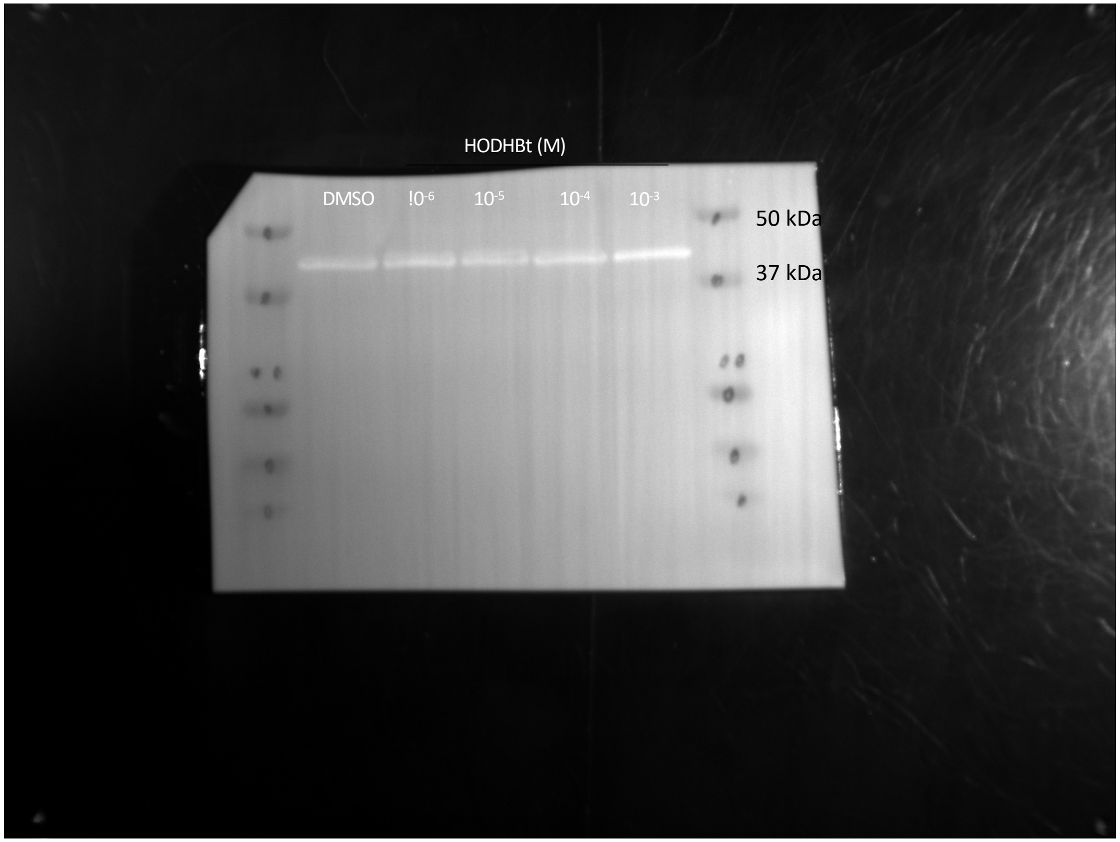

Supplement: Unedited blot and gel images [file jciinsight-9-179680-s107.pdf]
